# Variation among strains of *Borrelia burgdorferi* in host tissue abundance and lifetime transmission determine the population strain structure in nature

**DOI:** 10.1371/journal.ppat.1011572

**Published:** 2023-08-22

**Authors:** Christopher B. Zinck, Prasobh Raveendram Thampy, Eva-Maria E. Uhlemann, Hesham Adam, Jenny Wachter, Danae Suchan, Andrew D. S. Cameron, Ryan O. M. Rego, Dustin Brisson, Catherine Bouchard, Nicholas H. Ogden, Maarten J. Voordouw

**Affiliations:** 1 Department of Veterinary Microbiology, Western College of Veterinary Medicine, University of Saskatchewan, Saskatoon, Saskatchewan, Canada; 2 Vaccine and Infectious Disease Organization, University of Saskatchewan, Saskatoon, Saskatchewan, Canada; 3 Institute for Microbial Systems and Society, Faculty of Science, University of Regina, Regina, Saskatchewan, Canada; 4 Department of Biology, University of Regina, Regina, Saskatchewan, Canada; 5 Biology Centre, Institute of Parasitology, Czech Academy of Sciences, České Budějovice, Czech Republic; 6 Faculty of Science, University of South Bohemia, České Budějovice, Czech Republic; 7 Department of Biology, University of Pennsylvania, Philadelphia, Pennsylvania, USA; 8 Public Health Risk Sciences, National Microbiology Laboratory, Public Health Agency of Canada, St Hyacinthe, Quebec, Canada; 9 Groupe de recherche en épidémiologie des zoonoses et santé publique (GREZOSP), Faculté de Médecine Vétérinaire, Université de Montréal, Montreal, Canada; 10 Centre de recherche en santé publique (CReSP), Université de Montréal, Montreal, QC, Canada; University of Montana, UNITED STATES

## Abstract

Pathogen life history theory assumes a positive relationship between pathogen load in host tissues and pathogen transmission. Empirical evidence for this relationship is surprisingly rare due to the difficulty of measuring transmission for many pathogens. The comparative method, where a common host is experimentally infected with a set of pathogen strains, is a powerful approach for investigating the relationships between pathogen load and transmission. The validity of such experimental estimates of strain-specific transmission is greatly enhanced if they can predict the pathogen population strain structure in nature. *Borrelia burgdorferi* is a multi-strain, tick-borne spirochete that causes Lyme disease in North America. This study used 11 field-collected strains of *B*. *burgdorferi*, a rodent host (*Mus musculus*, C3H/HeJ) and its tick vector (*Ixodes scapularis*) to determine the relationship between pathogen load in host tissues and lifetime host-to-tick transmission (HTT). Mice were experimentally infected via tick bite with 1 of 11 strains. Lifetime HTT was measured by infesting mice with *I*. *scapularis* larval ticks on 3 separate occasions. The prevalence and abundance of the strains in the mouse tissues and the ticks were determined by qPCR. We used published databases to obtain estimates of the frequencies of these strains in wild *I*. *scapularis* tick populations. Spirochete loads in ticks and lifetime HTT varied significantly among the 11 strains of *B*. *burgdorferi*. Strains with higher spirochete loads in the host tissues were more likely to infect feeding larval ticks, which molted into nymphal ticks that had a higher probability of *B*. *burgdorferi* infection (*i*.*e*., higher HTT). Our laboratory-based estimates of lifetime HTT were predictive of the frequencies of these strains in wild *I*. *scapularis* populations. For *B*. *burgdorferi*, the strains that establish high abundance in host tissues and that have high lifetime transmission are the strains that are most common in nature.

## Author summary

For many pathogens, the benefit of higher abundance in host tissues is better transmission to uninfected hosts. Evidence for this relationship is rare because measuring pathogen transmission is difficult. Experimental infection with different pathogen strains in the same animal host allows scientists to test the relationship between abundance in host tissues versus transmission. We performed such a study using the tick-borne bacterium *Borrelia burgdorferi*, which causes Lyme disease in humans in North America. Laboratory mice were infected with 1 of 11 different strains of *B*. *burgdorferi* and ticks were fed on these mice to measure host-to-tick transmission. We determined the presence and abundance of *B*. *burgdorferi* in the mouse tissues and in the ticks using basic molecular methods. We found that strains with higher abundance in the mouse tissues had higher lifetime transmission to feeding ticks. We used published databases to obtain approximate estimates of the frequencies of our strains in nature. We found that strains that had higher transmission in the laboratory were also more common in nature. Studies that compare strain fitness between the lab versus nature can help scientists understand the evolution of multi-strain pathogens in response to human control measures.

## Introduction

Pathogen evolution is determined by the trade-offs between key life history traits including replication, transmission, persistence (*i*.*e*., avoidance of immune clearance by the host), and virulence [1–6]. Pathogen strains that have higher within-host replication (*i*.*e*., establish higher abundance in host tissues) are expected to have higher transmission success and/or lower clearance by the host, but they may reduce the survival of the host (*i*.*e*., have higher virulence), which shortens the duration of the infection. Pathogens are therefore expected to evolve intermediate virulence, which balances the benefits of higher transmission with the costs of a shorter lifespan of infection [6–10]. Life history theory assumes that variation in these key pathogen traits and their trade-offs are determined at least in part by pathogen genetics [1–6]. A powerful approach for demonstrating the genetic basis of trade-offs is the comparative method, which requires measurement of pathogen traits across a set of genetically distinct pathogen clones or strains in a common host and a common environment [4,8,11,12].

Numerous empirical studies have reported the expected positive relationship between pathogen abundance (or pathogen load) in the host tissues and pathogen transmission [7–10,13–15]. However, upon closer inspection, only one study demonstrates the presence of a genetic correlation between these two pathogen traits by measuring them across a set of parasite clones in experimentally infected hosts [12]. Studies on human pathogens have demonstrated the expected positive relationship but cannot control for host or environmental factors [7,8]. Some studies using arthropod hosts as model systems have measured genetic variation among pathogen strains in load or virulence but not in transmission [9,10]. Thus, there are surprisingly few examples of a genetic correlation between pathogen load in host tissues and pathogen transmission and recent reviews have highlighted the need for more such studies [4,6,15].

Studies that measure pathogen life history traits are typically conducted in the lab under carefully controlled conditions. An open question is whether these lab-based estimates of strain fitness are relevant to the real world. The revolution in next generation sequencing methods has allowed scientists to document pathogen strain diversity in host populations with great accuracy and precision [16–20]. All else being equal, the frequency of a strain in the pathogen population is a measure of its fitness at a point in time; common strains are expected to have higher fitness compared to rare strains. Thus, one way to validate laboratory-based estimates of pathogen strain fitness would be to test whether they can explain variation in frequencies among strains in the real world [18,20]. Such validation would increase our confidence that lab-based estimates of the genetic variation underlying pathogen life history traits are relevant to their evolution in nature.

Lyme borreliosis is the most common vector-borne disease in Europe and North America [21] and is caused by tick-borne spirochetes belonging to the *Borrelia burgdorferi* sensu lato (sl) genospecies complex. The three most important *Borrelia* genospecies are *B*. *afzelii* and *B*. *garinii* in Europe, and *B*. *burgdorferi* sensu stricto (hereafter *B*. *burgdorferi*) in North America [22]. These genospecies are transmitted among vertebrate hosts through the bite of *Ixodes* ticks, which include *I*. *scapularis* in North America and *I*. *ricinus* in Europe. *Ixodes* ticks consist of three feeding developmental stages: larva, nymph, and adult. Larval ticks acquire spirochetes after feeding on infected hosts and moult into infected nymphs, which infect the next generation of vertebrate hosts. Adult female ticks feed on deer that are incompetent for *B*. *burgdorferi* sl pathogens [23,24]. In summary, the two transmission steps that maintain *B*. *burgdorferi* sl in nature are from infected vertebrate hosts to naïve larvae and from infected nymphs to naïve vertebrate hosts.

Each *B*. *burgdorferi* sl genospecies comprises multiple strains that coexist in natural populations. These strains can vary in their life history traits, such as replication or abundance in host tissues [25–29] or host-to-tick transmission (HTT) [30–37]. However, it is generally thought that *B*. *burgdorferi* sl does not elicit pathology in important rodent reservoir hosts like the white-footed mouse (*Peromyscus leucopus*), which in most studies (but not all), appears to be tolerant of infection [38–41]. Few studies have investigated covariation between life history traits for any *B*. *burgdorferi* sl pathogen. A study of two strains of *B*. *afzelii* found that the strain abundance in host tissues was correlated with HTT [26]; but the sample size (n = 2 strains) was not sufficient to test for genetic correlations between pathogen life history traits. Other studies have found that lab-based estimates of spirochete load in host tissues or lab-based estimates of transmission are associated with strain frequencies in nature [18,25,42]. However, no study has investigated the relationships between host tissue spirochete load, lifetime HTT, and frequency in nature across a set of *B*. *burgdorferi* sl strains. Such studies enhance our understanding of the ecological and evolutionary forces that shape the current strain structure, and which strains are most likely to be of public health significance.

In this study, we investigated the variation in host-to-tick transmission (HTT) among 11 different strains of *B*. *burgdorferi*. We have previously shown that there is significant variation in host tissue abundance among these strains [42]. The present study had three objectives. First, characterize the variation in lifetime HTT among 11 strains of *B*. *burgdorferi*. Second, quantify the relationship between host tissue spirochete load (replication) and lifetime HTT for 11 strains of *B*. *burgdorferi*. Third, determine whether our laboratory estimates of strain-specific lifetime HTT are correlated with strain frequencies in nature. We hypothesize that strains with higher tissue spirochete load will have a greater lifetime HTT. Finally, we predict that the strains with a greater lifetime HTT will be more common in nature.

## Materials and methods

### Ethics statement

The use of mice and all procedures in this study were reviewed and approved by the Animal Research Ethics Board (AREB), which is part of the University Animal Care Committee (UACC) at the University of Saskatchewan. The AREB-assigned animal use protocol number for our study was 20190012. The UACC follows the animal experimentation guidelines of the Canadian Council on Animal Care (CCAC).

### Strains, ticks, and mice

For this study, 11 low passage strains of *B*. *burgdorferi* were obtained from the isolate collection of the Public Health Agency of Canada (PHAC; Table 1). To minimize plasmid loss during culture, the *B*. *burgdorferi* strains had undergone only four passages from the time of isolation from field-collected *I*. *scapularis* ticks to needle inoculation into our mice. Initially, 12 strains were used, however, one failed to infect mice and is not included here [42]. These strains were originally isolated from wild *I*. *scapularis* ticks collected from field sites in Canada [43]. Strains were whole genome sequenced and their multi-locus sequence types (MLSTs) and *ospC* types were determined by PHAC [43]. The strains were selected by maximizing MLST diversity while also considering the *ospC* type and geographic region of origin. We selected 5 strains from Eastern Canada (Nova Scotia), and 6 from Midwestern Canada (Manitoba and western Ontario) because previous work found substantial differences in strain diversity between these two regions [44–46]. All strains were validated for the presence of plasmids that are necessary for the natural transmission cycle of *B*. *burgdorferi* [42].

**Table 1:** The genetic identity and location of origin are shown for the 11 strains of *B*. *burgdorferi* used in the study. Strain identity numbers (Bb16-) are from the reference publication [43]. Counts of larvae and nymphs are only for the subset of infected mice as mice that failed to become infected were excluded from analysis.

PubMLST Strain ID	NML Strain ID	MLST	*ospC* type	Region	Town (Province)	Infected mice	Infected larvae	Infected nymphs
2388	10–2	32	C3	Midwest	Roseau River (MB)	8/8	62/87	202/234
2392	22–2	55	A	Midwest	Buffalo Point (MB)	8/8	53/83	176/226
2038	54	741	I	Midwest	Buffalo Point (MB)	8/8	33/93	155/230
2439	57–1	29	A3	Midwest	Buffalo Point (MB)	8/8	70/90	212/230
2443	66	237	J	Midwest	Buffalo Point (MB)	7/8	27/77	114/210
2399	126	43	N	Midwest	Big Grassy (ON)	8/8	58/90	210/236
2413	150	19	E	East	Lunenberg (NS)	8/8	61/94	205/239
2416	167	12	M	East	Shelburne (NS)	8/8	66/93	200/240
2418	174	37	T	East	Lunenberg (NS)	5/8	47/58	148/150
2420	178–1 ^a^	3	K	East	Lunenberg (NS)	8/8	85/89	233/236
2429	198	3	K	East	Bedford (NS)	8/8	83/96	229/240
	Total					84/88	645/950 ^c^	2,084/2,471 ^c^
	Uninfected ^b^	NA	NA	NA		0/20	0/200	0/558

^a^ This strain was originally intended to be 178–2 but turned out to be 178–1 (Tyler et al., 2018)[43], thus we have two isolates of the same strain in this study (MLST 3, *ospC* K).

^b^ Uninfected control mice were fed upon by uninfected *I*. *scapularis* nymphs. As expected, all the ticks that fed on the uninfected control mice tested negative for *B*. *burgdorferi*.

^c^ The larvae and nymphs from the 4 mice that failed to become infected following the nymphal challenge are not included in these numbers

Laboratory-reared, specific-pathogen-free *I*. *scapularis* larvae were obtained from the National Tick Research and Education Resource at Oklahoma State University. This colony is regularly outbred with wild *I*. *scapularis* ticks to prevent inbreeding and is screened for multiple tick-borne pathogens including *B*. *burgdorferi*. Six batches of larvae were purchased over the course of the study (2 temporal blocks x 3 larval infestations per block). The larvae for each infestation came from egg clutches of different adult female ticks.

Specific-pathogen-free, 6 to 8-week-old, female and male *Mus musculus* C3H/HeJ mice were purchased from The Jackson Laboratory and used as the reservoir host in this study. This strain of mouse has been used in numerous experimental infection studies with *B*. *burgdorferi* [28,29,47]. *Mus musculus* mice are not natural reservoir hosts for *B*. *burgdorferi*. The main reasons for working with this non-natural reservoir host were availability, convenience, cost, and genetic similarity of individual mice. While strain C3H/HeJ possesses a mutation in Toll-like receptor 4, which makes it highly susceptible to Gram-negative bacteria compared to the wild-type C3H/HeN, previous studies have found that C3H/HeJ mice are no different than wild type in their response to infection with *B*. *burgdorferi* [48,49].

### Creation of *I*. *scapularis* nymphs infected with one of the 11 strains of *B*. *burgdorferi*

The *I*. *scapularis* nymphs were infected with one of the 11 strains as described previously [42]. Briefly, each strain was cultured from frozen stock in Barbour-Stonner-Kelly-H (BSK-H) medium (Millipore Sigma, Oakville, Canada) for 9–14 days. C3H/HeJ mice were then needle-inoculated with single strains (n = 2–4 mice per strain). At 2–4 weeks post-infection (PI), mice were infested with naïve *I*. *scapularis* larvae that were left to feed to repletion following our infestation procedure (see below). These larvae were maintained at room temperature in humidity chambers to moult into nymphs.

### Infecting mice via nymphal tick bite with one of the 11 strains of *B*. *burgdorferi*

A total of 108 mice (54 female, 54 male) were used in this study. For each of the 11 strains, 8 mice (4 female, 4 male) were infected, with an additional 20 mice (10 female, 10 male) being uninfected controls. To mimic a natural infection cycle, experimental mice were infected by allowing three putatively infected *I*. *scapularis* nymphs to feed to repletion as previously described [42]. Control mice were infested with 3 uninfected *I*. *scapularis* nymphs. To manage the workload, the study was run in two orthogonal temporal blocks (A, B) processed ~6 months apart; each block contained half the experimental mice and half the control mice. To confirm the infectious challenge, replete nymphs were recovered and tested for the presence of *B*. *burgdorferi* by qPCR targeting the *23S rRNA* gene of *B*. *burgdorferi* (see below).

### Infestations of mice with *I*. *scapularis* larvae

At days 30, 60, and 90 PI, mice were infested with 50–100 naïve *I*. *scapularis* larval ticks that were allowed to feed to repletion. All mice were anaesthetized for ~20 min with xylazine and ketamine for each infestation, and larvae were brushed onto their fur. Mice were housed in a cage setup that facilitated the collection of engorged larvae as described previously [27]. For each mouse and infestation, 5 engorged larvae were frozen immediately at -80°C following recovery (no engorged larvae were collected for the first infestation of the mice in block A). The remaining engorged larvae were kept at high humidity (~99%) and room temperature and were allowed to moult into nymphs. Four weeks after the engorged larvae moulted into nymphs, a sample of 10 nymphs per mouse per infestation were frozen at -80°C for DNA extraction. These nymphs will hereafter be referred to as unfed nymphs or flat nymphs to indicate that they have not taken their nymphal blood meal.

### Collection of ear biopsies and tissue samples from mice at necropsy

The sampling of the ear biopsies and necropsy tissue samples from the mice in this study was previously described in [42]. Ear biopsies (2 mm in diameter) were collected from the right ear on days 29, 59, and 89 PI, which were the days before the mice were infested with *I*. *scapularis* larvae. The mice were euthanized on day 97 PI, and we dissected and collected 7 necropsy tissues: (1) ventral skin, (2) left ear, (3) right ear, (4) heart, (5) bladder, (6) right rear tibiotarsal joint, and (7) kidney. Mouse necropsy tissue samples were frozen at -80°C prior to DNA extraction.

### DNA extraction of whole *I*. *scapularis* ticks

For each mouse, there were 45 ticks (3 infestations * (5 engorged larvae + 10 flat nymphs moulted from larvae fed on the mouse) per infestation) for a total of 4,860 ticks (108 mice x 45 ticks). Tick extractions were done as previously described [27]. Ticks were homogenized using bead beating with the Qiagen TissueLyser II. Homogenized ticks were digested with proteinase K overnight at 56°C in a shaking incubator at 400 rpm. DNA was extracted from the homogenized ticks using the Qiagen DNEasy 96-well plate extraction kit following the manufacturer’s instructions. The DNA extractions of the ticks were eluted with 65 μL of elution buffer and stored at -80°C prior to use in qPCR.

### DNA extraction of mouse ear biopsies and necropsy tissues

The homogenization and DNA extraction of the mouse ear biopsies and necropsy tissue samples in this study were previously described in [42]. Briefly, a total of 324 ear biopsies (108 mice x 3 ear biopsies per mouse) and 756 mouse tissue samples from necropsies were processed (108 mice x 7 tissue samples per mouse); the 7 necropsy samples included (1) ventral skin, (2) left ear, (3) right ear, (4) heart, (5) bladder, (6) right rear tibiotarsal joint, and (7) kidney. Whole ear biopsies and the necropsy samples (~20 mg of tissue) were homogenized using 3.6-mm stainless steel beads with the Qiagen TissueLyser II or a micro pestle. Homogenized tissue samples were digested with proteinase K overnight at 56°C in a shaking incubator at 400 rpm. DNA was extracted from homogenized tissue samples using the Qiagen DNEasy Blood and Tissue kit individual spin columns following the manufacturer’s instructions. The DNA extractions of the ear biopsies and necropsy tissues were eluted with 55 μL and 100 μL of elution buffer, respectively, and stored at -80°C prior to use in qPCR.

### Detection of *B*. *burgdorferi* in *I*. *scapularis* ticks by qPCR

To test for the presence of *B*. *burgdorferi* in experimental samples, a qPCR targeting the *23S rRNA* intergenic spacer of *B*. *burgdorferi* was used [50]. Every plate was run with a synthetic gene fragment to use as a standard [42]. A strict lower detection limit of 1 gene copy/μL was used as a cut-off based on the lower detection limit of the standards. To generate copy numbers, a single regression was done on multiple runs of standards (range: 1–10^5^ copies/μL, n = 81) so that the same equation was used for every transformation [42]. As whole ticks were used in every DNA extraction, values are reported as the log_10_ *23S rRNA* copies per tick.

In comparison to nymphs, replete larvae have a lower abundance of *B*. *burgdorferi* and the presence of mammalian blood can inhibit amplification of DNA [51,52]. For this reason, a qPCR targeting the *I*. *scapularis* calreticulin gene was developed (**see ESM Section 1**) to determine DNA extraction success by using a tick housekeeping gene present in all samples. As with the *23S rRNA* qPCR, a synthetic gene fragment was designed to use as a standard (**see ESM Section 2**).

### Detection of *B*. *burgdorferi* in mouse tissues by qPCR

The abundance of *B*. *burgdorferi* in the mouse ear biopsies and necropsy tissue samples was also estimated using *23S rRNA* qPCR and was standardized relative to the number of mouse *Beta-actin* gene copies, as previously described [42].

### Statistical methods

#### Infection prevalence and spirochete load of mouse tissues

The infection prevalence and the spirochete load of the mouse ear biopsies and the 7 mouse tissue samples were analyzed as described in **ESM Section 3** (see also [42]). The infection prevalence refers to whether the mouse tissue is uninfected (0) or infected (1). In the present study, the spirochete load in the mouse tissues was calculated as log10[(*23S rRNA* gene copies/10^6 *Beta-actin* copies) + 1]. The advantage of this log[X + 1] transformation is that uninfected tissues are defined and have a value of 0. The mouse tissue spirochete loads were scaled to z-scores with mean of 0 and a variance of 1 (hereafter referred to as the z-score spirochete loads). The advantage of this scaling is that each tissue has the same weight in estimates that are averaged across tissues.

#### Overview of transmission measures: LIP, LSL, NIP, and NSL

We analyzed 4 measures of transmission of *B*. *burgdorferi*: (1) proportion of engorged larvae infected with *B*. *burgdorferi*, hereafter larval infection prevalence (LIP), (2) abundance of *B*. *burgdorferi* in the subset of infected larvae, hereafter larval spirochete load (LSL) (3) proportion of unfed nymphs infected with *B*. *burgdorferi*, hereafter nymphal infection prevalence (NIP), and (4) abundance of *B*. *burgdorferi* in the subset of infected unfed nymphs, hereafter nymphal spirochete load (NSL). The details of these analyses are in **ESM Sections 4, 5, 6, and 7**.

#### Analysis of *B*. *burgdorferi* infection prevalence in larvae and nymphs

For larvae and nymphs, the presence of *B*. *burgdorferi* infection was a binomial variable (0 = uninfected, 1 = infected). The LIP and the NIP were analyzed by generalized linear mixed-effects models (GLMMs) with binomial errors. For both analyses, the fixed factors included strain (11 levels; see Table 1), mouse sex (2 levels: female, male), infestation (3 levels: days 30, 60, 90 PI), and their interactions. Temporal block (2 levels: A, B) was included as a main effect. Mouse identity was included as a random factor. Analyses were restricted to ticks from the subset of 84 mice that became infected following nymphal challenge (see below). To generate the final models, interactions were sequentially removed if they were not significant and/or if their inclusion prevented the GLMM from converging on precise parameter estimates. Factor significance was estimated using type II Wald tests, post-hoc analyses were performed and estimated marginal means (EMMs) were calculated.

#### Analysis of *B*. *burgdorferi* abundance in infected larvae and infected nymphs

For the subset of infected larvae and nymphs, analyses were run using linear mixed-effects models (LMMs). For both analyses, the fixed factors included strain, mouse sex, infestation, and their interactions. Temporal block was included as a main effect. Mouse identity was included as a random factor. For the larvae only, calreticulin copies per tick were also included as a covariate to control for random variation in the efficiency of DNA extraction. Non-significant interactions were sequentially removed to generate the final models. Post-hoc analyses and EMMs were performed as mentioned above.

#### Relationship between the mouse tissue spirochete loads and NIP

We wanted to determine which mouse tissues were most strongly associated with variation in the NIP (**see ESM Section 8 for details**). There were 8 mouse tissues: ear biopsies that were sampled the day before the larval infestation (i.e., days 29, 59, and 89 PI) and 7 mouse tissues that were sampled at necropsy (day 97 PI). Each mouse tissue had an estimate of spirochete load, which was calculated as log10[(*23S rRNA* gene copies/10^6 *Beta-actin* copies) + 1]. The mouse tissue spirochete loads were scaled to z-scores with a mean of 0 and a variance of 1 (hereafter referred to as the z-score spirochete loads).

We used AIC-based model selection to compare 20 GLMMs with binomial errors; each model analyzed the NIP as a function of a different mouse tissue variable (**see ESM Section 8 for details**), as well as the fixed factors of mouse sex, infestation, and relevant interactions. Mouse identity was included as a random factor. Models were ranked according to their corrected Akaike Information Criterion (AICc) where the best models have the lowest AICc scores. The parameter estimates of the best models were analyzed in more detail.

#### Correlation matrix of mouse tissue spirochete loads and *B*. *burgdorferi* transmission variables

We wanted to visualize the pairwise correlation matrix for our eight estimates of tissue spirochete load: (1) ear biopsy, (2) ventral skin, (3) left ear, (4) right ear, (5) heart, (6) bladder, (7) kidney, (8) and right rear tibiotarsal joint, and our four estimates of host-to-tick transmission: (9) larval infection prevalence (LIP), (10) larval spirochete load (LSL), (11) nymphal infection prevalence (NIP), and (12) nymphal spirochete load (NSL). The genetic correlation matrix was calculated across the 11 *B*. *burgdorferi* strains, whereas the phenotypic correlation matrix was calculated across the 84 infected mice (**see ESM Section 9**).

#### Frequencies of *B*. *burgdorferi* strains in *I*. *scapularis* tick populations in North America

We wanted to use the frequencies of our *B*. *burgdorferi* strains in *I*. *scapularis* tick populations in North America as a measure of strain fitness. Two studies by Travinsky et al [53] and Ogden et al [46] determined the frequencies of *B*. *burgdorferi* MLSTs in *I*. *scapularis* ticks collected in the USA and Canada. Travinsky et al [53] classified MLSTs as belonging to the northeastern or north central (hereafter midwestern) USA. Although Ogden et al [46] sampled *I*. *scapularis* ticks in Canada, they similarly classified the MLSTs as being the same as, or clustering with, MLSTs that had been previously detected in the northeastern or midwestern USA. Our study contained 11 *B*. *burgdorferi* strains with 10 unique MLSTs (strain 178–1 and strain 198 both have MLST 3). We obtained frequency estimates for our strains by matching their MLSTs to the MLSTs (without regard to *ospC* type) in these published data sets (**see ESM Section 10 for details**).

MLSTs from regions not relevant to our study (e.g., northern California and Europe) were excluded. After this step, Travinsky et al [53] identified 35 different MLSTs in 654 *I*. *scapularis* ticks and Ogden et al [46] identified 40 different MLSTs in 166 *I*. *scapularis* ticks. Together, these two studies contained 8 of the 10 unique MLSTs and 9 of the 11 strains in the present study. The two strains that were not detected in either study, strains Bb16-66 and Bb16-54 with MLSTs 237 and 741, were assigned counts of zero. Strains Bb16-10-2 and Bb16-57-1 had matching MLSTs in the study by Travinsky et al [53] but different *ospC* types. For each study, we counted the number of *I*. *scapularis* ticks infected with each of the 10 unique MLSTs of interest. To obtain the best estimate of the MLST-specific frequencies in *I*. *scapularis* populations, the counts were combined for the two studies.

For the two studies combined, the 10 unique MLSTs in our study were detected in 144 and 109 ticks in northeastern and midwestern North America, respectively (total n = 253 ticks). For the two studies combined, 40 MLSTs were identified in 482 and 338 ticks in northeastern and midwestern North America, respectively (total n = 820 ticks). These counts were converted to four different strain-specific frequencies of which two are presented here (**see ESM Sections 10 and 11**). For frequency 2 (%), the counts for the 10 MLSTs were divided by the number of ticks when the two geographic regions were combined (n = 820). For frequency 4 (%), the counts for the 4 MLSTs in the northeast and the 6 MLSTs in the Midwest were divided by the number of ticks in each region (n = 482 and n = 338). Thus, frequency 4 accounts for potential differences in sampling effort between geographic regions, whereas frequency 2 does not.

### Statistical software

The data analyzed for this manuscript can be accessed at Dryad [54]. We used R version 4.0.4 for all statistical analyses [55]. The list of R packages and R functions we used are given in **ESM Section 1**.

## Results

### Infestations of mice and recovery and testing of immature *I*. *scapularis* ticks

For each strain, 5–8 infected mice survived to the end of the study with a total of 84 infected mice surviving (**Table 1**). Of the 4 mice that did not become infected following nymphal challenge, 3 had been infected with strain Bb16-174 and 1 with Bb16-66 (**Table 1**). In addition, 20 uninfected control mice survived to the end of the study. For each mouse and each infestation, we froze a maximum of 5 engorged larvae and 10 unfed nymphs. The infection status of these mice was previously determined by performing qPCR on 3 post-infection ear biopsies and 7 tissues collected at necropsy and by performing ELISA on 2 post-infection blood samples per mouse [42]. In the present study, a maximum of 45 immature ticks (15 larvae, 30 nymphs) were tested for their infection status for each mouse. Every infected mouse was found positive for *B*. *burgdorferi* in mouse tissues, xenodiagnostic ticks, and with respect to the IgG antibody response on a commercial ELISA. No evidence of *B*. *burgdorferi* infection was found in the 20 control mice, or in the 4 mice that failed to demonstrate infection after the infectious nymphal challenge. In summary, there was no ambiguity about the infection status of the 84 infected mice, the 4 experimental mice that failed to become infected following nymphal challenge, and the 20 uninfected control mice.

### Infection prevalence and spirochete load of mouse tissues

The infection prevalence and the spirochete loads of the 3 ear biopsies and the 7 necropsy tissues are presented in detail in **ESM Section 3** (see also [42]). The infection prevalence in the biopsies from the right ear on days 29, 59, and 89 PI were 98.8%, 100.0%, and 91.7%, respectively. For the necropsy tissues on day 97 PI, the infection prevalence was low in skin-related tissues (52.4% in ventral skin, 38.1% in left ear, and 14.3% in right ear) and was high in internal tissues (92.9% in heart, 97.6% in bladder, and 71.4% in right rear tibiotarsal joint).

The spirochete loads are measured in units of *23S rRNA* copies per 10^6 *Beta-actin* copies. For the biopsies of the right ear, the mean spirochete load decreased by 71.8% from the first to the third biopsy (2878.5 versus 812 units). For the necropsy tissues on day 97 PI, if the right ear is taken as the reference, the mean spirochete load in the kidney, left ear, ventral skin, joint, bladder, and heart is 3.2x, 8.0x, 14.3x, 32.6x, 76.9x, 824.2x, and 1182.9x higher, respectively. Thus, the mean spirochete load in the skin-related organs (ventral skin, ears) was much lower compared to the internal organs (bladder, and heart).

An unexpected result is that the infection prevalence in the right ear decreased from 91.7% in the biopsy on day 89 PI to 14.3% in the necropsy sample on day 97 PI (a 6.4-fold decrease). Similarly, the spirochete load in the right ear decreased from 812 units in the biopsy on day 89 PI to 1.3 units in the necropsy sample on day 97 PI (a 624.6-fold decrease). Of note, all ear biopsies were collected 1 day before infestation with larvae. However, ear tissue samples collected at necropsy on day 97 PI were collected one week after larvae fed on the mice.

### Larval infection prevalence

Each mouse had up to 5 engorged larvae frozen from three separate infestations at days 30, 60, and 90 PI. In total, 950 and 200 engorged larvae were recovered and tested from the 84 infected mice and 20 uninfected control mice, respectively. The infection prevalence in the engorged larvae that fed on the 84 infected mice was 67.9% (645/950) with none of the 200 larvae from the 20 uninfected control mice testing positive (Table 1).

The analysis of the LIP found that strain (p = 1.346*10^−15^) and infestation (p = 3.359*10^−5-^) were significant (**see ESM Section 4**). The overall LIP varied 2.7-fold among strains (strain Bb16-54: 35.5%; strain Bb16-178-1: 95.5%). The LIP decreased from the first infestation at day 30 PI to the subsequent infestations (30 days PI: 89.7%; 60 days PI: 60.5%; 90 days PI: 64.6%).

### Larval spirochete load

The analysis of the LSL for the subset of infected engorged larvae (n = 645) found that strain (p = 5.968*10^−12^) and infestation (p = 4.773*10^−11^) were both significant (**see ESM Section 5**). The LSL has units of *23S rRNA* copies/larva and varied 11.9-fold between the lowest and highest abundance strains (strain Bb16-54: 1,317.4 units; strain Bb16-178-1: 15,635.5 units). As with the LIP, the LSL was highest in the first infestation (30 days PI: 10,501.0 units; 60 days PI: 2,115.3 units; 90 days PI: 5,538.3 units).

### Nymphal infection prevalence (host-to-tick transmission)

Each mouse had up to 10 unfed, 4-week-old nymphs frozen from the three separate infestations at days 30, 60, and 90 PI. In total, 2,471 and 558 unfed nymphs were tested from the 84 infected mice and 20 uninfected control mice, respectively. The infection prevalence in the unfed nymphs that had fed as larvae on the 84 infected mice was 84.3% (2,084/2,471) with none of the 558 nymphs that had fed as larvae on the 20 uninfected control mice testing positive (Table 1).

**Fig 1:**
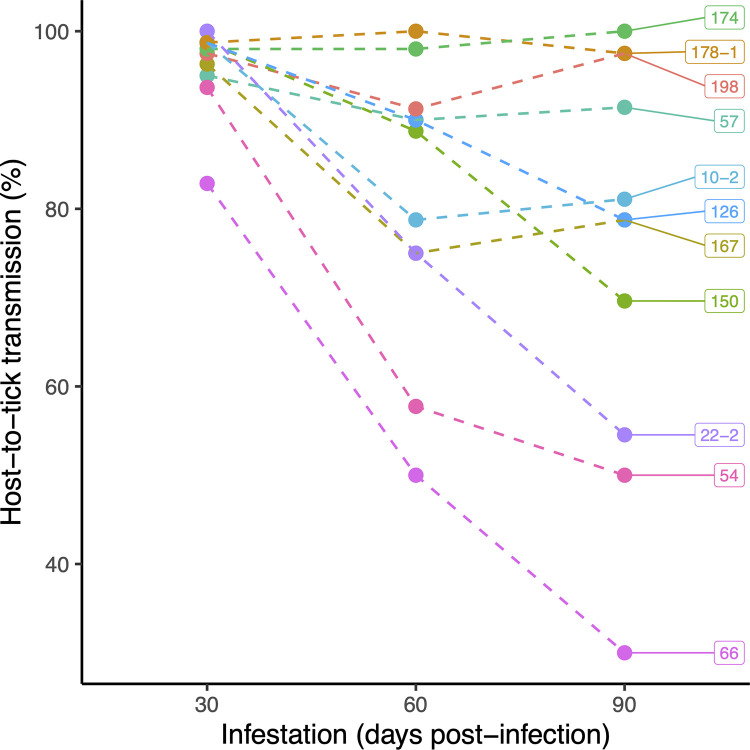
The transmission of *B*. *burgdorferi* from infected mice to immature *I*. *scapularis* ticks decreased over the 3 successive larval infestations for some strains but remained constant over time for other strains. Lifetime host-to-tick transmission (HTT) was measured by infesting mice with *I*. *scapularis* larvae on 3 occasions (days 30, 60, and 90 post-infection), allowing the engorged larvae to moult into nymphs, and testing the nymphs for infection with *B*. *burgdorferi*. Each of the 33 estimates of HTT (11 strains x 3 infestations) was based on a maximum of 80 unfed nymphs (8 mice per strain x 10 unfed nymphs per infestation). Labels on datapoints are strain ID numbers.

**Fig 2:**
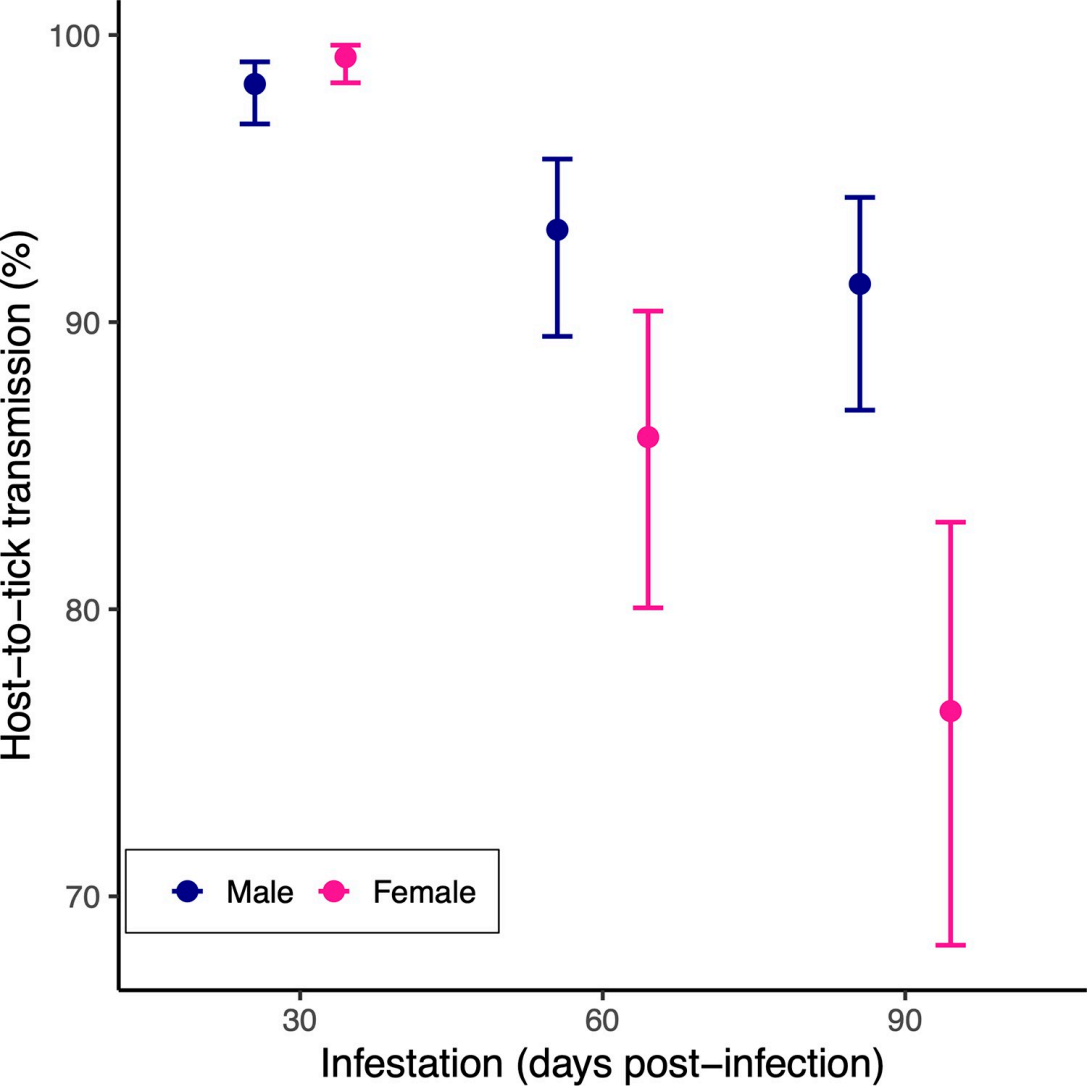
Host-to-tick transmission of *B*. *burgdorferi* from infected male and female C3H/HeJ mice to immature *I*. *scapularis* ticks decreased over the course of the infection. Male mice had higher lifetime HTT compared to female mice for all 11 strains of *B*. *burgdorferi*. Lifetime HTT was measured by infesting mice with *I*. *scapularis* larvae on 3 occasions (days 30, 60, and 90 post-infection), allowing the engorged larvae to moult into nymphs, and testing the nymphs for infection with *B*. *burgdorferi*. The highly significant 2-way interaction between mouse sex and infestation (p = 0.0002) indicates that the decrease in the HTT over time was larger in the female mice compared to the male mice.

The analysis of the NIP found that strain (p = 1.758*10^−14^), mouse sex (p = 0.001), infestation (p = 2.2*10^−16^), and the 2-way interaction between sex and infestation (p = 1.625*10^−4^) were significant (**see ESM Section 6**). For 8 of the 11 strains, the NIP decreased with successive larval infestations, whereas it remained consistently high (above 90%) for 3 strains (Bb16-174, Bb16-178-1, Bb16-198; **Fig 1**). At day 30 PI, the NIP for female and male mice was similar (females: 97.8%; males: 94.8%) but declined more for females by day 90 PI (females: 67.1%; males: 82.6%; **Fig 2**). Thus, the NIP decreased over time in both sexes, but the decrease in NIP was significantly greater for female mice compared to male mice (**Fig 2**). Overall, the lifetime NIP was 87.8% (1,113/1,268) in male mice (n = 43), which was higher compared to 80.7% (971/1,203) in female mice (n = 41). The lifetime NIP was higher for male mice compared to female mice for 9 of 11 *B*. *burgdorferi* strains.

### Nymphal spirochete load

The analysis of the NSL in the subset of infected unfed nymphs (n = 2,084) found that strain (p = 2.2*10^−16^), infestation (p = 8.914*10^−12^), and the 2-way interactions between strain and infestation (p = 7.561*10^−5^), and between mouse sex and infestation (p = 0.026) were all significant (**see ESM Section 7**). The NSL has units of *23S rRNA* copies/nymph and varied 8.6-fold between the lowest and highest abundance strains (strain Bb16-66: 7,707.3 units; strain Bb16-174: 66,069.3 units). Unlike the LSL, the NSL in the unfed nymphs increased for some strains with successive infestations, whereas it decreased for others. Like the NIP, the NSL in ticks fed on female mice decreased more with successive infestations than the NSL in ticks fed on male mice (Males: 30 days PI: 26,525.2 units; 90 days PI: 19,075.1 units; Females: 30 days PI: 32,587.7 units; 90 days PI: 17,937.3 units).

### Relationship between the mouse tissue spirochete loads and NIP

The AIC-based model selection of the 20 GLMMs to determine which mouse tissues influenced the NIP is presented in **ESM Section 8**. The top 5 GLMMs of the NIP had 99.2% of the support. These 5 models included spirochete loads for the (1) heart, (2) bladder, (3) mean of heart and bladder, (4) ear biopsy, and (5) infection prevalence for the heart with 30.7%, 29.7%, 26.7%, 7.5% and 4.7% of the support, respectively. The GLMMs that modelled the NIP as a function of heart spirochete load or ear biopsy spirochete load are discussed below.

**Fig 3:**
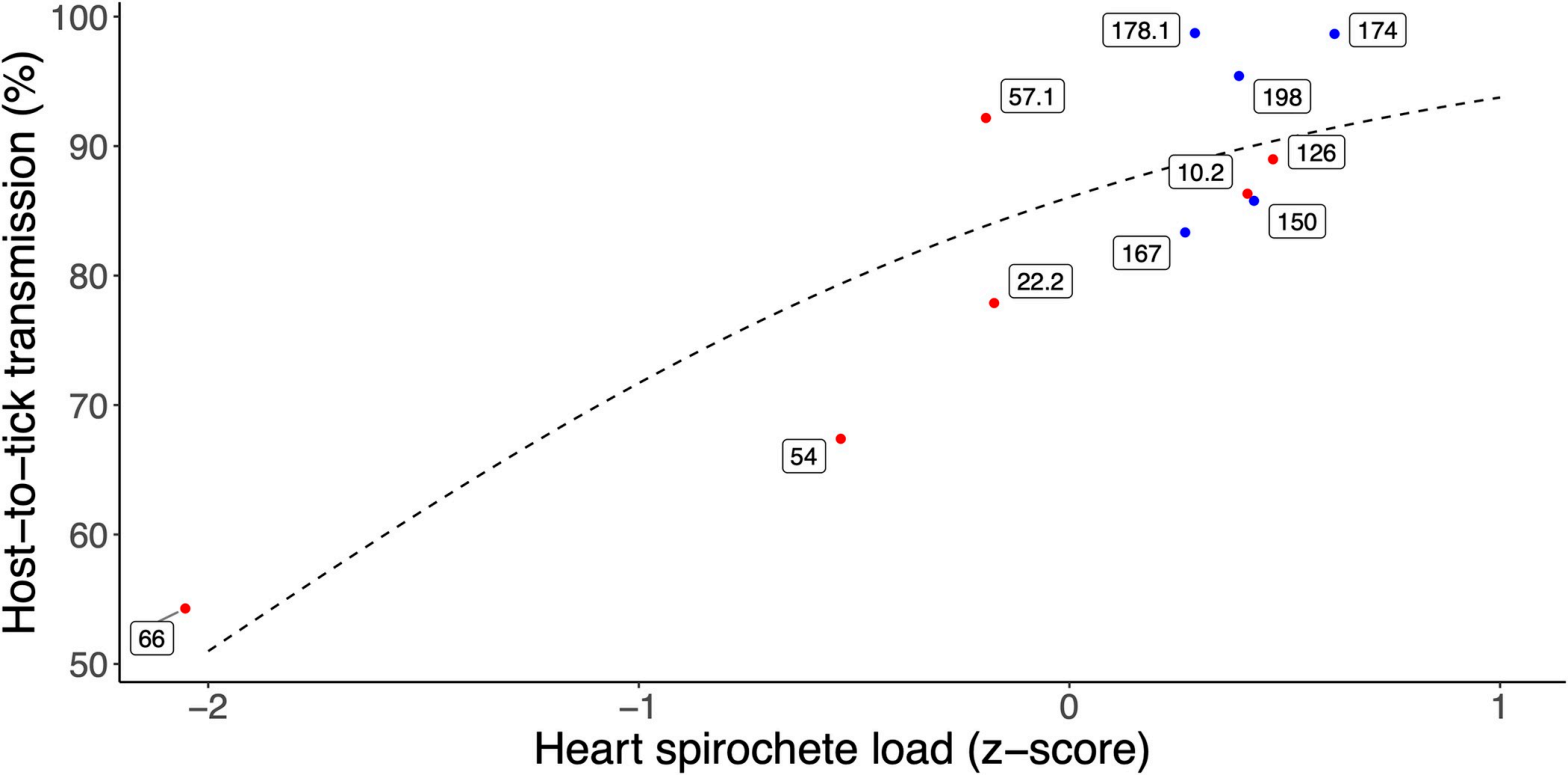
The mean spirochete load in the mouse heart determines the lifetime host-to-tick transmission (HTT) across the 11 strains of *B*. *burgdorferi*. The lifetime HTT (also called the lifetime NIP) is the percentage of nymphs infected with *B*. *burgdorferi*; these nymphs had taken their larval blood meal on the mice at days 30, 60, or 90 PI. The lifetime HTT for each strain was calculated by summing the infected nymphs and uninfected nymphs over all infestations and mice. The hearts were dissected from the euthanized mice at day 97 PI. The spirochete load in the mouse heart was calculated as log10[(*23S rRNA*/ 10^6^ *Beta-actin*) + 1] and was converted to a z-score. A GLM with quasibinomial errors found a strong positive relationship between heart spirochete load and lifetime HTT across the 11 *B*. *burgdorferi* strains (slope = 0.890, SE = 0.214, p = 0.002). Strains from eastern and midwestern Canada are shown in blue and red, respectively. Labels on datapoints are strain ID numbers.

The GLMM that modelled the NIP as a function of heart spirochete load, mouse sex, and infestation is described in **ESM Section 8**. The heart spirochete load had a significant and positive effect on the NIP (slope = 0.751, SE = 0.164, p < 0.001). This result indicates that *B*. *burgdorferi* strains that establish higher spirochete loads in the heart tissues have higher host-to-tick transmission to feeding ticks. To visualize the relationship, we graphed the mean lifetime NIP for each strain versus its mean heart spirochete load (**Fig 3**). The lifetime NIP was calculated by summing the infected and uninfected nymphs over all infestations and mice for each of the 11 *B*. *burgdorferi* strains. There was a strong positive relationship between heart spirochete load and lifetime NIP across the 11 strains of *B*. *burgdorferi* (**Fig 3;** slope = 0.890, SE = 0.214, p = 0.002).

**Fig 4:**
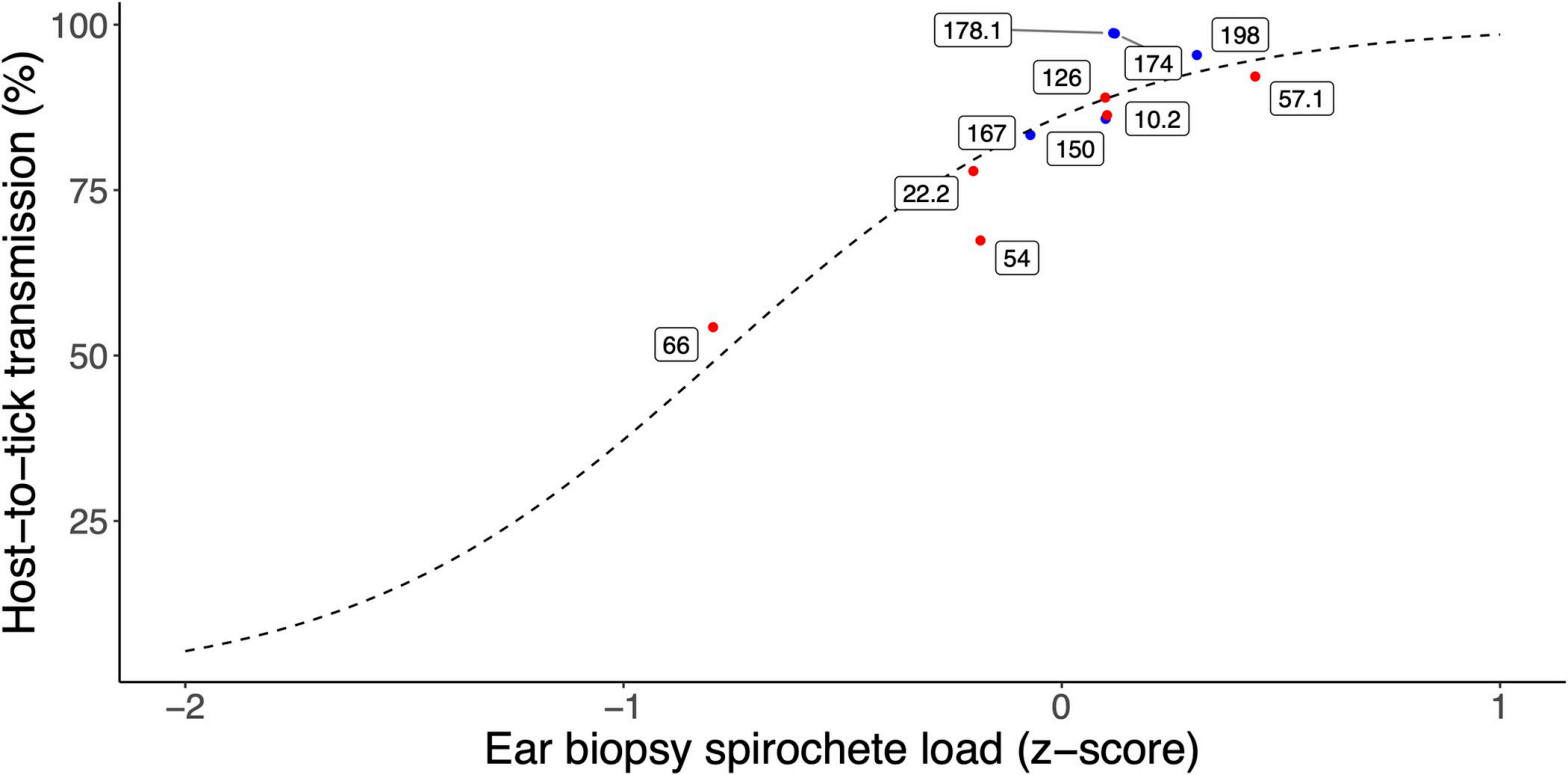
The mean spirochete load in the mouse ear biopsy determines the lifetime host-to-tick transmission (HTT) across the 11 strains of *B*. *burgdorferi*. The lifetime HTT (also called the lifetime NIP) is the percentage of nymphs infected with *B*. *burgdorferi*; these nymphs had taken their larval blood meal on the mice at days 30, 60, or 90 PI. The lifetime HTT for each strain was calculated by summing the infected nymphs and uninfected nymphs over all infestations and mice. The ear biopsies were sampled on days 30, 60, and 90 PI and were averaged for each mouse and strain. The spirochete load in the mouse ear biopsy was calculated as log10[(*23S rRNA*/ 10^6^ *Beta-actin*) + 1] and was converted to a z-score. A GLM with quasibinomial errors found a strong positive relationship between ear biopsy spirochete load and lifetime HTT across the 11 *B*. *burgdorferi* strains (slope = 2.357, SE = 0.467, p < 0.001). Strains from eastern and midwestern Canada are shown in blue and red, respectively. Labels on datapoints are strain ID numbers.

The GLMM that modelled the NIP as a function of ear biopsy spirochete load, mouse sex, and infestation is described in **ESM Section 8**. A significant 2-way interaction (p = 0.002) indicates that the slope relating NIP to ear biopsy spirochete load differs between infestations. The slope of the relationship between the NIP and the ear biopsy spirochete load was negative for infestation 1 (slope = -0.790, SE = 0.278, p = 0.004), whereas it was positive for infestation 2 (contrast of slopes = 1.261, SE = 0.407, p = 0.002) and for infestation 3 (contrast of slopes = 1.011, SE = 0.300, p = 0.001). This result indicates that *B*. *burgdorferi* strains that establish higher spirochete loads in the ear tissues have higher host-to-tick transmission to feeding ticks for infestation 2 at day 60 PI and for infestation 3 at day 90 PI. To visualize the relationship, we graphed the mean lifetime NIP for each strain versus its mean ear biopsy spirochete load (**Fig 4**). There was a strong positive relationship between ear biopsy spirochete load and lifetime NIP across the 11 strains of *B*. *burgdorferi* (**Fig 4;** slope = 2.357, SE = 0.467, p < 0.001).

### Correlation matrix of mouse tissue spirochete loads and *B*. *burgdorferi* transmission variables

**Fig 5:**
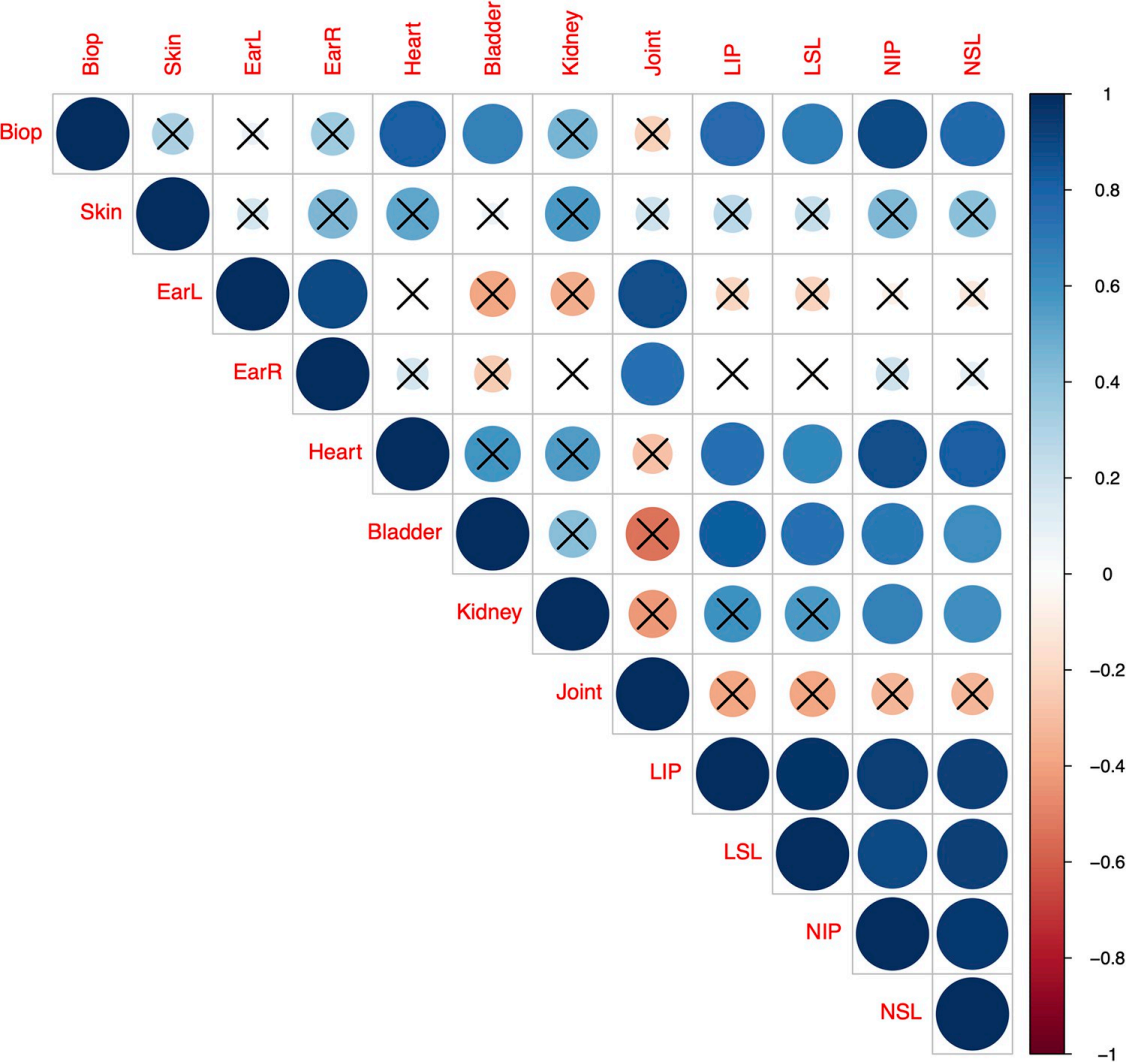
Genetic correlation matrix for 12 variables across the 11 strains of *B*. *burgdorferi*. The 12 variables include spirochete loads in eight mouse tissues: (1) ear biopsy, (2) ventral skin, (3) left ear, (4) right ear, (5) heart, (6) bladder, (7) kidney, (8) right rear tibiotarsal joint, and four estimates of host-to-tick transmission of *B*. *burgdorferi*: (9) larval infection prevalence (LIP), (10) larval spirochete load (LSL), (11) nymphal infection prevalence (NIP), and (12) nymphal spirochete load (NSL). The NIP is equivalent to host-to-tick transmission (HTT). The size and color of the circle indicate the absolute value and direction of the Pearson correlation coefficient, respectively. Non-significant pairwise correlations are indicated with an ‘X’.

To visualize the pairwise correlations between our 8 mouse tissue spirochete load variables and our 4 *B*. *burgdorferi* transmission variables, we calculated the genetic and phenotypic correlation matrices. For the genetic correlation matrix, the four estimates of host-to-tick transmission, LIP, LSL, NIP, and NSL, were all positively correlated with each other (**Fig 5**). Spirochete loads of the ear biopsies, heart, bladder, and kidney were positively correlated with the NIP and the NSL. Spirochete loads of the ear biopsies, heart, and bladder were positively correlated with the LIP and the LSL. The results for the phenotypic correlation matrix were very similar (**see ESM Section 9**).

### Relationship between lifetime NIP and frequencies observed in *I*. *scapularis* ticks in North America across 11 *B*. *burgdorferi* strains

We tested whether there was a relationship between the lifetime NIP and the MLST frequencies observed in *I*. *scapularis* ticks in North America across the 11 *B*. *burgdorferi* strains (**see ESM Sections 10 and 11 for details**). The lifetime NIP was calculated by summing the infected and uninfected nymphs over all infestations and mice for each of the 11 *B*. *burgdorferi* strains. We analyzed the frequencies that included the other MLSTs when the two regions were combined (i.e., MLST frequency 2), and when the two regions were adjusted for sampling effort (i.e., MLST frequency 4). MLST frequency was analyzed using a GLM with quasibinomial errors (to correct for overdispersion) as a function of geographic region (2 levels: northeastern North America and midwestern North America), strain-specific lifetime NIP, and their interaction.

**Fig 6:**
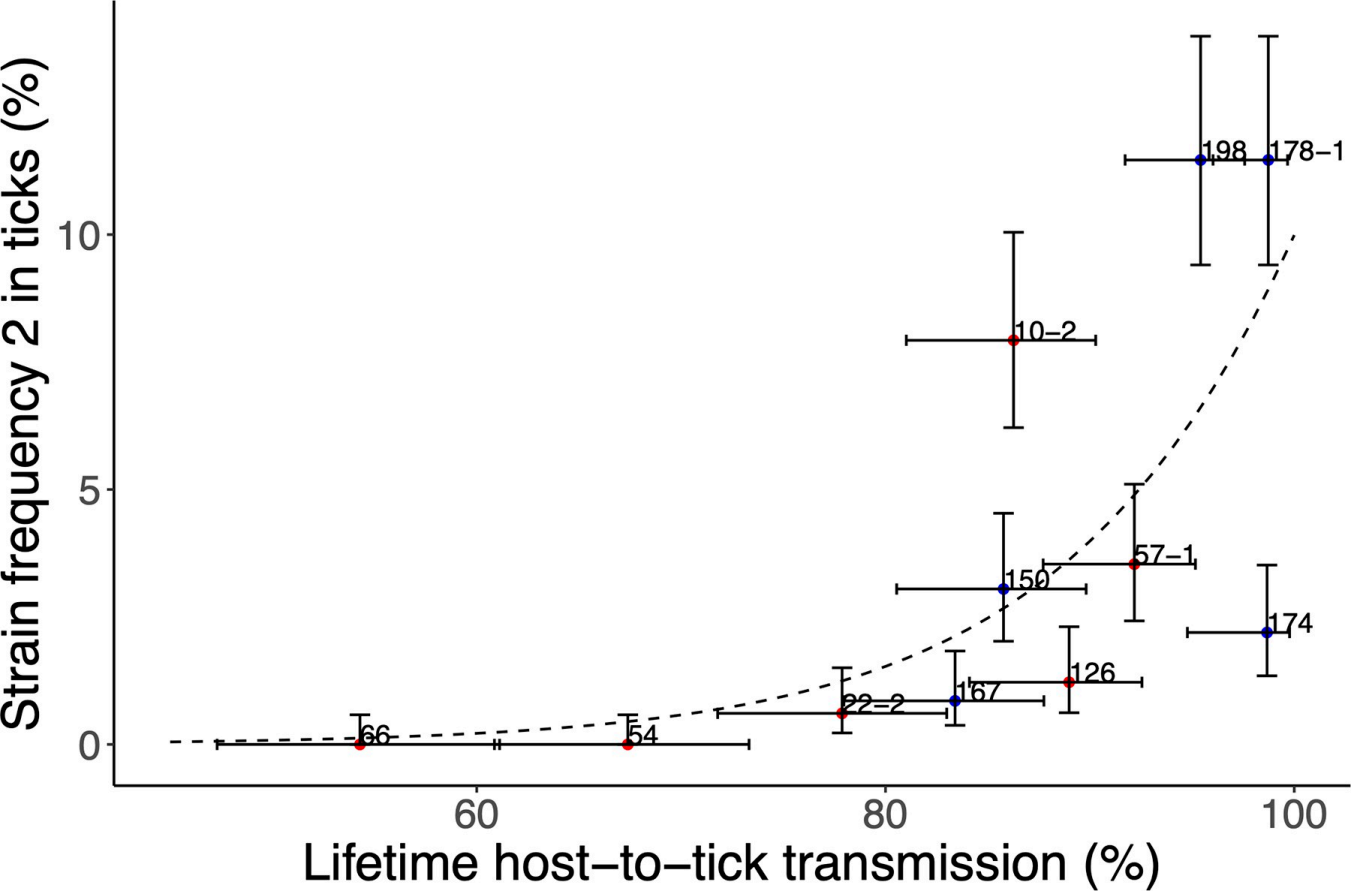
Our laboratory-based estimates of the lifetime host-to-tick transmission for the 11 strains of *B*. *burgdorferi* are positively related to the frequencies of these strains in natural populations of *I*. *scapularis* ticks in North America. The strain-specific frequencies were combined for both the northeastern (blue) and midwestern (red) strains and did not adjust for differential sampling effort between regions. The slope of the logistic regression is positive and significant (slope = 9.685, SE = 3.724, p = 0.029). The error bars represent the 95% confidence intervals.

For MLST frequency 2, geographic region (p = 0.970) and its interaction with lifetime NIP (p = 0.962) were not significant and were therefore removed from the model. Lifetime NIP had a significant effect on MLST frequency 2 (p = 0.001). The slope of the relationship between lifetime NIP and MLST frequency 2 was positive (**Fig 6**; slope = 9.685, SE = 3.724, p = 0.029). Strains with higher lifetime NIP had a higher frequency in *I*. *scapularis* populations in nature. The results were similar for MLST frequency 4 (**see ESM Section 11 for details**). The slope of the relationship between lifetime NIP and MLST frequency 4 was positive (slope = 8.708, SE = 3.942, p = 0.055). After adjusting for differential sampling effort between the two geographic regions, strains with higher lifetime NIP had a higher frequency in *I*. *scapularis* populations in nature.

## Discussion

### Strain-specific estimates of host tissue abundance predict lifetime host-to-tick transmission

Our primary measure of host-to-tick transmission (HTT) for the 11 strains of *B*. *burgdorferi* was the nymphal infection prevalence (NIP), a common metric of HTT of *B*. *burgdorferi* sl genospecies [36,37,56]. We found that our estimates of lifetime NIP (hereafter lifetime HTT) for the 11 strains of *B*. *burgdorferi* were significantly positively correlated with the spirochete load in host tissues, such as the heart, bladder, and ear biopsies (**Figs 3 and 4**). Our results agree with previous studies that found a positive phenotypic correlation between host tissue spirochete load and HTT [13,26,27]. The pairwise correlation matrix found that all four transmission measures, LIP, LSL, NIP, and NSL, were strongly correlated with each other and they were strongly correlated with the spirochete loads in the mouse heart, bladder, and ear biopsies (**Fig 5**). The demonstration of a positive correlation between host tissue spirochete load and HTT across 11 genetically distinct strains of *B*. *burgdorferi* suggests an underlying genetic correlation between these two life history traits, which shapes their evolution. The present study suggests that strains of *B*. *burgdorferi* are under strong selection to maintain high as possible abundance in the tissues of their reservoir hosts to achieve high HTT to feeding *I*. *scapularis* ticks. In summary, this study demonstrates the following causal chain linking spirochete replication inside the host to lifetime HTT, high spirochete load in the host tissues increases the probability that feeding larvae will acquire spirochetes, and these engorged infected larvae have a high probability of moulting into infected nymphs.

Pathogen life history theory typically assumes that the cost of maintaining high pathogen abundance in the host tissues is reduced host survival (*i*.*e*., increased virulence) [1–6]. Thus, higher virulence typically acts as a brake on higher pathogen abundance in the host tissues [1–6]. However, there is little evidence that infection with *B*. *burgdorferi* influences the survival of adult *P*. *leucopus* [38–40], although there is some evidence that infection with *B*. *afzelii* influences the reproduction of bank voles (*Myodes glareolus*) [57]. Infected *P*. *leucopus* mice develop a strong antibody response against *B*. *burgdorferi* that likely helps to control spirochete loads in the mouse tissues [25,58]. Infection with *B*. *burgdorferi* does not induce obvious pathology in the tissues of adult *P*. *leucopus* mice [41,59], although one study found pathology in juvenile *P*. *leucopus* [41]. A review of the reservoir competence of *Peromyscus* mice emphasized that their immune system has evolved to be tolerant of *B*. *burgdorferi* sl and other tick-borne pathogens [40]. It is likely that there is strong selection against virulence of *B*. *burgdorferi* in its rodent reservoir hosts as persistence of *B*. *burgdorferi* transmission demands mobility of the hosts to contact the tick vector which, unlike flying hematophagous insects, has limited mobility and adopts an ambush strategy [60].

### Host tissue type and transmission of *B*. *burgdorferi* to feeding ticks

Many vector-borne pathogens maintain high abundance in the blood to achieve transmission to feeding arthropod vectors [12,61,62]. *Borrelia burgdorferi* has low abundance in the blood of vertebrate hosts [63] and bacteremia is not important for host-to-tick transmission [64]. For *B*. *burgdorferi* sl pathogens, the host skin is believed to be the critical organ for transmission to feeding *Ixodes* ticks, and this has been demonstrated experimentally for *B*. *afzelii* [13,26,27]. It could be argued that the spirochete loads in the ear biopsies would be expected to have a stronger relationship with the NIP compared to the spirochete loads in the tissue samples collected at necropsy. The reason is because the ear biopsies were sampled the day before each of the three larval infestations, and therefore provide a snapshot of the mouse tissue spirochete load immediately prior to each of the three transmission events. For the mice in this study, we had previously shown that the ear biopsy spirochete loads remained constant over time for some strains, whereas they decreased dramatically over time for other strains [42]. As expected, the abundance of *B*. *burgdorferi* in the ear biopsies sampled the day before each of the three larval infestations was a good predictor of the NIP (**Fig 4**). This result agrees with previous studies on *B*. *afzelii* that have found positive relationships between the spirochete load in ear biopsies and the infection prevalence in feeding *Ixodes* ticks [13,27]. One reason why ear biopsies may be less reliable than necropsy tissues is because they are a much smaller sample of skin and because repeated biopsy sampling damages the ear tissue, which probably has a negative impact on the resident spirochete population.

Given that the skin is believed to be the critical organ for host-to-tick transmission [13,26,27], it could be considered surprising that the spirochete loads in the internal organs (e.g., heart and bladder) at necropsy were better predictors of the NIP compared to the spirochete loads in the skin-related tissues (e.g., ventral skin, left ear, right ear). However, in infected hosts there is possibly a complex dynamic of movement of spirochaetes amongst internal organs and the skin from where they are transmitted, involving chemotaxis of spirochaetes from internal organs to the skin, and from skin to tick, associated with responses to tick saliva [65,66], with depletion of spirochaete loads in skin where ticks are feeding (particularly the ears from where mice have difficulty grooming ticks off). The hypothesis that the larval infestation decreased the spirochete load in the skin explains the following three results. First, the infection prevalence and the spirochete loads of the skin-related tissues (ventral skin, left ear, right ear) at necropsy (*i*.*e*., shortly after the third larval infestation) were much lower compared to the internal organs (heart, bladder). Second, infection prevalence was 91.7% in the third biopsy of the right ear on day 89 PI (prior to tick feeding) and then dropped to 14.3% in the right ear sample collected at necropsy on day 97 PI, which was 7 days after the third larval tick infestation. Third, the mean spirochete loads of the three biopsies of the right ears were correlated with the spirochete loads in the heart and bladder collected at necropsy, but not with the spirochete loads in any of the skin samples, including the right ear, collected at necropsy (**Fig 5**). In summary, we believe that the larval infestation on day 90 PI transiently reduced the spirochete loads in the skin-related tissues, but not in the internal organs like the heart and bladder. As a result, the heart and bladder were better predictors of the lifetime NIP compared to the skin-related tissues. Future studies should investigate whether larval infestation influences the spirochete load in different host tissues.

### Strain-specific estimates of lifetime HTT are correlated with strain-specific frequencies in nature

Our strain-specific estimates of lifetime HTT were significantly positively correlated with the frequencies of these strains in field and surveillance studies (**Fig 6**). The strains that have higher HTT in an experimental setting were also the ones that are more commonly found in *I*. *scapularis* ticks in nature in Canada and the USA. We recently showed that the mean spirochete load for the subset of infected mouse tissues was significantly positively correlated with strain frequency [42]. In that study, we calculated the mean spirochete loads without standardizing each mouse tissue to z-scores and this mean was therefore dominated by the spirochete loads in the heart and bladder (i.e., because these tissues have high infection prevalence and high spirochete loads) [42]. The positive relationship between lifetime HTT and strain-specific frequency in the present study agrees with a previous study on *B*. *afzelii* that found that laboratory-based estimates of the R_0_ values of six *ospC* strains were related to their frequencies at a local field site in Neuchatel, Switzerland [18]. The present study is more general because the strain-specific frequencies were obtained from *I*. *scapularis* tick populations that were distributed across North America [46,53].

The estimation of unbiased MLST frequencies in nature using the published literature is complicated. The ticks in the study by Ogden et al [46] were collected from humans and companion animals as part of national passive surveillance system in Canada. Passive surveillance may result in skewed estimates of pathogen strain structure because ticks are mostly collected from humans and domestic pets, rather than from the environment or from natural hosts, and because passive surveillance also collects adventitious ticks that may have been dispersed long distances from their origin by migratory birds [67]. The ticks in the study by Travinsky et al [53] had been collected using a standardized sampling protocol at 40 different field sites in the northeastern, mid-Atlantic, and north central (midwestern) USA [53,68,69]. Both studies indicated whether the MLSTs were from the northeastern or the midwestern (north central) USA, but some MLSTs occurred in multiple geographic regions. The study by Ogden et al [46] had 15.6 times more MLSTs from the Northeast compared to the Midwest. For this reason, we calculated the MLST frequencies for the two geographic regions combined (frequency 2) and separately (frequency 4). Other complications included strains that had no matching MLST in the published data sets (Strains Bb16-66 and Bb16-54) and strains that matched with respect to their MLST but not their *ospC* type (Bb16-10-2 and Bb16-57-1). Despite these challenges, we found a positive relationship between our laboratory estimates of lifetime HTT and frequency in *I*. *scapularis* ticks in North America across 11 strains of *B*. *burgdorferi*. This result suggests that strains that have the highest fitness in *M*. *musculus* mice in the laboratory (even though *M*. *musculus* is not a natural host of *B*. *burgdorferi*) are those that are most common in nature, and that laboratory studies can enhance our understanding of pathogen strain structure in nature.

Previous studies have shown that strains of *B*. *burgdorferi* can be transmitted by many different vertebrate hosts including mice, chipmunks, squirrels, shrews, and other species [30, 70–73]. However, studies have identified considerable variation among vertebrate host species in their contribution to the epidemiology of *B*. *burgdorferi* [56,73,74], which depends on a number of factors including (1) host density, (2) numbers of larval ticks that are fed by the host, (3) probability that the host is infected with *B*. *burgdorferi*, and (4) probability that ticks acquire *B*. *burgdorferi* after feeding on an infected host (often referred to as host reservoir competence or host infectivity) [56,73,74]. Numerous studies have suggested that rodents, such as *P*. *leucopus* and the eastern chipmunk (*Tamias striatus*), are critical for determining the density of infected nymphs (DIN) and the nymphal infection prevalence (NIP) in an area [75–77]. Rodent-targeted interventions that are highly effective at reducing the DIN and/or NIP provide further evidence that *P*. *leucopus* is a keystone species for the epidemiology of *B*. *burgdorferi*. Thus, *B*. *burgdorferi* strains that are specialized for host species that are abundant and have high reservoir competence, such as *P*. *leucopus*, will have high frequency in nature. Strains that are specialized for host species that are rare and/or have low reservoir competence will have low frequency in nature.

The multiple niche polymorphism (MNP) hypothesis suggests that different *B*. *burgdorferi* strains are adapted to different host species [30,70–72,78]. Evidence for the MNP hypothesis comes from field studies showing that different *B*. *burgdorferi* strains are associated with different host species [30,70–72]. Some studies on *B*. *burgdorferi* in North America have found host-strain associations [30,71], whereas others have not [70,72]. One criticism of all these studies [30,70–72] is that they do not control for spatiotemporal variation in exposure. Another criticism of these studies is their small sample sizes (n < 100 animals). The most comprehensive study on host-strain associations was done on 7 *B*. *afzelii ospC* strains in 3 different host species (bank vole, yellow-necked mouse, common shrew) with a sample size of ~2800 animals [19]. This study found no evidence of host-strain associations; common strains had high frequencies in all three host species and vice versa for rare strains [19]. The bank vole (*M*. *glareolus*) and yellow-necked mouse (*Apodemus flavicollis*) belong to the Cricetidae and the Muridae, respectively, which shared a common ancestor 35 million years ago (mya). Rodents and shrews belong to Euarchontoglires and Laurasiatheria, respectively, which shared a common ancestor ~100 mya [79]. The observation that the frequencies of these *B*. *afzelii ospC* strains is highly correlated across these three divergent host species suggests that the role of host-strain interactions is negligible for *B*. *afzelii*. With respect to our study, *P*. *leucopus* and *M*. *musculus* belong to the Cricetidae and the Muridae, respectively, which shared a common ancestor 35 mya [40]. An experimental infection study that used both these host species and two strains of *B*. *burgdorferi* found a significant host-strain interaction [34]. However, the pattern of HTT was generally the same in the two host species with no changes in the ranking of the strains between hosts [34]. We suggest that the strains that have high HTT in our C3H/HeJ lab mice are also the strains that have high HTT in common rodent reservoirs such as *P*. *leucopus*. It is possible that strains that have short-lived transmissible infections and/or relatively low HTT may be better adapted to species other than *P*. *leucopus* mice, such as voles, chipmunks, shrews, or birds. Future experimental infection studies should investigate whether fitness of different *B*. *burgdorferi* strains is correlated across different vertebrate hosts.

### Mixed strain infections in the host and interactions among *B*. *burgdorferi* strains

In nature, vertebrate reservoir hosts are often infected with multiple strains of the same *B*. *burgdorferi* sl pathogen. Field studies on *B*. *burgdorferi* in North America [30,80] and on *B*. *afzelii* in Europe [81–83] have shown that mixed strain infections are the norm in endemic areas. Mixed strain infections in the vertebrate host result in competitive interactions that reduce host-to-tick transmission of strains to feeding *Ixodes* ticks and this phenomenon has been shown for *B*. *burgdorferi* in *P*. *leucopus* [31,36] and for *B*. *afzelii* in *M*. *musculus* [26,32,33]. The mechanism underlying competition between strains of *B*. *burgdorferi* sl is not known, but strain abundance in host tissues may be important. An experimental infection study of BALB/c mice with two strains of *B*. *afzelii* found that the strain that established the higher spirochete load in the tissues of co-infected mice had higher HTT to feeding *I*. *ricinus* ticks [26]. Thus, high abundance in the host tissues, whether alone or in mixed infections, enhances strain transmission to feeding ticks. Studies on the rodent malaria parasite, *Plasmodium chabaudi*, have shown that strains that establish the highest abundance in the blood when alone in the rodent host are also the strains that are the most competitive in mixed strain infections [61]. Whether this phenomenon is true for *B*. *burgdorferi* remains to be tested.

### Male mice have higher transmission of *B*. *burgdorferi* to *I*. *scapularis* ticks

An interesting result was that the lifetime HTT was higher for male mice compared to female mice and this was true for 9 of 11 strains of *B*. *burgdorferi* (**Fig 2**). We had recently shown that male mice have significantly higher spirochete loads in their tissues, especially the skin, compared to female mice [42,84]. For the C3H/HeJ mice in the present study, the spirochete load in the ventral skin of the male mice was 15.3 times higher compared to female mice [42]. In another study on C3H/HeJ mice where *B*. *burgdorferi* was re-activated by the application of immunosuppressive corticosteroids, the spirochete load in the skin (averaged over four different sites) of the male mice was 72.6 times higher compared to female mice [84]. Testosterone-based immunosuppression is a general explanation why males often have higher pathogen abundance in their tissues than females [85–87]. The present study suggests that male mice have higher HTT because they have higher spirochete loads in their tissues compared to female mice. Field studies on *P*. *leucopus* in the USA and on bank voles (*M*. *glareolus*) in Sweden have shown that male rodents are more likely to be infected with *B*. *burgdorferi* sl than their female counterparts [38,88–90]. One reason for this sex bias in infection prevalence in the field is that male rodents have larger home ranges than female rodents and are more likely to encounter *B*. *burgdorferi*-infected nymphs [38,91–94]. For the same reasons, male rodents are more likely to encounter and feed a greater number of larval ticks [38,91–94]. Assuming similar survival rates between the sexes, our study suggests that male rodents contribute more to the prevalence of infected ticks in nature and hence to the risk of Lyme disease compared to their female counterparts. Future studies should investigate whether similar sex-specific differences in tissue spirochete load and HTT occur in wild rodent reservoir hosts.

### HTT decreases over time for most *B*. *burgdorferi* strains

Interestingly, 10 of 11 *B*. *burgdorferi* strains had HTT greater than 90% for the first larval infestation (**Fig 1**), suggesting that there is little variation in fitness among these strains early in the infection. Pathogen life history theory predicts that early transmission is more important for pathogen fitness than late transmission, and this is especially true if the hosts are small, short-lived prey species. Field studies suggest that most *P*. *leucopus* mice do not survive to see a second Lyme disease transmission season [38,40,58,64,95]. Capture-mark-recapture studies in the field suggest that monthly survival rates of adult *P*. *leucopus* can be as low as 50% [38]. Assuming a host mortality rate of 50%, a strain that reaches peak HTT at 30 days PI will be twice as fit compared to a strain that reaches peak HTT at 60 days PI. Thus, all strains are under strong selection to have high HTT over the early phase of the infection (i.e., the first 30 days). Nevertheless, the observation that all strains established persistent infection in the mice suggests that this trait must have adaptive value. The fact that *B*. *burgdorferi* has evolved complicated antigenic variation mechanisms that allow it to evade the host antibody response [96] provides further evidence that persistent infection in the host must provide some fitness benefits.

Lifetime HTT remained above 90% over the course of the infection for 3 strains from the northeast (Bb16-174, Bb16-178-1, and Bb16-198 in **Fig 1**). For the other 8 strains, HTT decreased over the three successive infestations, which agrees with other studies [34,35,97,98], and the magnitude of the decrease determined variation in lifetime HTT (**Fig 1**). Interestingly, the 3 strains with the most dramatic decline in HTT over time (Bb16-22-2, Bb16-54, and Bb16-66 in Fig 1) are from the Midwest. Differences in climate between the Northeast and Midwest cause differences in the seasonal phenology of immature *I*. *scapularis* ticks, which might explain these differences in lifetime HTT. In North America, nymphs become active and infect the reservoir hosts in the spring and early summer; the larvae subsequently acquire the spirochetes from these infected hosts in the late summer and early fall [23,64]. In the Northeast, the time interval between the larval peak and the nymphal peak is longer and their questing activity is therefore less synchronous compared to the Midwest [99]. Thus, strains in the Northeast must persist for longer in their rodent reservoir hosts compared to strains in the Midwest. Taken together, these observations suggest that the more synchronous questing behaviour of immature *I*. *scapularis* ticks in the Midwest relaxes selection on midwestern strains to have persistently high HTT over the course of the infection. There were too few strains studied here to explore this, but indeed the three strains that had constantly high HTT at all infestations were from the northeast (Bb16-174, Bb16-178-1 and Bb16-198 from Nova Scotia), while the three strains with the lowest HTT at the second and third infestations were from the Midwest (Bb16-22-2, Bb16-54 and Bb16-66 from Manitoba).

## Conclusions

We tested the variation in host-to-tick transmission among strains of *B*. *burgdorferi* using a laboratory model host. We found that these estimates of HTT were positively correlated with measures of host tissue spirochete load, and with the strain frequencies in nature. From this we suggest a simple mechanistic explanation, where strains that establish a higher spirochete load in the tissues of the vertebrate host are more likely to be acquired by feeding larvae, which in turn results in more infected questing nymphs. The strains with higher spirochete loads in the host tissues and higher lifetime host-to-tick transmission are thus more common in nature.

## Supporting information

S1 Information Electronic supplementary material (ESM). (PDF)
