# Supplementary material for: Variation among strains of Borrelia burgdorferi in host tissue abundance and lifetime transmission determine the population strain structure in nature
Source: PLoS Pathog. 2023 Aug 22;19(8):e1011572. doi: 10.1371/journal.ppat.1011572 (PMC10473547; doi:10.1371/journal.ppat.1011572)
Supplement: S1 Information — (PDF) [file ppat.1011572.s001.pdf]

Supporting Information

Title: Variation among strains of *Borrelia burgdorferi* in host tissue abundance and lifetime transmission determine the population strain structure in nature

Authors: Christopher B. Zinck, Prasobh Raveendram Thampy, Eva Uhlemann, Hesham Adam, Jenny Wachter, Danae Suchan, Andrew D. S. Cameron, Ryan O. M. Rego, Dustin Brisson, Catherine Bouchard, Nicholas H. Ogden, and Maarten J. Voordouw

Table of Contents

Section 1 – Additional Methods 2

Section 2 – Synthetic standards used in qPCR 4

Section 3 – Infection prevalence and spirochete load of mouse tissues 6

Section 4 – Analyses of the larval infection prevalence (LIP) 9

Section 5 – Analyses of the larval spirochete load (LSL) 12

Section 6 – Analyses of the nymphal infection prevalence (NIP) 17

Section 7 – Analyses of the nymphal spirochete load (NSL) 22

Section 8 – Relationship between the mouse tissue spirochete loads and NIP 28

Section 9 – Correlation matrix of mouse tissue spirochete loads and *B. burgdorferi* transmission variables 33

Section 10 – Estimation of MLST frequencies in *I. scapularis* ticks in North America. 36

Section 11 – Relationship between laboratory estimates of lifetime NIP and frequencies observed in *I. scapularis* ticks across 11 *B. burgdorferi* strains. 41

Section 12 - References 47

Section 1 – Additional Methods

Strains of *Borrelia burgdorferi*: The strains of *Borrelia burgdorferi* were obtained from the National Microbiology Laboratory (NML) in Winnipeg, which is part of the Public Health Agency of Canada (PHAC). These strains were originally isolated from questing *I. scapularis* ticks that were collected at various field sites across Canada [1]. To obtain single-strain colonies, tick homogenates were cultured in semi-solid agar (passage 1). For each tick, two colonies were cut out of the agar and grown in vials containing liquid BSK-H (passage 2). The methods for sampling the strains and creating the single-strain colonies are described in [1]. Once we had selected the set of *B. burgdorferi* strains that we wanted to work with, the strains were grown up at the NML (passage 3) before being shipped to the University of Saskatchewan (USask). At USask, we grew each strain in BSK-H medium (passage 4) before needle inoculation into C3H/HeJ mice (see below). Thus, each isolate was passaged 4 times from the original isolation from field-collected *I. scapularis* ticks to needle-inoculation into mice.

The single strain colonies were subsequently whole genome sequenced by PHAC and the strains were classified according to their multi-locus sequence type (MLST) and *ospC* type [1]. MLST is determined by the allelic identity at eight housekeeping loci: *clpA*, *clpX*, *nifS*, *pepX*, *pyrG*, *recG*, *rplB*, and *uvrA* [2, 3]. The identity of alleles at these eight housekeeping loci and the resultant MLST can be determined using PubMLST [4]. The MLST and *ospC* types for each of the 11 *B. burgdorferi* strains were published in [1].

Corrections to genetic identity of *B. burgdorferi* strains: The NML in Winnipeg sent us a manifest that indicated the MLST and the *ospC* groups for the 11 *B. burgdorferi* strains, which we reported in [5]. In comparing this manifest to the supplementary material of [1], we realized that the manifest contained some errors with respect to the *ospC* types for two strains, Bb16-167 and Bb16-174. In the present study, we report that the *ospC* types for strains Bb16-167 and Bb16-174 are M and T (Table 1), respectively, and not C and D, as was reported in [5].

We checked the identity of the *B. burgdorferi* strains by downloading the original sequence data from [1] and found the same MLSTs as reported by [1]. We compared the original *ospC* sequence data from [1] to the reference sequences listed in [6]. For strain Bb16-57-1, we discovered that the *ospC* gene that had been annotated as type Y in [1], is annotated as type A3 in [6]. These two *ospC* types are easily confused because the reference sequences are >98% similar (Y: HM047875.1; A3: EF592541.1), which is much less than the > 8% criterion for distinguishing among different *ospC* types [6, 7]. For simplicity, in the present study, we report that the *ospC* type for strain Bb16-57-1 is A3 (Table 1) and not Y, as was reported in [1] and [5].

In-house Sanger sequencing of MLST and *ospC*: We tested the MLST identity for 3 of our 11 strains, Bb16-10-2, Bb16-167, and Bb16-174. We used DNA from the *B. burgdorferi* strain cultures that were inoculated into the mice to create the *I. scapularis* nymphs used to experimentally infect the mice in the present study. We PCR-amplified the eight housekeeping genes using an established protocol [2, 3] and sent the amplicons for Sanger sequencing. This in-house sequencing confirmed that the MLSTs of the these 3 cultures were identical to the MLSTs identified by [1].

To further confirm the *ospC* type of our strains, we used a nested PCR to amplify the *ospC* gene from various sources including cultures of *B. burgdorferi*, tissues of experimentally infected mice, engorged larval ticks, and engorged nymphal ticks. The nested PCR protocol for the *ospC* gene was described in [8], and the purified amplicons were sent for Sanger sequencing. We obtained high quality Sanger sequences for 43 samples including 10 cultures of *B. burgdorferi*, 3 mouse tissues, 13 engorged larval ticks, and 17 engorged nymphal ticks. Of the 43

ospC sequences, 39 were identical to the *ospC* type in [1]. For strain Bb16-10-2, there was a mismatch in *ospC* type between [1] and our in-house Sanger sequencing of the *ospC* amplicons for 4 infected ticks. The whole genome sequence in [1] was annotated as *ospC* type H, but the forward and reverse Sanger sequences of our 4 infected ticks clustered with *ospC* type C3.

In an earlier version of this manuscript, we had reported a mismatch in *ospC* type between [1] and our in-house sequencing for strains Bb16-10-2 and Bb16-57-1. A reviewer suggested that strains Bb16-10-2 and Bb16-57-1 might be mixed strain infections. As mentioned above, Bb16-57-1 had an unambiguous *ospC* sequence that could be called A3 or Y, so this is not a mixture of strains. Nevertheless, to address the concern of possible mixed strain infections, we amplified the *ospC* gene for each strain from 24 infected mouse tissues (8 mice x 3 tissues per mouse) and 24 infected unfed nymphs (8 mice x 3 unfed nymphs per mouse; nymphs had taken their larval blood meal from the mice) and sent the amplicons for Sanger sequencing. We obtained 141 high quality Sanger sequences of the *ospC* gene (69 forward and 72 reverse; 51 from mouse tissues and 90 from unfed nymphs), of which 87 and 54 were from strains Bb16-10-2 and Bb16-57-1, respectively. The *ospC* sequences were evenly distributed over the 16 mice with a median of 10 sequences per mouse (range = 5 to 10). The *ospC* sequences were matched to the reference sequences A3_EF592541 and C3_EF592543, and the mean % identical sites was 99.9% (range = 98.8 – 100.0%). All 87 *ospC* sequences from the mice infected with strain Bb16-10-2 clustered with *ospC* type C3 with a mean % similarity of 99.93% (range = 98.8 – 100.0%). All 54 *ospC* sequences from the mice infected with strain Bb16-57-1 clustered with *ospC* type A3 with a mean % similarity of 99.95% (range = 99.2 – 100.0%). In summary, we are very confident that strains Bb16-10-2 and Bb16-57-1 are not mixed infections and that the *ospC* types of the strains that we used in our study are C3 and A3, respectively and not H and Y, as originally reported in Zinck et al (2022).

Switch of strains Bb16-178-2 and Bb16-178-1: At the start of our study, we had requested strain Bb16-178-2, which according to [1] has MLST 12, *ospC* type O, and ID 2421 in the PubMLST database. The in-house genetic testing of the strain that we had received found MLST 3 and *ospC* type K. According to [1], strain Bb16-178-1 has MLST 3 and *ospC* type K. Strain Bb16-178-1 and strain Bb16-178-2 were isolated from the same adult male *I. scapularis* tick collected from Lunenberg, Nova Scotia. This suggests that we received strain Bb16-178-1 instead of Bb16-178-2. This error was detected after we started our experimental infection and was pointed out in an earlier article published on this study [5]. It is for this reason, that our study contains two strains, Bb16-178-1 and Bb16-198, that are in fact different, but are both MLST 3 and *ospC* type K.

Larval infestation of mice: Specific pathogen-free *Ixodes scapularis* larval ticks were purchased from the National Tick Research and Education Resource at Oklahoma State University. At days 30, 60, and 90 post-infection (PI), mice were infested with 50-100 *I. scapularis* larvae. All mice were anaesthetized for ~20 min with ketamine and xylazine for each infestation, where larvae were brushed onto their fur. During each infestation, mice were housed in their mouse cage with the plastic top removed and placed on risers within a rat cage containing a shallow moat of water. As a precaution, a 1-inch-tall strip of petroleum jelly was put around the inner wall of each rat cage. Engorged larvae climbed out of the mouse cage and fell into the water moat of the rat cage where they were easily recovered. At 5 days post-infestation, most larvae were in the water moat, and no larvae were found in the bedding by 7 days post-infestation. At 7-8 days post-infestation, the mice were housed under normal conditions. For each mouse and infestation, 5 engorged larvae were frozen immediately at -80 ºC following

recovery (this was not done for the first infestation in block 1; it was done for infestations 2 and 3 in block 1 and for infestations 1, 2, and 3 in block 2). The remaining engorged larvae were placed into 50 mL Falcon tubes where they were allowed to moult into nymphs. These tubes had perforated lids and a double layer of nylon stocking to allow air exchange. The centrifuge tubes were placed in sealed clear plastic containers that had a layer of standing ultrapure water (Milli-Q) to ensure a high relative humidity (~100%). Four weeks after the engorged larvae moulted into nymphs, a sample of 10 nymphs per mouse per infestation were frozen at -80 ºC for later DNA extraction.

DNA extraction of whole *Ixodes scapularis* ticks: For each mouse, there were 45 ticks (3 infestations * (5 larvae + 10 nymphs) per infestation) for a total of 5,040 tick DNA extractions (112 mice x 45 ticks per mouse = 5,040 ticks). Ticks were homogenized using bead beating with the Qiagen TissueLyser II. First, ticks were placed into 5 µL of nuclease free water at the bottom of a 2.0 mL MP BIO disruption tube. These were then frozen for 1-2 hrs at -80 ºC before adding a 1.6 mm stainless steel bead, and refreezing inverted at -80 ºC for a minimum of 24 hours. This allows the tick to freeze in place at the center of the tube bottom but keeps the bead from freezing to the tick. The ticks were then disrupted with the TissueLyser II with two runs at 30 Hz for 45 seconds, flipping the two racks of tubes in between runs. DNA was extracted from the homogenized ticks using the Qiagen DNEasy 96-well plate extraction kit and following the manufacturer's instructions. The disrupted ticks were extracted following the kit guidelines with some modifications. The plate extraction was done in batches of 192 samples per run. Each run included 8 no sample controls (i.e., with no DNA substrate added), and experimental controls (ticks from uninfected control mice). The proteinase K digestion step was done overnight in the MP BIO tubes without removing the disruption bead in a shaking incubator (56 ºC, 400 rpm). Samples were then moved into the Qiagen kit plates for the remainder of the extraction. All extractions were eluted into a final volume of 65 µL and stored at -80 ºC prior to use in qPCR.

Statistical software: We used R version 4.0.4 with RStudio version 1.4.1103 for all statistical analyses [9, 10]. We used the *lmer()* function in the lme4 package to run the LMMs, and *glmer()* to run the GLMMs [11]. The *Anova()* function in the car package was used to run the Wald tests of statistical significance [12]. Post-hoc analysis and calculation of estimated marginal means were done using *emmeans(), pairs(), pwpm()* functions in the emmeans package [13] and *ggemmeans()* and *ggpredict()* in the ggeffects package [14]. Correlations were calculated using the *cor.test()* function in the base package. Correlation matrices and correlation plots were calculated using the *rcorr()* function in the Hmisc package [15].

Section 2 – Synthetic standards used in qPCR

Synthetic *23S rRNA* gene standard: The presence and abundance of *B. burgdorferi* in the *I. scapularis* ticks was estimated using real-time PCR that targeted the *23S rRNA* gene of *B. burgdorferi* and was previously described [16]. To translate the quantification cycle (Cq) values produced by the *23S rRNA* qPCR to estimates of the abundance of *B. burgdorferi* requires a standard curve. We created this standard curve by repeatedly performing the *23S rRNA* qPCR on a dilution series of a synthetic *23S rRNA* gene of known concentrations. The synthetic standard was purchased as a gblock from IDTDNA. The standard was based on the *B. burgdorferi* B31 reference sequence (Genbank Accession number: AE000783.1) to encompass the region targeted by the *23S rRNA* primers (Table S1). A total sequence length of 181 nucleotides (nt) was used to ensure that the forward and reverse primer sites were not at the ends of the gblock where degradation could impact binding. To be able to check for contamination, the *23S rRNA* gene

sequence was modified from the reference genome to allow for two methods of differentiation between the *23S rRNA* gene of *B. burgdorferi* and the synthetic standard. A total of 30 nt were inserted in the synthetic fragment between the forward and reverse priming sites to allow for product discrimination on an agarose gel; the amplicon lengths of the synthetic standard (105 nt) and the *23S rRNA* gene of *B. burgdorferi* strain B31 (75 nt) differ by 30 nucleotides. A *BamHI* restriction enzyme cut site was included in the 30-nucleotide insert so that a restriction digest would cleave the synthetic standard, but not a copy of the real *23S rRNA* gene. The gblock was initially suspended in Buffer AE (Qiagen DNEasy kit) with 0.1 mg/mL tRNA (Sigma-Aldrich: 10109517001). This was done so that the standard was in the same suspension buffer as all the experimental samples. The tRNA was added to reduce the impact of DNA adsorption to the walls of the qPCR tubes in dilutions with a low concentration of gblock *23S rRNA*.

Table S1. The sequences used in creating the *23S rRNA* synthetic standard as well as the primer and probe sequences for the *23S rRNA* qPCR. All sequences are reported in the 5'-3' direction. The *23S rRNA* forward primer and the *23S rRNA* reverse primer are highlighted in yellow and green, respectively. The 30-nucleotide insert sequence and the *BamHI* cut site are shown in red and purple, respectively.

Identity	Sequence (5'-3')
23S rRNA forward primer	CGAGTCTTAAAAGGGCGATTTAGT
23S rRNA reverse primer	GCTTCAGCCTGGCCATAAATAG
23S rRNA probe	AGATGTGGTAGACCCGAAGCCGAGTG
B. burgdorferi B31 original sequence	TTATCATGTCTAGCAAGATTAAAGCATAGAAGTGCTGGAGTC GAAGCGAAAGCGAGTCTTAAAAGGGCGATTTAGTTAGATGTG GTAGACCCGAAGCCGAGTGATCTATTTATGGCCAGGCTGAAG CTTGGGTAAAACCAAGTGGAGGGCC
gBlock synthetic standard	TTATCATGTCTAGCAAGATTAAAGCATAGAAGTGCTGGAGT CGAAGCGAAAGCGAGTCTTAAAAGGGCGATTTAGTGCGGA TCCTATCATGTAGATGTGGTAGACCCGAAGCCGAGTGAGCG CTGATTGCATAATCTATTTATGGCCAGGCTGAAGCTTGGGTA AAACCAAGTGGAGGGCC

Synthetic tick *calreticulin* gene standard: The abundance of the tick genome in the tick DNA extractions was estimated using a real-time PCR that targeted the *calreticulin* gene of *I. scapularis*. A synthetic gblock standard was created for the *calreticulin* gene of *I. scapularis* ticks following the same rationale as above. This standard was based on the reference sequence (Genbank Accession number: AY690335.1) to encompass the region targeted by the *calreticulin* primers (Table S2). The total sequence length of the synthetic tick *calreticulin* gene is 177 nt. Like the *23S rRNA* standard, the internal sequence of the *calreticulin* standard was modified by adding a 33-nucleotide insert that contained a *BamHI* cut site. This 33-nt insert will allow us to

differentiate the standard from a real copy of the *calreticulin* gene. The *calreticulin* standard was suspended in the same manner as the *23S rRNA* standard.

Table S2. The sequences used in creating the synthetic tick *calreticulin* standard as well as the primer and probe sequences for the tick *calreticulin* qPCR. All sequences are reported in the 5'-3' direction. The *calreticulin* forward primer and the *calreticulin* reverse primer are highlighted in yellow and green, respectively. The 33-nucleotide insert sequence and the *BamHI* cut site are shown in red and purple, respectively.

Identity	Sequence (5'-3')
Calreticulin forward primer	AAGCGAGCAGGGAACTTTCA
Calreticulin reverse primer	GAACTTGGTGGACAGTCCGT
Calreticulin probe	CACTGCCGCATCCTCTTCCTCCC
I. scapularis Calreticulin original sequence	CTCCACGAAGAAGGGAAGCGAGCAGGGAACTTTCAAGCTGTCCGCCG GCAAGTTCCACGGCGACCCGGAGAAGGACCTCGGCATCCAGACCTCT GAAGATGCCCGCTTTTACGGACTGTCCACCAAGTTCGAGCCGTTCTCG AAC
gBlock synthetic standard	CTCCACGAAGAAGGGAAGCGAGCAGGGAACTTTCAAGCTGTTCCA GGATCCTTCACCCGCCGGCAAGTTCCACGGCGACCCGGAGAAGGA CCTCGGCATCCAGACCTCTGAAGATGCCCGCCTTCAACACCCCAGC CATTTTACGGACTGTCCACCAAGTTCGAGCCGTTCTCGAAC

Section 3 – Infection prevalence and spirochete load of mouse tissues

Infection prevalence in the mouse tissues: The tissue infection prevalence and the tissue spirochete loads of the 84 infected C3H/HeJ mice in this study have been published elsewhere [5]. To facilitate interpretation, we briefly present here the infection prevalence and the spirochete loads of the 3 ear biopsies and the 7 necropsies. The ear biopsies were sampled from the right ear on days 29, 59, and 89 post-infection (PI), whereas the 7 necropsies were sampled on day 97 PI. The 7 necropsies were the ventral skin, left ear, right ear, heart, bladder, joint, and kidney. The infection status and spirochete load of each mouse tissue were determined using qPCR targeting the *23S rRNA* gene. The spirochete loads were standardized relative to a million mouse *Beta-actin* gene copies. These standardized spirochete loads were transformed as follows: log10[(*23S rRNA* copies/10^6 *Beta-actin* copies) + 1]. qPCR is an exponential process and log transformation normalizes the residuals and gives better estimates of the mean. One problem with log transformation is that it cannot handle uninfected samples that have zero *23S rRNA* copies. For this reason, we used the log[X + 1] transformation, where all uninfected values

have a value of zero (i.e., log[0 + 1] = log[1] = 0). For each mouse tissue, we calculated the percentage of infected tissues and the mean spirochete load with a 95% confidence interval on the log10 scale and the original scale. In Zinck et al (2022), the mean spirochete loads were calculated over the subset of infected tissues (i.e., uninfected tissues were excluded). For this reason, the mean tissue spirochete loads in the present study are different from Zinck et al (2022).

The infection prevalence in the biopsies from the right ear on days 29, 59, and 89 PI were 98.8%, 100.0%, and 91.7%, respectively (**Table S3**). For the necropsies on day 97 PI, the infection prevalence varied among tissue types. Skin-related tissues, such as the ventral skin, left ear, and right ear had low infection prevalence with 52.4%, 38.1%, and 14.3%, respectively. In contrast, the heart, bladder, and joint had high infection prevalence with 92.9%, 97.6%, and 71.4%, respectively. An unexpected result is that the infection prevalence in the right ear changed from 91.7% in the biopsy on day 89 PI to 14.3% in the necropsy on day 97 PI. In other words, over the course of 8 days, the infection prevalence in the right ear decreased 6.4-fold.

Spirochete load in the mouse tissues: The mean spirochete loads for the mouse tissues are shown in **Table S3**. The mean spirochete load was expressed as log10[(*23S rRNA* copies/10^6 *Beta-actin* copies) + 1] for each mouse tissue (Mean1 in **Table S3**). The advantage of the log[X + 1] transformation is that it allows uninfected samples to be defined on the log scale; these samples have a value of 0 (i.e., log[X + 1] = log[0 + 1] = log[1] = 0). Thus, for each mouse tissue, the mean is based on all 84 mice and includes both infected and uninfected tissues. The mean log[X + 1] values were back-transformed to indicate the number of *23S rRNA* copies/10^6 *Beta-actin* copies (Mean 2 in **Table S3**).

For the biopsies of the right ear, the mean spirochete load decreased over the course of infection. The mean spirochete load of biopsy 1 on day 29 PI (2878.5 *23S rRNA* copies/10^6 *Beta-actin* copies) was 3.5x higher compared to the mean spirochete load of biopsy 3 on day 89 PI (812 *23S rRNA* copies/10^6 *Beta-actin* copies). For the necropsies on day 97 PI, there were dramatic differences in mean spirochete load. The right ear had the lowest spirochete load (1.3 *23S rRNA* copies/10^6 *Beta-actin* copies) and the heart had the highest spirochete load (1537.8 (1.3 *23S rRNA* copies/10^6 *Beta-actin* copies). If the right ear is taken as the reference tissue, the mean spirochete load in the kidney, left ear, ventral skin, joint, bladder, and heart is 3.2x, 8.0x, 14.3x, 32.6x, 76.9x, 824.2x, and 1182.9x higher, respectively. Thus, the mean spirochete load in the skin-related organs (ventral skin, left ear, right ear) was orders of magnitude lower compared to the mean spirochete load in the internal organs (bladder, and heart). Again, we point out the remarkable change in spirochete load in the right ear from ear biopsy 3 on day 89 PI (812 *23S rRNA* copies/10^6 *Beta-actin* copies) to the necropsy on day 97 PI (1.3 *23S rRNA* copies/10^6 *Beta-actin* copies). In other words, over the course of 8 days, the mean spirochete load in the right ear decreased 624.6-fold.

Explanation for decrease in infection prevalence and spirochete load in right ear: The right ear was sampled with a biopsy on day 89 PI and a necropsy on day 97 PI. Over the course of 8 days, the infection prevalence of the right ear decreased 6.4-fold and the spirochete load of the right ear decreased 624.6-fold. The mice were injected with ketamine and xylazine on day 90 PI before being infested with I. *scapularis* larvae. A recent study has demonstrated that ketamine kills and inhibits the growth of *B. burgdorferi* in vitro [17]. Thus, one explanation is that the ketamine injection on day 90 PI was responsible for the 6.4-fold decrease in infection prevalence and for the 624.6-fold decrease in spirochete load in the right ear from day 89 PI to day 97 PI.

Table S3. Infection prevalence and mean spirochete load in the mouse tissues. For each of the 10 mouse tissues, the following are shown: number of infected tissues and the total number of tissues tested (Inf/Total), % of infected tissues, mean spirochete load on the log[X + 1] scale (Mean1), standard deviation (SD1), and standard error (SE1; based on sample size of 84), and lower limit (LL1) and upper limit (UL1) of the 95% confidence interval.

Tissue	Inf/Total	% inf	Mean1	SD1	SE1	LL1	UL1	Mean2	LL2	UL2
Biopsy 1	83/84	98.8	3.459	0.800	0.088	3.285	3.634	2878.5	1925.5	4302.9
Biopsy 2	84/84	100.0	3.019	0.405	0.044	2.931	3.108	1044.2	851.9	1280.0
Biopsy 3	77/84	91.7	2.910	0.962	0.106	2.700	3.120	812.0	500.3	1317.5
Ventral	44/84	52.4	1.637	1.584	0.174	1.291	1.983	42.4	18.6	95.2
Ear left	32/84	38.1	1.057	1.134	0.125	0.809	1.304	10.4	5.4	19.2
Ear right	12/84	14.3	0.353	0.710	0.078	0.198	0.508	1.3	0.6	2.2
Heart	78/84	92.9	3.187	0.806	0.088	3.011	3.363	1537.8	1025.4	2306.1
Bladder	82/84	97.6	3.030	0.796	0.087	2.857	3.204	1071.4	717.7	1599.0
Joint	60/84	71.4	2.004	1.201	0.132	1.742	2.266	99.9	54.2	183.6
Kidney	25/84	29.8	0.714	0.909	0.100	0.516	0.913	4.2	2.3	7.2

Section 4 – Analyses of the larval infection prevalence (LIP)

GLMM of LIP: The larval infection prevalence (LIP) is the proportion of engorged larvae infected with *B. burgdorferi*. The analysis of the LIP was performed on all the engorged larvae (n = 950 engorged larvae) that fed on the subset of infected mice (n = 84 mice). The response variable was the infection status for each engorged larva as measured by *23S rRNA* qPCR. This binomial response variable (0 = uninfected, 1 = infected) was modelled using a generalized linear mixed effect model (GLMM) with binomial errors. The fixed factors were strain (11 levels), mouse sex (2 levels: male, female), infestation (3 levels: infestation 1, infestation 2, infestation 3), and temporal block (2 levels: A and B). Tick *calreticulin* gene copy number was not included in the model. The full model included all the two-way and the three-way interactions between strain, sex, and infestation. Temporal block was included as a main effect only. Mouse identity was included as a random factor. Non-significant interaction terms (at $\alpha = 0.05$) were removed sequentially (Table S4). The final simplified model included no interactions (Table S4).

Strain ($p = 1.346*10^{-15}$), infestation ($p = 3.359*10^{-5}$), and temporal block ($p = 8.088*10^{-10}$) but not mouse sex (p = 0.085) had significant effects on the LIP (Table S4). The parameter estimates for the final simplified model are shown in Table S5. The mean LIP for the levels of a given factor were calculated from the raw data by averaging over the levels of the other factors (Table S6). Estimated marginal means (EMMs) of the LIP and post-hoc tests were generated using the ggemmeans() function and the final simplified model (Table S6). The means from the raw data and the EMMs were similar (Table S6). The Pearson correlation between the means and the EMMs of the LIP across the 18 levels was positive and significant (Mean vs EMM in Table S6; r = 0.977, t = 18.36, df = 16, p = 3.563e-12). In what follows, we present the EMMs of the LIP and their post-hoc tests (Table S6).

Among strains of *B. burgdorferi* and averaged across mouse sex, infestation, and block, the mean LIP ranged from 32.89% for strain 54 to 97.10% for strain 178-1. All strains, except strain 54, had a significantly higher LIP compared to the reference strain 66 (Table S5). Averaged across *B. burgdorferi* strains, infestation, and block, the EMM LIP for male mice (79.72%) was higher compared to female mice (73.56%), but this difference was not significant (p = 0.085). Averaged across *B. burgdorferi* strains, mouse sex, and block, the EMM LIP for infestation 1 (88.96%) was significantly higher compared to infestation 2 (65.88%; p < 0.0001) and infestation 3 (69.94%; p = 0.0002). The EMM LIP for block B (86.48%) was significantly higher compared to block A (63.10%; p < 0.0001).

GLMM of LIP that exclude infestation 1 or block A: For infestation 1, engorged larvae were collected for block B, but not block A. We therefore re-analyzed the LIP for the data set that excluded infestation 1. The results were like Table S4; strain ($p = 1.302*10^{-13}$) and block ($3.777*10^{-09}$) remained highly significant, but infestation was no longer significant (p = 0.259). This result was expected because the significant effect of infestation was driven by differences between infestation 1 versus the other two infestations. We also re-analyzed the LIP for the data set that excluded block A. The results were like Table S4; strain ($p = 1.031*10^{-10}$) and infestation ($4.123*10^{-5}$) remained highly significant. In summary, the significant effects of infestation and temporal block on the LIP were independent of the uneven sampling of engorged larvae from infestation 1 between temporal blocks A and B.

Differences between temporal blocks: The difference in LIP between the two temporal blocks (63.10% in block A versus 86.48% in block B) was unexpected. Importantly, as the main factors of interest, *B. burgdorferi* strain and mouse sex, were perfectly orthogonal with respect to block, differences between blocks do not influence differences between strains or the sexes. We also emphasize here that there was no effect of temporal block on the NIP and NSL. Thus, whatever factors caused the differences in LIP and LSL between temporal blocks, their importance disappeared once the larvae moulted into nymphs.

LMM of tick *calreticulin*: We used an LMM to compare the tick *calreticulin* gene copy number between blocks. This analysis found that engorged larvae from block B contained significantly more *calreticulin* genes compared to engorged larvae in block A ($p < 2e-16$). One explanation is that there was variation in DNA extraction efficiency between the two blocks. We view this explanation as unlikely because all larvae had their DNA extracted at the same time. A second explanation is variation in quality of *I. scapularis* larvae that we received from Oklahoma State University. This laboratory colony is periodically outbred with wild *I. scapularis* ticks to maintain genetic variation. Over the 2-year duration of this study, we performed at least 8 different larval infestations, and we observed substantial variation in the quality and vigour of the larvae. Some batches of larvae were lively and motivated to feed, whereas other batches lacked vigour. In summary, variation among batches of larvae may have caused the observed differences in tick *calreticulin* gene copy number, LIP, and LSL. Importantly, these differences among blocks had no effect on the NIP and NSL.

Table S4. Model simplification of the GLMM of LIP. Non-significant interactions were sequentially removed in order of increasing significance; the model was updated each time a non-significant term was removed. The final simplified model contained the fixed factors only. For each term, the order in which it was removed, the Chi-squared statistic (Chi), degrees of freedom, and p-value (p) are shown. For the non-significant interactions, the p-values are the ones in the model from which they were removed. For the included terms, the p-values are from the final simplified model.

Term	Order removed	Chi	df	p	Signif [a]
Strain:Sex:Infestation	1	8.738	19	0.978	
Strain:Infestation	2	12.306	20	0.905	
Sex:Infestation	3	3.184	2	0.203	
Strain:Sex	4	14.660	10	0.145	
Strain	Final model	93.020	10	$1.346*10^{-15}$	***
Sex	Final model	2.965	1	0.085	
Infestation	Final model	20.603	2	$3.359*10^{-5}$	***
Temporal block	Final model	37.739	1	$8.088*10^{-10}$	***

[a] Categories of significance are as follows: * 0.05 < p > 0.01, ** 0.01 < p > 0.001, *** p < 0.001.

Table S5. Parameter estimates of the GLMM of LIP. Factors include strain (11 levels), mouse sex (2 levels: male, female), infestation (3 levels: infestation 1, infestation 2, infestation 3), and temporal block (2 levels: A and B). For each parameter in the GLMM, the type, description, estimate, standard error (SE), and p-value (p) are shown.

Parameter type	Parameter description	Estimate	SE	p	Signif [a]
Intercept	Reference [b]	-0.188	0.454	0.679	
Contrast	Strain 57 – Strain 66	2.149	0.468	$4.443*10^{-6}$	***
Contrast	Strain 150 – Strain 66	1.497	0.447	$8.163*10^{-4}$	***
Contrast	Strain 167 – Strain 66	1.666	0.436	$1.316*10^{-4}$	***
Contrast	Strain 126 – Strain 66	1.536	0.447	$5.888*10^{-4}$	***
Contrast	Strain 22-2 – Strain 66	1.289	0.451	0.004	**
Contrast	Strain 10-2 – Strain 66	2.015	0.480	$2.666*10^{-5}$	***
Contrast	Strain 54 – Strain 66	-0.122	0.448	0.785	
Contrast	Strain 198 – Strain	2.924	0.498	$4.379*10^{-9}$	***
Contrast	Strain 174 – Strain 66	2.604	0.542	$1.538*10^{-6}$	***
Contrast	Strain 178-1 – Strain 66	4.103	0.649	$2.564*10^{-10}$	***
Contrast	Female – Male	-0.346	0.201	0.085	
Contrast	Inf 2 – Inf 1	-1.428	0.315	$5.988*10^{-6}$	***
Contrast	Inf 3 – Inf 1	-1.242	0.311	$6.581*10^{-5}$	***
Contrast	Block B – Block A	1.319	0.215	$8.088*10^{-10}$	***

[a] Categories of significance are as follows: * 0.05 < p > 0.01, ** 0.01 < p > 0.001, *** p < 0.001.

[b] Reference condition refers to engorged larvae from infestation 1 that fed on a male mouse in block A that was infected with *B. burgdorferi* strain 66.

Table S6. The means and EMMs of the LIP for each of the levels of the factors of interest. The LIP is the percentage of engorged larvae infected with *B. burgdorferi*. The means are from the raw data, whereas the EMMs were calculated using the final simplified model in Table S4. The mean or EMM of a given level was averaged over the levels of the other factors. Lower and upper (LCL, UCL) 95% confidence limits for the EMM are reported.

Factor	Level	Mean (%)	EMM (%)	LCL (%)	UCL (%)
Strain	66	35.06	35.64	22.33	51.60
Strain	57	77.78	82.61	70.97	90.23
Strain	150	64.89	71.21	57.41	81.95
Strain	167	69.81	74.55	62.34	83.83
Strain	126	64.44	72.01	58.33	82.54
Strain	22-2	63.86	66.77	52.04	78.81
Strain	10-2	72.97	80.59	67.50	89.25
Strain	54	35.48	32.89	21.14	47.24
Strain	198	86.46	91.15	83.11	95.57
Strain	174	81.03	88.21	76.20	94.59
Strain	178-1	95.51	97.10	91.76	99.02
Sex	Male	71.40	79.72	73.97	84.47

Sex	Female	64.00	73.56	66.79	79.38
Infestation	Inf 1	89.67	88.96	81.88	93.49
Infestation	Inf 2	60.54	65.88	59.33	71.88
Infestation	Inf 3	64.65	69.94	63.73	75.49
Block	A	51.28	63.10	54.94	70.57
Block	B	79.57	86.48	82.17	89.88

Section 5 – Analyses of the larval spirochete load (LSL)

LMM of the LSL: The larval spirochete load (LSL) is the number of *23S rRNA* copies per infected engorged larvae. The analysis of the LSL was performed on the subset of engorged larvae that were infected with *B. burgdorferi* (n = 645 infected engorged larvae) that fed on the subset of infected mice (n = 84 mice). The LSL was log10-transformed to normalize the residuals. The $\log_{10}$ *23S rRNA* copies per engorged larva was modelled using a linear mixed effect model (LMM). The fixed factors were strain (11 levels), mouse sex (2 levels: male, female), infestation (3 levels: infestation 1, infestation 2, infestation 3), and temporal block (2 levels: A and B). The $\log_{10}$ *calreticulin* copies per tick was included as a covariate. The full model included all the two-way and three-way interactions between strain, sex, and infestation. Temporal block was included as a main effect only. Mouse identity was included as a random factor. The three-way interaction between strain, sex, and infestation was significant (p = 0.010), but none of the two-way interactions were significant (Table S7). A comparison of the AIC values found that the main effects model had an AIC value (1633.779) that was 36.4 units lower compared to the full interaction model (1670.186). For this reason, the parameter estimates and EMMs are presented for the more parsimonious main effects model (i.e., does not include any interactions; Table S8).

Strain ($p = 5.968*10^{-12}$), infestation ($p = 4.773*10^{-11}$), temporal block ($p = 6.236*10^{-05}$), and log10 *calreticulin* ($p = 2.2*10^{-16}$), but not mouse sex (p = 0.317) had significant effects on the LSL (Table S7). The parameter estimates for this final simplified model and their significance are shown in Table S8. Means and EMMs (using the final model) were generated for each factor level (Table S9). The means from the raw data and the EMMs were similar (Table S9). The Pearson correlation between the means and the EMMs of the LSL across the 18 levels was positive and significant (Mean1 vs EMM1 in Table S9; r = 0.944, t = 11.494, df = 16, p = 3.829e-09). The units of the LSL are the number of *23S rRNA* gene copies per infected engorged larva. In what follows, we present the EMMs of the LSL and their post-hoc tests (EMM2 in Table S9).

The EMM LSL varied 11.8-fold among strains from 728.5 units for strain 54 to 15635.5 units for strain 178-1. Larvae that fed on female mice had an EMM LSL (4094.7 units) that was 23.3% higher compared to larvae that fed on male mice (3320.7 units), but this difference was not significant (p = 0.322). The EMM LSL of infestation 1 (8502.2 units) was significantly higher compared to infestation 2 (2142.2 units; p < 0.0001) and infestation 3 (2752.9 units; p < 0.0001). Finally, the EMM LSL of larvae in block B (5808.5 units) was 2.5x higher compared to block A (2340.9 units; p = 0.0001).

The LSL had a positive and significant relationship with the number of *calreticulin* gene copies in the tick (Table S8: slope = 0.735, SE = 0.058, $p < 2.2*10^{-16}$). Thus, DNA extractions from engorged larvae that had higher levels of *calreticulin* also had higher levels of *23S rRNA*

(Figure S1). One methodological explanation for this result is variation in DNA extraction efficiency; high efficiency samples with more DNA will have more *calreticulin* and more *23S rRNA* gene copies. A biological explanation for this result is variation in larval size; larger larvae (as estimated by a higher abundance of *calreticulin*) may acquire more spirochetes during their blood meal [18]. A separate analysis of the ratio of the *23S rRNA* gene copy number to the tick *calreticulin* copy number provided very similar results (see below).

GLMM of LSL that exclude infestation 1 or block A: For infestation 1, engorged larvae were collected for block B, but not block A. We therefore re-analyzed the LSL for the data set that excluded infestation 1. The results were like Table S7; strain ($p = 1.015*10^{-06}$) and block ($1.280*10^{-4}$) remained highly significant, but infestation was no longer significant ($p = 0.146$). This result was expected because the significant effect of infestation was driven by differences between infestation 1 versus the other two infestations. We also re-analyzed the LSL for the data set that excluded block A. The results were like Table S7; strain ($p = 1.412*10^{-13}$), infestation ($1.125*10^{-11}$), and log10(*calreticulin*) ($2.2*10^{-16}$) remained highly significant. In summary, the significant effects of infestation and temporal block on the LSL were independent of the uneven sampling of engorged larvae from infestation 1 between temporal blocks A and B.

Table S7. Model simplification of the LMM of the LSL. Non-significant interactions were sequentially removed in order of increasing significance; the model was updated each time a term was removed. The final simplified model contained the fixed factors only. For each term, the order in which it was removed, the Chi-squared statistic (Chi), degrees of freedom (df), and p-value (p) are shown. For the non-significant interactions, the p-values are the ones in the model from which they were removed. For the included terms, the p-values are from the final simplified model.

Term	Order removed	Chi	df	p	Signif [a]
Strain:Sex:Infestation	1	36.180	19	0.010	*
Sex:Infestation	2	0.052	2	0.974	
Strain:Sex	3	7.160	10	0.710	
Strain:Infestation	4	27.011	20	0.135	
Strain	Final model	74.494	10	$5.968*10^{-12}$	***
Sex	Final model	1.002	1	0.317	
Infestation	Final model	47.531	2	$4.773*10^{-11}$	***
Temporal block	Final model	16.030	1	$6.236*10^{-5}$	***
log10(*calreticulin*)	Final model	162.05	1	$2.2*10^{-16}$	***

[a] Categories of significance are as follows: * 0.05 < p > 0.01, ** 0.01 < p > 0.001, *** p < 0.001.

Table S8. Parameter estimates of the LMM of the LSL. Factors include strain (11 levels), mouse sex (2 levels: male, female), and infestation (3 levels: infestation 1, infestation 2, infestation 3), temporal block (2 levels: A and B). Covariates included $\log_{10}$ *calreticulin* copies per tick. For each parameter in the LMM, the type, description, estimate, standard error (SE), and p-value (p) are shown.

Parameter type	Parameter description	Estimate	SE	p	Signif [a]
Intercept	Reference [b]	0.362	0.324	0.676	

Contrast	Strain 57 – Strain 66	0.286	0.242	0.241	
Contrast	Strain 150 – Strain 66	-0.443	0.247	0.077	
Contrast	Strain 167 – Strain 66	0.092	0.237	0.701	
Contrast	Strain 126 – Strain 66	0.126	0.246	0.609	
Contrast	Strain 22-2 – Strain 66	0.151	0.248	0.544	
Contrast	Strain 10-2 – Strain 66	0.277	0.250	0.270	
Contrast	Strain 54 – Strain 66	-0.518	0.266	0.054	
Contrast	Strain 198 – Strain 66	0.591	0.237	0.015	*
Contrast	Strain 174 – Strain 66	0.671	0.261	0.012	*
Contrast	Strain 178-1 – Strain 66	0.813	0.237	0.001	**
Contrast	Female – Male	0.091	0.091	0.321	
Contrast	Inf 2 – Inf 1	-0.599	0.089	$4.118*10^{-11}$	***
Contrast	Inf 3 – Inf 1	-0.490	0.090	$8.939*10^{-8}$	***
Contrast	Block B – Block A	0.395	0.099	$1.349*10^{-4}$	***
Slope	log10(*calreticulin*)	0.735	0.058	$2.2*10^{-16}$	***

[a] Categories of significance are as follows: * 0.05 < p > 0.01, ** 0.01 < p > 0.001, *** p < 0.001.

[b] Reference condition refers to engorged larvae from infestation 1 that fed on a male mouse in block A that was infected with *B. burgdorferi* strain 66. The value of the covariate log10(*calreticulin*) was set to 0.

Table S9. The means and EMMs of the LSL for each of the levels of the factors of interest. The LSL is the number of *23S rRNA* gene copies per infected engorged larva (i.e., uninfected larvae are excluded). The means are from the raw data, whereas the EMMs were calculated using the final simplified model in Table S7. The mean or EMM of a given level was averaged over the levels of the other factors. For each EMM, the values of log10 *calreticulin* copies per tick were set to 4.57. Mean 1, EMM1, are on the log10-transformed scale, whereas Mean 2 and EMM2 are on the original scale. LCL and UCL refer to the 95% lower and upper confidence limits reported on the same scale.

Factor	Level	Mean1	Mean 2	EMM1	LCL1	UCL1	EMM2	LCL2	UCL2
Strain	66	3.572	3731.1	3.381	2.992	3.769	2402.6	982.5	5875.0
Strain	57	3.698	4986.9	3.667	3.389	3.945	4644.0	2449.4	8804.9
Strain	150	3.107	1280.1	2.937	2.642	3.233	865.6	438.1	1710.3
Strain	167	3.565	3672.5	3.472	3.213	3.731	2966.2	1634.7	5382.2
Strain	126	3.571	3727.2	3.507	3.216	3.799	3214.3	1642.8	6289.3
Strain	22-2	3.522	3323.1	3.532	3.234	3.829	3403.3	1715.2	6753.0
Strain	10-2	3.532	3402.1	3.658	3.356	3.960	4548.9	2270.8	9112.2
Strain	54	3.120	1317.4	2.862	2.511	3.213	728.5	324.7	1634.5
Strain	198	3.924	8402.2	3.972	3.714	4.230	9379.0	5174.1	17001.1
Strain	174	4.008	10189.2	4.051	3.717	4.385	11254.3	5213.9	24292.8

Strain	178-1	4.194	15635.5	4.194	3.937	4.451	15635.5	8645.3	28277.6
Sex	Male	3.633	4293.4	3.521	3.395	3.647	3320.7	2483.0	4441.1
Sex	Female	3.715	5182.3	3.612	3.476	3.748	4094.7	2995.6	5597.0
Infestation	Inf 1	4.021	10501	3.930	3.771	4.088	8502.2	5906.7	12238.2
Infestation	Inf 2	3.325	2115.3	3.331	3.205	3.457	2142.2	1603.1	2862.5
Infestation	Inf 3	3.743	5538.3	3.440	3.317	3.563	2752.9	2073.8	3654.2
Block	A	3.271	1867.1	3.369	3.215	3.524	2340.9	1640.9	3339.6
Block	B	3.850	7071.7	3.764	3.649	3.880	5808.5	4451.9	7578.5

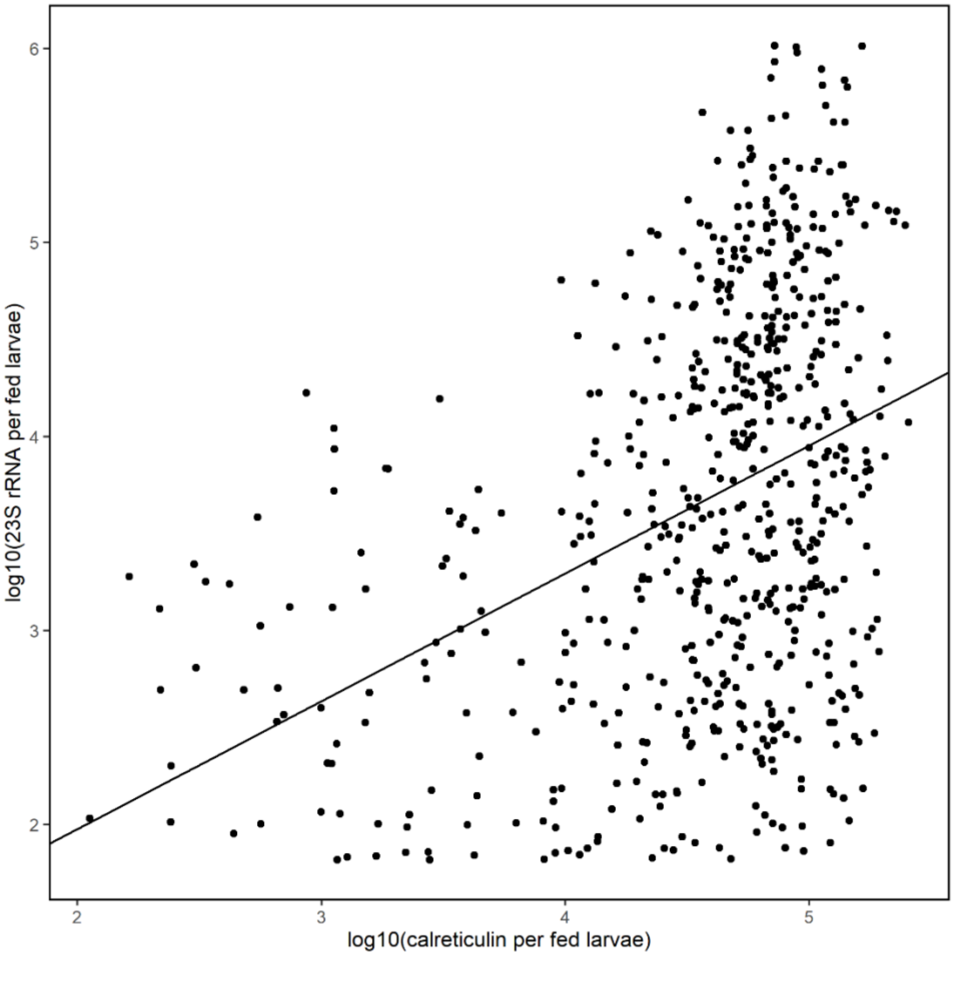

Figure S1. The larval spirochete load (LSL) in engorged larvae increased with the tick *calreticulin* load. The LSL was measured as the $\log_{10}$ *23S rRNA* copies per tick. The *calreticulin* load was measured as the $\log_{10}$ *calreticulin* copies per tick. Each point represents an individual infected engorged larva (n = 645). The line represents the simple linear regression (slope = 0.657, r = 0.38, p = $2.2*10^{-16}$).

Analysis of LSL standardized to *calreticulin*: In the previous analysis of LSL, the tick *calreticulin* gene copy number was included as a covariate. In the present analysis, the response variable was the ratio of *23S rRNA* gene copies per million tick *calreticulin* gene copies per tick (LSLCAL). This ratio was log10 transformed to standardize the residuals. As before, this analysis was performed on the subset of engorged larvae that were infected with *B. burgdorferi* (n = 645 engorged larvae) that fed on the subset of infected mice (n = 84 mice). The LSLCAL was modelled using an LMM. The fixed factors were strain (11 levels), mouse sex (2 levels: male, female), infestation (3 levels: infestation 1, infestation 2, infestation 3), and temporal block (2 levels: A and B). The full model included all the two-way and three-way interactions between strain, sex, and infestation. Temporal block was included as a main effect only. Mouse identity was included as a random factor. As before, the three-way interaction between strain, sex, and infestation was significant (p = 0.013), but none of the two-way interactions were significant (Table S10). For simplicity, we interpreted the parameter estimates for the main effects model (i.e., all interactions were removed).

Strain ($p = 1.462*10^{-12}$), infestation ($p = 1.229*10^{-13}$), and temporal block ($p = 6.565*10^{-04}$), but not mouse sex (p = 0.406) had significant effects on the LSLCAL (Table S10). The parameter estimates for this final simplified model and their significance are shown in Table S11. The correlation in the parameter estimates of the 14 contrasts between Table S8 and Table S11 is positive and highly significant (r = 0.996, df = 12, t = 36.703, p = 1.073E-13). Thus, the results from these two analyses are very similar regardless of whether tick *calreticulin* gene copy number is included as an explanatory covariate in the analysis of the LSL (Table S7, S8) or in the denominator of the LSLCAL response variable (Table S10, Table S11).

Table S10. Model simplification of the LMM of the LSLCAL. Non-significant interactions were sequentially removed in order of increasing significance; the model was updated each time a term was removed. The final simplified model contained the fixed factors only. For each term, the order in which it was removed, the Chi-squared statistic (Chi), degrees of freedom (df), and p-value (p) are shown. For the non-significant interactions, the p-values are the ones in the model from which they were removed. For the included terms, the p-values are from the final simplified model.

Term	Order removed	Chi	df	p	Signif [a]
Strain:Sex:Infestation	1	35.141	19	0.013	*
Sex:Infestation	2	0.145	2	0.930	
Strain:Sex	3	8.022	10	0.627	
Strain:Infestation	4	26.595	20	0.147	
Strain	Final model	77.628	10	$1.462*10^{-12}$	***
Sex	Final model	0.692	1	0.406	
Infestation	Final model	59.454	2	$1.229*10^{-13}$	***
Temporal block	Final model	11.608	1	$6.565*10^{-4}$	***

[a] Categories of significance are as follows: * 0.05 < p > 0.01, ** 0.01 < p > 0.001, *** p < 0.001.

Table S11. Parameter estimates of the LMM of the LSLCAL. Factors include strain (11 levels), mouse sex (2 levels: male, female), and infestation (3 levels: infestation 1, infestation 2,

infestation 3), temporal block (2 levels: A and B). For each parameter in the LMM, the type, description, estimate, standard error (SE), and p-value (p) are shown.

Parameter type	Parameter description	Estimate	SE	p	Signif [a]
Intercept	Reference [b]	4.986	0.229	$2.2*10^{-16}$	***
Contrast	Strain 57 – Strain 66	0.329	0.248	0.188	
Contrast	Strain 150 – Strain 66	-0.439	0.253	0.087	
Contrast	Strain 167 – Strain 66	0.141	0.242	0.563	
Contrast	Strain 126 – Strain 66	0.176	0.251	0.486	
Contrast	Strain 22-2 – Strain 66	0.203	0.254	0.426	
Contrast	Strain 10-2 – Strain 66	0.341	0.255	0.184	
Contrast	Strain 54 – Strain 66	-0.498	0.272	0.070	
Contrast	Strain 198 – Strain 66	0.662	0.242	0.008	**
Contrast	Strain 174 – Strain 66	0.724	0.266	0.008	**
Contrast	Strain 178-1 – Strain 66	0.872	0.242	0.001	**
Contrast	Female – Male	0.077	0.093	0.409	
Contrast	Inf 2 – Inf 1	-0.622	0.090	$1.418*10^{-11}$	***
Contrast	Inf 3 – Inf 1	-0.619	0.087	$3.836*10^{-12}$	***
Contrast	Block B – Block A	0.341	0.100	0.001	**

[a] Categories of significance are as follows: * 0.05 < p > 0.01, ** 0.01 < p > 0.001, *** p < 0.001.

[b] Reference condition refers to engorged larvae from infestation 1 that fed on a male mouse in block A that was infected with *B. burgdorferi* strain 66.

Section 6 – Analyses of the nymphal infection prevalence (NIP)

GLMM of NIP: The nymphal infection prevalence (NIP) is the proportion of unfed nymphs infected with *B. burgdorferi*. The analysis of the NIP was performed on all the unfed nymphs (n = 2471 unfed nymphs) that fed as larvae on the subset of infected mice (n = 84 mice). The response variable was the infection status for each nymph as measured by *23S rRNA* qPCR. This binomial response variable (0 = uninfected, 1 = infected) was modelled using a GLMM with binomial errors. The fixed factors were strain (11 levels), mouse sex (2 levels: male, female), infestation (3 levels: infestation 1, infestation 2, infestation 3), and temporal block (2 levels: A and B). The full model included all the two-way and the three-way interactions between strain, sex, and infestation. Temporal block was included as a main effect only. Mouse identity was included as a random factor. Non-significant, or non-functional interaction terms were removed sequentially (Table S12). Significant interactions were removed if they had standard errors that were orders of magnitude greater than the parameter estimates. Such problem interactions usually have marginally significant p-values (i.e., 0.01 < p < 0.05). Retention of such problem interactions in the model results in biased estimates of the EMMs.

The interaction between strain and sex (p = 0.022) and between strain and infestation (p = 0.017) were examples of problem interactions. Although these interactions were both statistically

significant according to the Wald chi-square tests, inspection of the parameter estimates found that the contrasts for some strains had standard errors that were an order of magnitude larger than the parameter estimates. For this reason, these interactions were removed from the model. The final simplified model included the main effects and the interaction between sex and infestation (Table S12). The interaction between sex and infestation had a significant effect on the NIP ($1.57*10^{-4}$). Strain ($p = 1.835*10^{-14}$), mouse sex ($p = 9.76*10^{-4}$), infestation ($p = 2.2*10^{-16}$), but not temporal block ($p = 0.568$) had significant effects on the NIP (Table S12).

The parameter estimates for the final simplified model and their significance are shown in Table S13. The mean NIP for the levels of a given factor were calculated from the raw data by averaging over the other levels (Table S14). Estimated marginal means (EMMs) of NIP were generated for each factor level using the ggemmeans() function and the final simplified model (Table S14). The means from the raw data and the EMMs were similar (Table S14). The Pearson correlation between the means and the EMMs of the NIP across the 24 levels was positive and significant (Mean vs EMM in Table S14; $r = 0.953$, $t = 14.806$, $df = 22$, $p = 6.379e-13$). In what follows, we present the EMMs of the NIP and their post-hoc tests (Table S14).

Among strains of *B. burgdorferi* and averaged across mouse sex, infestation, and block, the EMM NIP ranged from 57.53% for strain 66 to 99.46% for strain 178-1. All strains, except strain 54, had a significantly higher NIP compared to reference strain 66 (Table S13). Averaged across *B. burgdorferi* strains, infestation, and block, the EMM NIP for male mice (95.30%) was higher compared to female mice (93.19%), but this difference was not significant ($p = 0.157$). Averaged across *B. burgdorferi* strains, mouse sex, and block, the EMM NIP for infestation 1 (98.85%) was significantly higher compared to infestation 2 (90.18%; $p < 0.0001$) and infestation 3 (85.40%; $p < 0.0001$). The EMM NIP for block B (94.71%) was similar to block A (93.94%; $p = 0.565$).

The interaction between sex and infestation refers to the fact that the difference in NIP between males and females changes over the successive infestations (Figure 2 in the main manuscript). For infestation 1, males have a lower EMM NIP compared to females (98.29% vs 99.23%, $p = 0.095$) but the difference is not significant. Males have a significantly higher NIP compared to females for infestation 2 (93.22% vs 86.00%, $p = 0.008$) and infestation 3 (91.33% vs 76.45%, $p < 0.0001$).

The relationship between NIP and infestation is shown for each of the 11 *B. burgdorferi* strains in Figure 1 in the main manuscript and in Figure S2. In this study, the NIP is synonymous with host-to-tick transmission (HTT), but the latter term has as stronger connotation of being a pathogen life history trait. For this reason, the NIP is labelled as host-to-tick transmission (HTT) in Figure S2. Figure S2 shows the estimated marginal means and their 95% confidence intervals that were calculated for the 33 combinations of strain and infestation using the final simplified model and the ggemmeans() function.

Table S12. Model simplification of the GLMM of the NIP. Non-significant interactions were sequentially removed in order of increasing significance; the model was updated each time a term was removed. Significant interactions were removed if they had standard errors that were orders of magnitude greater than the parameter estimates. The final simplified model contained the fixed factors and the interaction of sex and infestation. For each term, the order in which it was removed, the Chi-squared statistic (Chi), degrees of freedom (df), and p-value (p) are shown. For the non-significant interactions, the p-values are the ones in the model from which they were removed. For the included terms, the p-values are from the final simplified model.

Term	Order removed	Chi	df	p	Signif [a]
Strain:Sex:Infestation	1	17.386	20	0.628	
Strain:Sex	2	20.847	10	0.022	*
Strain:Infestation	3	35.616	20	0.017	*
Sex:Infestation	Final model	17.520	2	$1.57*10^{-4}$	***
Strain	Final model	87.300	10	$1.835*10^{-14}$	***
Sex	Final model	10.873	1	$9.76*10^{-4}$	***
Infestation	Final model	111.972	2	$2.2*10^{-16}$	***
Temporal block	Final model	0.326	1	0.568	

[a] Categories of significance are as follows: * 0.05 < p > 0.01, ** 0.01 < p > 0.001, *** p < 0.001.

Table S13. Parameter estimates of the GLMM of the NIP. Factors include strain (11 levels), mouse sex (2 levels: male, female), infestation (3 levels: infestation 1, infestation 2, infestation 3), and temporal block (2 levels: A and B). For each parameter in the GLMM, the type, description, estimate, standard error (SE), and p-value (p) are shown.

Parameter type	Parameter description	Estimate	SE	p	Signif [a]
Intercept	Reference [b]	1.471	0.449	0.001	**
Contrast	Strain 57 – Strain 66	2.826	0.559	$4.336*10^{-7}$	***
Contrast	Strain 150 – Strain 66	2.252	0.537	$2.783*10^{-5}$	***
Contrast	Strain 167 – Strain 66	2.059	0.529	$9.906*10^{-5}$	***
Contrast	Strain 126 – Strain 66	2.508	0.541	$3.485*10^{-6}$	***
Contrast	Strain 22-2 – Strain 66	1.641	0.535	0.002	**
Contrast	Strain 10-2 – Strain 66	2.185	0.532	$4.041*10^{-5}$	***
Contrast	Strain 54 – Strain 66	0.741	0.508	0.144	
Contrast	Strain 198 – Strain 66	3.585	0.602	$2.666*10^{-9}$	***
Contrast	Strain 174 – Strain 66	4.902	0.945	$2.104*10^{-7}$	***
Contrast	Strain 178-1 – Strain 66	4.906	0.779	$3.043*10^{-10}$	***
Contrast	Female – Male	0.805	0.483	0.095	
Contrast	Inf 2 – Inf 1	-1.432	0.291	$8.405*10^{-7}$	***
Contrast	Inf 3 – Inf 1	-1.698	0.286	$2.902*10^{-9}$	***
Contrast	Block B – Block A	0.145	0.253	0.565	
Contrast	Female Inf 2 – Male Inf 1	-1.611	0.477	0.001	**
Contrast	Female Inf 3 – Male Inf 1	-1.982	0.475	$2.950*10^{-5}$	***

[a] Categories of significance are as follows: * 0.05 < p > 0.01, ** 0.01 < p > 0.001, *** p < 0.001.

[b] Reference condition refers to engorged larvae from infestation 1 that fed on a male mouse in block A that was infected with *B. burgdorferi* strain 66.

Table S14. The means and EMMs of the NIP for each of the levels of the factors of interest. The NIP is the percentage of unfed nymphs infected with *B. burgdorferi* (these nymphs took their

larval blood meal from the experimentally infected mice). The means are from the raw data, whereas the EMMs were calculated using the final simplified model in Table S12. The mean or EMM of a given level was averaged over the levels of the other factors. Lower and upper (LCL, UCL) 95% confidence limits for the EMM are reported. For factors with significant interactions, the means and EMMs are reported for the relevant combinations of factors.

Factor	Level	Mean (%)	EMM (%)	LCL (%)	UCL (%)
Strain	66	54.29	57.53	39.63	73.65
Strain	57	92.17	95.81	90.86	98.13
Strain	150	85.77	92.79	85.58	96.54
Strain	167	83.40	91.39	83.31	95.76
Strain	126	88.98	94.33	88.37	97.33
Strain	22-2	77.88	87.49	76.47	93.76
Strain	10-2	86.27	92.33	84.92	96.26
Strain	54	67.39	73.98	58.77	85.00
Strain	198	95.42	97.99	95.02	99.21
Strain	174	98.67	99.45	97.05	99.90
Strain	178-1	98.73	99.46	97.93	99.86
Sex	Male	87.78	95.30	93.00	96.87
Sex	Female	80.71	93.19	89.96	95.44
Infestation	Inf 1	96.28	98.85	98.09	99.31
Infestation	Inf 2	81.30	90.18	86.71	92.82
Infestation	Inf 3	75.15	85.40	80.81	89.04
Block	A	83.68	93.94	91.25	95.84
Block	B	85.01	94.71	92.17	96.47
Sex:Inf	Male & Inf 1	94.85	98.29	96.90	99.06
Sex:Inf	Male & Inf 2	85.75	93.22	89.50	95.68
Sex:Inf	Male & Inf 3	82.62	91.33	86.93	94.34
Sex:Inf	Female & Inf 1	97.78	99.23	98.34	99.65
Sex:Inf	Female & Inf 2	76.72	86.00	80.05	90.38
Sex:Inf	Female & Inf 3	67.10	76.45	68.30	83.03

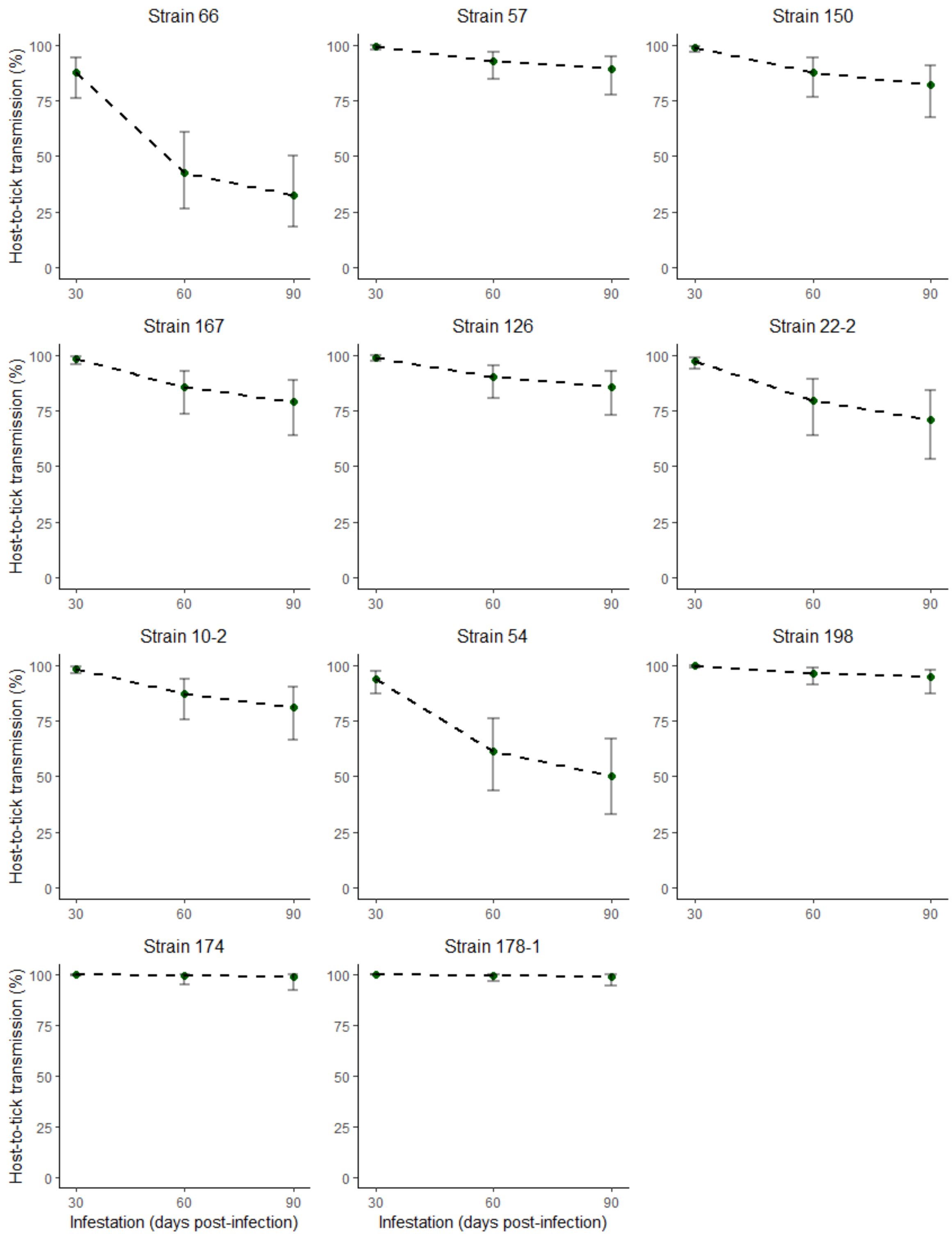

Figure S2. The lifetime host-to-tick transmission (HTT) of *B. burgdorferi* from infected mice to immature *I. scapularis* ticks decreased over the 3 successive larval infestations for some strains but remained constant over time for other strains. For this reason, the 2-way interaction between strain and infestation was significant ($p = 0.017$). Lifetime HTT was measured by infesting mice

with *I. scapularis* larvae on 3 occasions (days 30, 60, and 90 post-infection), allowing the engorged larvae to moult into nymphs, and testing the nymphs for infection with *B. burgdorferi*. The mean values (green circles) for each infestation are the EMMs from the GLMM for the HTT of each strain over time. Error bars are the 95% confidence intervals for the EMMs.

Section 7 – Analyses of the nymphal spirochete load (NSL)

LMM of the NSL: The nymphal spirochete load (NSL) is the number of *23S rRNA* copies per infected unfed nymph. The analysis of the NSL was performed on the subset of unfed nymphs that were infected with *B. burgdorferi* (n = 2,084 infected unfed nymphs) and that fed on the subset of infected mice (n = 84 mice). The NSL was log10-transformed to normalize the residuals. The $\log_{10}$ *23S rRNA* copies per unfed nymph was modelled using an LMM. The fixed factors were strain (11 levels), mouse sex (2 levels: male, female), infestation (3 levels: infestation 1, infestation 2, infestation 3), and temporal block (2 levels: A and B). The full model included all the two-way and the three-way interactions between strain, sex, and infestation. Temporal block was included as a main effect only. Mouse identity was included as a random factor. Non-significant interactions ($p > 0.05$) were removed sequentially (Table S15).

The final simplified model included the main effects and the interactions between strain and infestation, and sex and infestation (Table S15). The interaction between strain and infestation ($p = 0.0001$) and the interaction between sex and infestation ($p = 0.026$) both had significant effect on the NSL. Strain ($p = 2.2*10^{-16}$) and infestation ($p = 1.01*10^{-11}$), but not mouse sex ($p = 0.988$) and temporal block ($p = 0.182$), had significant effects on the NSL (Table S15).

The parameter estimates for this final simplified model and their significance are shown in Table S16. Means and EMMs (using the final model and the ggemmeans() function) of the NSL were generated for each factor level (Table S17). As there were significant interactions between strain and infestation, and sex and infestation, EMMs for the combinations of these factors were also estimated (Table S17). The means from the raw data and the EMMs were similar (Table S17). The Pearson correlation between the means and the EMMs of the NSL across the 57 levels was positive and significant (Mean1 vs EMM1 in Table S17; r = 0.997, t = 102.32, df = 55, p < 2.2e-16). The units of the NSL are the number of *23S rRNA* gene copies per infected unfed nymph. In what follows, we present the EMMs of the NSL and their post-hoc tests (EMM2 in Table S17).

The EMM NSL varied 8.9-fold among strains from 7665.2 units for strain 66 to 68086.7 units for strain 174. All the strains had a higher NSL compared to reference strain 66 (Table S16). The EMM NSL of infestation 1 (29631.2 units) was 59.5% higher compared to infestation 2 (18580.3 units; p < 0.0001) and 86.0% higher compared to infestation 3 (15934.6 units; p < 0.0001). The interaction between strain and infestation indicated that the NSL changed over the three infestations for some strains, but not for others (Figure S3).

Nymphs that had fed as larvae on male mice had an EMM NSL (20936.3 units) that was 3.0% higher compared to nymphs that had fed as larvae on female mice (20317.1 units), but this difference was not significant (p = 0.768). The interaction between sex and infestation refers to the fact that the difference in NSL between males and females changes over the successive infestations. For infestation 1, males have a lower EMM NSL compared to females (26270.5 vs 33421.9), but males have a higher EMM NSL compared to females for infestation 2 (19525.2 vs

17681.1) and infestation 3 (17891.0 vs 14192.1). Finally, the EMM NSL of larvae in block B (22017.2 units) was similar compared to block A (19319.7 units; p = 0.198).

Table S15. Model simplification of the LMM of the NSL. Non-significant interactions were sequentially removed in order of increasing significance; the model was updated each time a term was removed. For each term, the order in which it was removed, the Chi-squared statistic (Chi), degrees of freedom (df), and p-value (p) are shown. For the non-significant interactions, the p-values are the ones in the model from which they were removed. For the included terms, the p-values are from the final simplified model.

Term	Order removed	Chi	df	p	Signif [a]
Strain:Sex:Infestation	1	23.840	20	0.249	
Strain:Sex	2	4.014	10	0.947	
Sex:Infestation	Final model	7.318	2	0.026	*
Strain:Infestation	Final model	53.213	20	$7.561*10^{-5}$	***
Strain	Final model	215.753	10	$2.2*10^{-16}$	***
Sex	Final model	$1.0*10^{-4}$	1	0.992	
Infestation	Final model	50.887	2	$8.914*10^{-12}$	***
Temporal block	Final model	1.692	1	0.193	

[a] Categories of significance are as follows: * 0.05 < p > 0.01, ** 0.01 < p > 0.001, *** p < 0.001.

Table S16. Parameter estimates of the LMM of the NSL. Factors include strain (11 levels), mouse sex (2 levels: male, female), infestation (3 levels: infestation 1, infestation 2, infestation 3), and temporal block (2 levels: A and B). For each parameter in the LMM, the type, description, estimate, standard error (SE), and p-value (p) are shown.

Parameter type	Parameter description	Estimate	SE	p	Signif [a]
Intercept	Reference [b]	3.831	0.114	$2.2*10^{-16}$	***
Contrast	Strain 57 – Strain 66	0.236	0.145	0.104	
Contrast	Strain 150 – Strain 66	0.333	0.144	0.021	
Contrast	Strain 167 – Strain 66	0.440	0.144	0.002	
Contrast	Strain 126 – Strain 66	0.522	0.145	$3.812*10^{-4}$	***
Contrast	Strain 22-2 – Strain 66	0.665	0.143	$5.723*10^{-6}$	***
Contrast	Strain 10-2 – Strain 66	0.660	0.144	$7.061*10^{-6}$	***
Contrast	Strain 54 – Strain 66	0.656	0.145	$9.412*10^{-6}$	***
Contrast	Strain 198 – Strain 66	0.815	0.144	$4.130*10^{-8}$	***
Contrast	Strain 174 – Strain 66	1.012	0.161	$1.645*10^{-9}$	***
Contrast	Strain 178-1 – Strain 66	0.823	0.144	$3.186*10^{-8}$	***
Contrast	Female – Male	0.105	0.059	0.079	
Contrast	Inf 2 – Inf 1	-0.122	0.162	0.451	
Contrast	Inf 3 – Inf 1	0.217	0.191	0.256	
Contrast	Block B – Block A	0.057	0.044	0.198	
Contrast	57 Inf 2 - 66 Inf 1	0.009	0.199	0.965	

Contrast	150 Inf 2 - 66 Inf 1	-0.316	0.199	0.112	
Contrast	167 Inf 2 - 66 Inf 1	-0.101	0.203	0.618	
Contrast	126 Inf 2 - 66 Inf 1	0.115	0.200	0.566	
Contrast	22-2 Inf 2 - 66 Inf 1	-0.230	0.202	0.255	
Contrast	10-2 Inf 2 - 66 Inf 1	-0.157	0.201	0.436	
Contrast	54 Inf 2 - 66 Inf 1	-0.039	0.214	0.854	
Contrast	198 Inf 2 - 66 Inf 1	0.287	0.198	0.148	
Contrast	174 Inf 2 - 66 Inf 1	0.202	0.217	0.350	
Contrast	178-1 Inf 2 - 66 Inf 1	0.157	0.197	0.427	
Contrast	57 Inf 3 - 66 Inf 1	-0.254	0.226	0.261	
Contrast	150 Inf 3 - 66 Inf 1	-0.635	0.228	0.005	**
Contrast	167 Inf 3 - 66 Inf 1	-0.532	0.226	0.019	*
Contrast	126 Inf 3 - 66 Inf 1	-0.558	0.226	0.014	*
Contrast	22-2 Inf 3 - 66 Inf 1	-0.610	0.241	0.011	*
Contrast	10-2 Inf 3 - 66 Inf 1	-0.390	0.226	0.085	
Contrast	54 Inf 3 - 66 Inf 1	-0.746	0.237	0.002	**
Contrast	198 Inf 3 - 66 Inf 1	-0.015	0.222	0.947	
Contrast	174 Inf 3 - 66 Inf 1	-0.393	0.239	0.099	
Contrast	178-1 Inf 3 - 66 Inf 1	-0.093	0.222	0.674	
Contrast	Female Inf 2 – Male Inf 1	-0.148	0.077	0.055	
Contrast	Female Inf 3 – Male Inf 1	-0.205	0.080	0.011	*

[a] Categories of significance are as follows: * 0.05 < p > 0.01, ** 0.01 < p > 0.001, *** p < 0.001.

[b] Reference condition refers to engorged larvae from infestation 1 that fed on a male mouse in block A that was infected with *B. burgdorferi* strain 66.

Table S17. The means and EMMs of the NSL for each of the levels of the factors of interest. The NSL is the number of *23S rRNA* gene copies per infected unfed nymph (i.e., uninfected nymphs are excluded). The means are from the raw data, whereas the EMMs were calculated using the final simplified model in Table S15. The mean or EMM of a given level was averaged over the levels of the other factors. Mean 1 and EMM1 are on the log10-transformed scale, whereas Mean 2 and EMM2 are on the original scale. For factors with significant interactions, the means and EMMs are reported for the relevant combinations of factors. LCL and UCL represent the lower and upper 95% confidence intervals reported on the same scale.

Factor	Level	Mean1	Mean2	EMM1	LCL1	UCL1	EMM2	LCL2	UCL2
Strain	66	3.887	7707.3	3.885	3.706	4.063	7665.2	5077.9	11570.8
Strain	57	4.041	10997.7	4.038	3.903	4.173	10922.1	8002.4	14907.1
Strain	150	3.951	8929.6	3.901	3.764	4.038	7960.8	5801.5	10923.7
Strain	167	4.141	13843.4	4.113	3.975	4.251	12976.4	9448.0	17822.5
Strain	126	4.274	18795.0	4.259	4.123	4.394	18136.8	13285.0	24760.6

Strain	22-2	4.342	21994.3	4.269	4.120	4.418	18580.4	13180.9	26191.6
Strain	10-2	4.382	24110.7	4.363	4.225	4.500	23041.9	16801.2	31600.8
Strain	54	4.346	22162.0	4.279	4.128	4.430	19010.0	13414.6	26939.2
Strain	198	4.790	61632.0	4.790	4.658	4.922	61645.9	45500.1	83521.1
Strain	174	4.827	67141.7	4.833	4.667	4.999	68086.7	46477.6	99742.5
Strain	178-1	4.730	53671.0	4.729	4.598	4.860	53600.9	39619.3	72516.6
Sex	Male	4.344	22083.3	4.321	4.262	4.380	20936.3	18265.1	23998.2
Sex	Female	4.364	23105.4	4.308	4.244	4.372	20317.1	17535.0	23540.7
Inf	Inf 1	4.468	29370.4	4.472	4.413	4.530	29631.2	25896.7	33904.4
Inf	Inf 2	4.293	19617.5	4.269	4.205	4.333	18580.3	16028.6	21538.3
Inf	Inf 3	4.269	18578.1	4.202	4.132	4.272	15934.6	13566.0	18716.7
Block	A	4.337	21702.8	4.286	4.225	4.347	19319.7	16790.1	22230.3
Block	B	4.370	23444.7	4.343	4.281	4.405	22017.2	19099.0	25381.4
Sex:Inf	Male & Inf 1	4.424	26525.1	4.419	4.338	4.501	26270.5	21774.6	31694.7
Sex:Inf	Male & Inf 2	4.316	20693.1	4.291	4.204	4.377	19525.2	16004.5	23820.5
Sex:Inf	Male & Inf 3	4.280	19075.1	4.253	4.164	4.341	17891.0	14582.1	21950.8
Sex:Inf	Female & Inf 1	4.513	32587.7	4.524	4.441	4.607	33421.9	27592.6	40482.7
Sex:Inf	Female & Inf 2	4.266	18449.4	4.248	4.154	4.341	17681.1	14268.1	21910.6
Sex:Inf	Female & Inf 3	4.254	17937.3	4.152	4.048	4.256	14192.1	11163.7	18041.9
Strain:Inf	Strain 66 & Inf 1	3.908	8083.4	3.912	3.699	4.124	8157.9	5006.1	13294.2
Strain:Inf	Strain 66 & Inf 2	3.748	5600.5	3.716	3.449	3.983	5194.5	2808.8	9606.7
Strain:Inf	Strain 66 & Inf 3	4.054	11331.2	4.026	3.693	4.360	10627.8	4935.1	22887.1
Strain:Inf	Strain 57 & Inf 1	4.147	14015.1	4.147	3.959	4.335	14033.0	9106.7	21624.3
Strain:Inf	Strain 57 & Inf 2	3.953	8964.9	3.960	3.768	4.152	9116.1	5860.7	14179.8
Strain:Inf	Strain 57 & Inf 3	4.016	10378.1	4.008	3.805	4.211	10185.0	6379.5	16260.4
Strain:Inf	Strain 150 & Inf 1	4.247	17669.5	4.245	4.060	4.430	17575.2	11478.6	26909.9
Strain:Inf	Strain 150 & Inf 2	3.755	5687.4	3.733	3.540	3.926	5411.8	3470.0	8440.3
Strain:Inf	Strain 150 & Inf 3	3.778	5998.2	3.725	3.508	3.941	5304.2	3220.9	8735.1
Strain:Inf	Strain 167 & Inf 1	4.356	22724.6	4.351	4.166	4.537	22449.8	14652.0	34397.7
Strain:Inf	Strain 167 & Inf 2	4.062	11541.3	4.054	3.846	4.262	11321.8	7016.3	18269.2
Strain:Inf	Strain 167 & Inf 3	3.950	8911.9	3.934	3.730	4.139	8596.7	5369.1	13764.6
Strain:Inf	Strain 126 & Inf 1	4.425	26617.8	4.433	4.244	4.622	27124.0	17556.0	41906.4
Strain:Inf	Strain 126 & Inf 2	4.352	22472.0	4.352	4.160	4.544	22487.6	14450.7	34994.2
Strain:Inf	Strain 126 & Inf 3	4.005	10126.0	3.990	3.788	4.193	9781.1	6133.5	15598.1
Strain:Inf	Strain 22-2 & Inf 1	4.576	37690.0	4.576	4.392	4.760	37690.0	24666.1	57590.7
Strain:Inf	Strain 22-2 & Inf 2	4.153	14214.6	4.150	3.942	4.358	14125.7	8748.7	22807.5
Strain:Inf	Strain 22-2 & Inf 3	4.138	13754.8	4.081	3.815	4.347	12048.3	6527.3	22239.1
Strain:Inf	Strain 10-2 & Inf 1	4.572	37294.8	4.572	4.386	4.758	37306.2	24316.2	57235.6
Strain:Inf	Strain 10-2 & Inf 2	4.227	16868.5	4.219	4.016	4.421	16556.3	10386.8	26390.1
Strain:Inf	Strain 10-2 & Inf 3	4.299	19898.9	4.297	4.090	4.503	19806.7	12314.8	31856.3

Strain:Inf	Strain 54 & Inf 1	4.566	36841.7	4.568	4.378	4.758	36982.2	23889.2	57251.2
Strain:Inf	Strain 54 & Inf 2	4.342	21953.8	4.333	4.088	4.577	21506.9	12237.5	37797.4
Strain:Inf	Strain 54 & Inf 3	3.941	8739.1	3.936	3.692	4.181	8637.2	4918.1	15168.8
Strain:Inf	Strain 198 & Inf 1	4.725	53032.8	4.726	4.540	4.912	53232.7	34695.1	81675.0
Strain:Inf	Strain 198 & Inf 2	4.821	66169.0	4.817	4.626	5.008	65664.9	42314.5	101900.6
Strain:Inf	Strain 198 & Inf 3	4.826	67018.9	4.826	4.640	5.012	67019.5	43677.0	102836.8
Strain:Inf	Strain 174 & Inf 1	4.906	80629.1	4.924	4.689	5.159	83907.0	48819.6	144212.1
Strain:Inf	Strain 174 & Inf 2	4.929	84823.0	4.930	4.695	5.165	85148.7	49534.1	146369.6
Strain:Inf	Strain 174 & Inf 3	4.650	44626.5	4.645	4.412	4.879	44178.4	25807.7	75626.1
Strain:Inf	Strain 178-1 & Inf 1	4.737	54541.9	4.735	4.548	4.922	54334.2	35335.2	83548.5
Strain:Inf	Strain 178-1 & Inf 2	4.697	49820.3	4.696	4.510	4.882	49655.4	32363.3	76187.0
Strain:Inf	Strain 178-1 & Inf 3	4.755	56907.9	4.756	4.571	4.942	57078.9	37201.5	87577.1

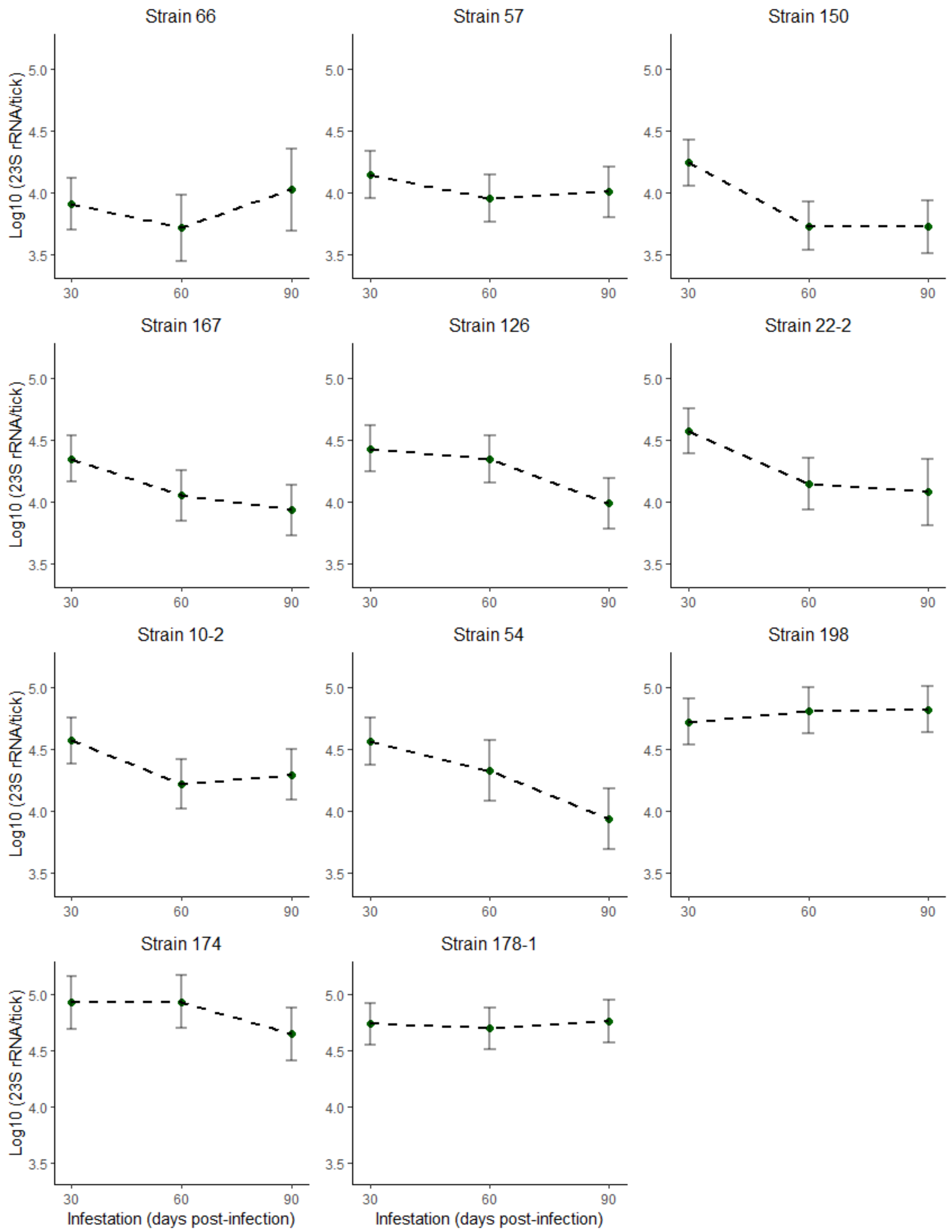

Figure S3. The nymphal spirochete load (NSL) of *B. burgdorferi* from infected mice to immature *I. scapularis* ticks decreased over the 3 successive larval infestations for some strains but remained constant or increased over time for other strains. For this reason, the 2-way interaction between strain and infestation was significant ($p = 7.561 \times 10^{-5}$). NSL was measured by infesting mice with *I. scapularis* larvae on 3 occasions (days 30, 60, and 90 post-infection), allowing the engorged larvae to moult into nymphs, and testing the nymphs for infection with *B. burgdorferi* and measuring the abundance of the *B. burgdorferi 23S rRNA* gene in each tick. The mean values (green circles) for each infestation are the EMMs from the LMM for the NSL of each strain over time. Error bars are the 95% confidence intervals for the EMMs.

Section 8 – Relationship between the mouse tissue spirochete loads and NIP

Statistical Methods: We wanted to determine which mouse tissues were most strongly associated with variation in the NIP. There were 8 mouse tissues: ear biopsies that were sampled the day before the larval infestation (i.e., days 29, 59, and 89 PI) and 7 necropsies that were sampled at the end of the study (day 97 PI). Each mouse tissue had an estimate of infection prevalence (0 = uninfected, 1 = infected) and an estimate of spirochete load, which was calculated as log10[(*23S rRNA* gene copies/10^6 mouse *Beta-actin* copies) + 1]. The advantage of this log[X + 1] transformation is that uninfected tissues are defined and have a value of 0. The mouse tissue spirochete loads were scaled to z-scores with mean of 0 and a variance of 1 (hereafter referred to as the z-score spirochete loads). We created 4 additional composite variables: (1) mean proportion of infected necropsies, (2) z-score spirochete load averaged over all necropsies, (3) z-score spirochete load averaged over the three skin-related tissues (ventral skin, left ear, right ear), (4) z-score spirochete load averaged over two internal organs (bladder, heart).

The 20 different mouse tissue variables are highly correlated, and a multiple regression approach was therefore not feasible. For this reason, we used AIC-based model selection to compare 20 different models; each model was a GLMM with binomial errors that analysed the NIP as a function of a different mouse tissue variable. Each model included the fixed factors of mouse sex, infestation, and temporal block, and all interactions between the mouse tissue variable, mouse sex, and infestation. As variation in NIP among strains was low in infestation 1, we expected that spirochete load (or infection prevalence) would explain more variation in NIP for infestations 2 and 3 (i.e., we expect interactions between mouse tissue variable and infestation). Mouse identity was included as a random factor. Models were ranked according to their corrected Akaike Information Criterion (AICc) where the best models have the lowest AICc scores. The difference in AICc from the top model was used to calculate the weight or support for each model. If a model has high support, it means that the mouse tissue variable is important for explaining variation in the NIP.

Results: The top 5 GLMMs of the NIP had 99.2% of the support (**Table S18**). These 5 models included the following 5 mouse tissue variables: heart spirochete load (30.7% support), bladder spirochete load (29.7% support), average of heart and bladder (26.7%), ear biopsy (7.5%), and heart infection prevalence (4.7%). Thus, GLMMs that included the internal organs of the heart and/or the bladder had 91.7% of the support.

GLMM of NIP versus the heart spirochete load: For the GLMM of the NIP as a function of the heart spirochete load, we simplified the model by removing non-significant terms. The simplified model contained the main effects of heart tissue spirochete load, mouse sex, infestation, and their interaction (**Table S19**). The heart spirochete load had a significant effect on the NIP ($p < 0.001$). We investigated the parameter estimates for the simplified model (**Table S20**). The slope of the relationship between the NIP and the heart spirochete load was positive and highly significant (slope = 0.751, SE = 0.164, $p < 0.001$). This result indicates that *B. burgdorferi* strains that establish higher spirochete loads in the heart tissues have higher host-to-tick transmission to feeding ticks (**Figure S4**).

For each strain, we calculated the mean heart spirochete load over the mice (up to 8 mice per strain) and the mean NIP (hereafter the lifetime NIP). The lifetime NIP for each strain was calculated by summing the number of infected nymphs and the number of tested nymphs over all the infestations and all the mice. A GLM with quasibinomial errors found a strong positive

relationship between heart spirochete load and lifetime NIP across the 11 *B. burgdorferi* strains (slope = 0.890, SE = 0.214, t = 4.152, p = 0.002).

GLMM of NIP versus the ear biopsy spirochete load: For the GLMM of the NIP as a function of the ear biopsy spirochete load, we simplified the model by removing non-significant terms. The simplified model contained the main effects of ear biopsy tissue spirochete load, mouse sex, infestation, and the 2-way interactions between ear biopsy spirochete load and infestation and between sex and infestation (**Table S21**). The significant ear biopsy spirochete load: infestation interaction (p = 0.002) indicates that the slope relating NIP to ear biopsy spirochete load differs between infestations. We investigated the parameter estimates for the simplified model (**Table S22**). The slope of the relationship between the NIP and the ear biopsy spirochete load was negative for infestation 1 (slope = -0.790, SE = 0.278, p = 0.004), whereas it was positive for infestation 2 (contrast of slopes = 1.261, SE = 0.407, p = 0.002) and for infestation 3 (contrast of slopes = 1.011, SE = 0.300, p = 0.001). This result indicates that *B. burgdorferi* strains that establish higher spirochete loads in the ear tissues have higher host-to-tick transmission to feeding ticks for infestation 2 at day 60 PI and for infestation 3 at day 90 PI (**Figure S5**).

For each strain, we calculated the mean ear biopsy spirochete load over the mice and the 3 infestations (up to 24 ear biopsies per strain; 8 mice x 3 infestations per mouse) and the mean NIP (hereafter the lifetime NIP). The lifetime NIP for each strain was calculated by summing the number of infected nymphs and the number of tested nymphs over all the infestations and all the mice. A GLM with quasibinomial errors found a strong positive relationship between ear biopsy spirochete load and lifetime NIP across the 11 *B. burgdorferi* strains (slope = 2.357, SE = 0.467, t = 5.052, p < 0.001).

Table S18. AIC-based model selection table is shown for 20 GLMMs with binomial errors that analyzed the NIP. Each model contained a different mouse tissue variable that was either an infection prevalence or a spirochete load. Each model also included the fixed factors of mouse sex, infestation, temporal block, various interactions, and the random factor of mouse ID. For each model, shown are the model rank, mouse tissue, variable type (infection prevalence or spirochete load), model degrees of freedom (df), corrected Akaike information criterion (AICc), change in the AICc value from the top model (delta), weight of model (weight1) and cumulative weight of the models (weight2).

Model Rank	Mouse tissue	Variable	df	AICc	delta	weight1	weight2
1	Heart	Load	14	1597.346	0.000	0.307	0.307
2	Bladder	Load	14	1597.411	0.066	0.297	0.603
3	Internal	Load	14	1597.620	0.275	0.267	0.870
4	Biopsy	Load	14	1600.161	2.815	0.075	0.945
5	Heart	Prevalence	14	1601.115	3.769	0.047	0.992
6	All necropsies	Load	14	1606.878	9.532	0.003	0.994
7	Ear right	Load	14	1608.088	10.742	0.001	0.996
8	Ventral	Load	14	1608.495	11.150	0.001	0.997
9	Biopsy	Prevalence	12	1608.939	11.593	0.001	0.998
10	Joint	Load	14	1609.349	12.003	0.001	0.999

11	Ventral	Prevalence	14	1610.122	12.777	0.001	0.999
12	Joint	Prevalence	14	1611.939	14.593	0.000	0.999
13	Ear right	Prevalence	14	1612.140	14.794	0.000	1.000
14	Ear left	Prevalence	14	1613.353	16.007	0.000	1.000
15	Bladder	Prevalence	11	1613.904	16.559	0.000	1.000
16	Skin	Load	14	1613.966	16.620	0.000	1.000
17	Ear left	Load	14	1614.051	16.705	0.000	1.000
18	Kidney	Prevalence	14	1616.168	18.822	0.000	1.000
19	Kidney	Load	14	1616.339	18.994	0.000	1.000
20	All necropsies	Prevalence	14	1617.072	19.727	0.000	1.000

Table S19. Model simplification of the GLMM of the NIP as a function of heart spirochete load. Non-significant interactions were removed from the model. The final simplified model contained the covariate of heart spirochete load, the fixed factors of sex, infestation, and their interaction. For each term, the Chi-squared statistic (Chi), degrees of freedom (df), and p-value (p) are shown.

Term	Order removed	Chi	df	p	Signif [a]
Heart spiro load	Final model	21.098	1	< 0.001	***
Sex	Final model	4.705	1	0.030	*
Infestation	Final model	111.507	2	< 2.2e-16	***
Sex:Infestation	Final model	17.322	2	< 0.001	***

[a] Categories of significance are as follows: * 0.05 < p > 0.01, ** 0.01 < p > 0.001, *** p < 0.001.

Table S20. Parameter estimates of the GLMM of the NIP as a function of heart spirochete load. Explanatory variables include heart spirochete load, mouse sex (2 levels: male, female), infestation (3 levels: infestation 1, infestation 2, infestation 3), and their interaction. For each parameter in the GLMM, the type, description, estimate, standard error (SE), and p-value (p) are shown.

Parameter type	Description	Estimate	SE	p	Signif [a]
Intercept	Reference [b]	4.824	0.428	< 0.001	***
Slope	Heart spirochete load	0.751	0.164	< 0.001	***
Contrast	Male - Female	-0.917	0.540	0.090	
Contrast	Inf 2 - Inf 1	-3.061	0.381	< 0.001	***
Contrast	Inf 3 - Inf 1	-3.688	0.383	< 0.001	***
Interaction	Male:Infestation 2	1.642	0.479	0.001	***
Interaction	Male:Infestation 3	1.987	0.478	< 0.001	***

[a] Categories of significance are as follows: * 0.05 < p > 0.01, ** 0.01 < p > 0.001, *** p < 0.001.

[b] Reference condition refers to the infection status of unfed nymphs from infestation 1 that fed as larvae on a female mouse for which the heart tissue spirochete load = 0 (which is the mean).

Table S21. Model simplification of the GLMM of the NIP as a function of ear biopsy spirochete load. Non-significant interactions were removed from the model. The final simplified model contained the covariate of ear biopsy spirochete load, the fixed factors of sex, infestation, and the ear biopsy spirochete load:infestation interaction and the sex:infestation interaction. For each term, the Chi-squared statistic (Chi), degrees of freedom (df), and p-value (p) are shown.

Term	Order removed	Chi	df	p	Signif [a]
Ear biopsy spiro load	Final model	1.4840	1	0.223	
Sex	Final model	3.8984	1	0.048	*
Infestation	Final model	60.7209	2	< 0.001	***
Ear biopsy spiro load:Inf	Final model	12.4535	2	0.002	**
Sex:Infestation	Final model	20.4186	2	< 0.001	***

[a] Categories of significance are as follows: * 0.05 < p > 0.01, ** 0.01 < p > 0.001, *** p < 0.001.

Table S22. Parameter estimates of the GLMM of the NIP as a function of ear biopsy spirochete load. Explanatory variables include ear biopsy spirochete load, mouse sex (2 levels: male, female), infestation (3 levels: infestation 1, infestation 2, infestation 3), and their interactions. For each parameter in the GLMM, the type, description, estimate, standard error (SE), and p-value (p) are shown.

Parameter type	Description	Estimate	SE	p	Signif [a]
Intercept	Reference [b]	5.379	0.527	< 0.001	***
Slope	Ear biopsy spiro load	-0.790	0.278	0.004	**
Contrast	Male - Female	-1.050	0.558	0.060	
Contrast	Inf 2 - Inf 1	-3.490	0.505	< 0.001	***
Contrast	Inf 3 - Inf 1	-4.190	0.483	< 0.001	***
Interaction	Ear biopsy spiro load:Inf 2	1.261	0.407	0.002	**
Interaction	Ear biopsy spiro load:Inf 3	1.011	0.300	0.001	**
Interaction	Male:Inf 2	1.681	0.496	0.001	**
Interaction	Male:Inf 3	2.174	0.483	< 0.001	***

[a] Categories of significance are as follows: * 0.05 < p > 0.01, ** 0.01 < p > 0.001, *** p < 0.001.

[b] Reference condition refers to the infection status of unfed nymphs from infestation 1 that fed as larvae on a female mouse for which the ear biopsy tissue spirochete load = 0 (which is the mean).

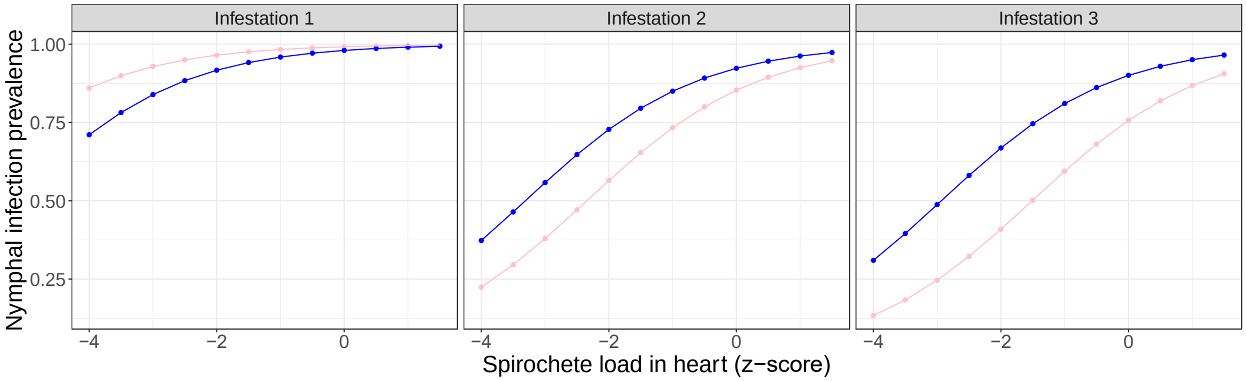

Figure S4. GLMM predicts a positive relationship between NIP and heart spirochete load. Larval infestations 1, 2, and 3 are shown in separate panels. Female and male mice are shown with pink and blue lines, respectively. The original heart spirochete loads were calculated as log10[(*23S rRNA* copies + 1)/10^6 *Beta-actin* copies] and these were converted to z-scores with a mean of 0 and a variance of 1.

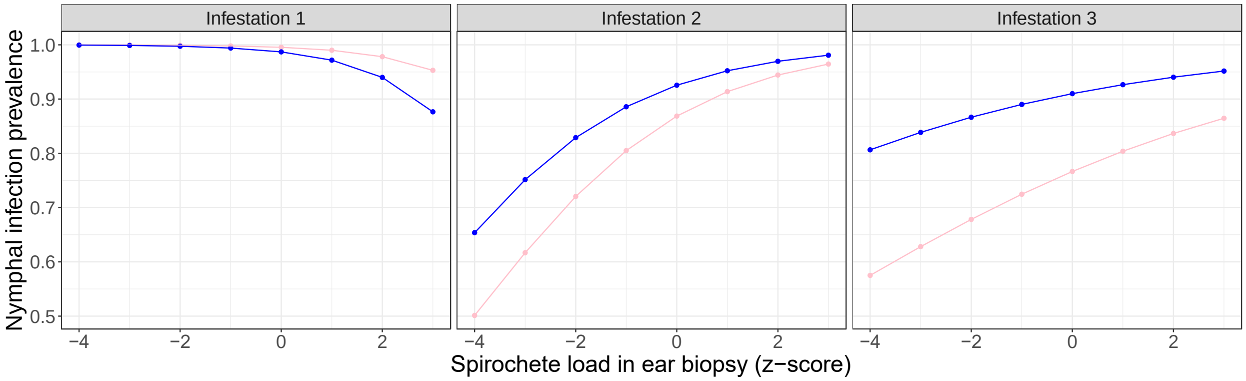

Figure S5. GLMM predicts a negative relationship between NIP and ear biopsy spirochete load for infestation 1 and a positive relationship for infestations 2 and 3. Larval infestations 1, 2, and 3 are shown in separate panels. Female and male mice are shown with pink and blue lines, respectively. The original ear biopsy spirochete loads were calculated as log10[(*23S rRNA* copies + 1)/10^6 *Beta-actin* copies] and these were converted to z-scores with a mean of 0 and a variance of 1.

Section 9 – Correlation matrix of mouse tissue spirochete loads and *B. burgdorferi* transmission variables

The pairwise correlation matrix was calculated for 12 variables: eight estimates of tissue spirochete load (1) ear biopsy, (2) ventral skin, (3) left ear, (4) right ear, (5) heart, (6) bladder, (7) kidney, (8) joint, and four estimates of host-to-tick transmission: (9) larval infection prevalence

(LIP), (10) larval spirochete load (LSL), (11) nymphal infection prevalence (NIP), and (12) nymphal spirochete load (NSL). The phenotypic correlation matrix was calculated across the 84 mice, whereas the genetic correlation matrix was calculated across the 11 *B. burgdorferi* strains. The spirochete loads for the mouse tissues and the ticks were log10[X + 1]-transformed where X is the ratio of *23S rRNA* gene copies per 10^6 *Beta-actin* gene copies.

For the genetic correlation matrix, the four estimates of host-to-tick transmission were all positively correlated with each other (**Figure S6**). Spirochete loads of the ear biopsies, heart, bladder, and kidney were positively correlated with the NIP and the NSL. Spirochete loads of the ear biopsies, heart, and bladder were positively correlated with the LIP and the LSL. The results for the phenotypic correlation matrix were very similar (**Figure S7**).

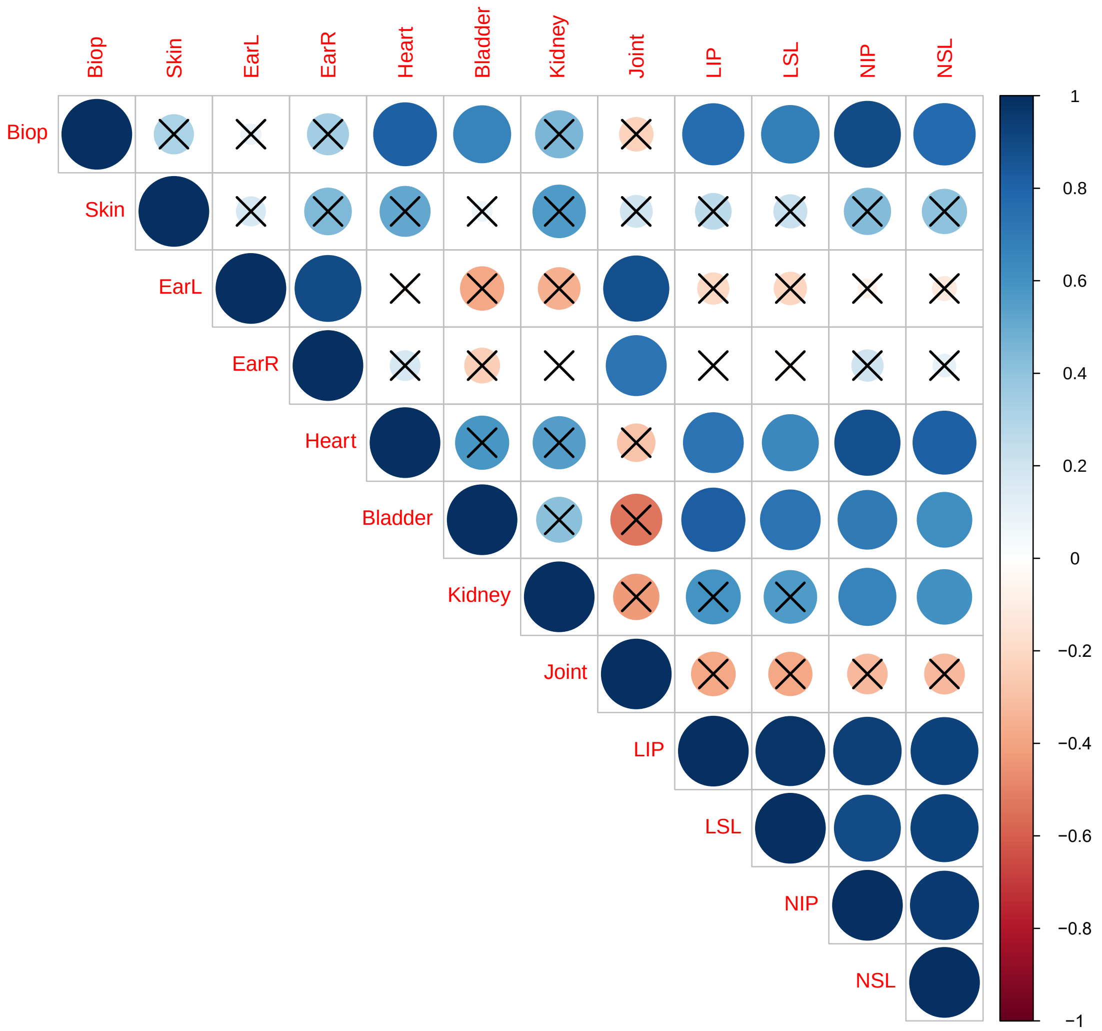

Figure S6. Genetic correlation matrix for the 12 variables across the 11 strains of *B. burgdorferi*. The 12 variables include spirochete loads in 8 mouse tissues: (1) ear biopsy, (2) ventral skin, (3) left ear, (4) right ear, (5) heart, (6) bladder, (7) kidney, (8) joint, and four estimates of host-to-tick transmission of *B. burgdorferi*: (9) larval infection prevalence (LIP), (10) larval spirochete load (LSL), (11) nymphal infection prevalence (NIP), and (12) nymphal spirochete load (NSL). The size and color of the circle indicate the absolute value and direction of the Pearson correlation coefficient. Non-significant pairwise correlations are indicated with an 'X'.

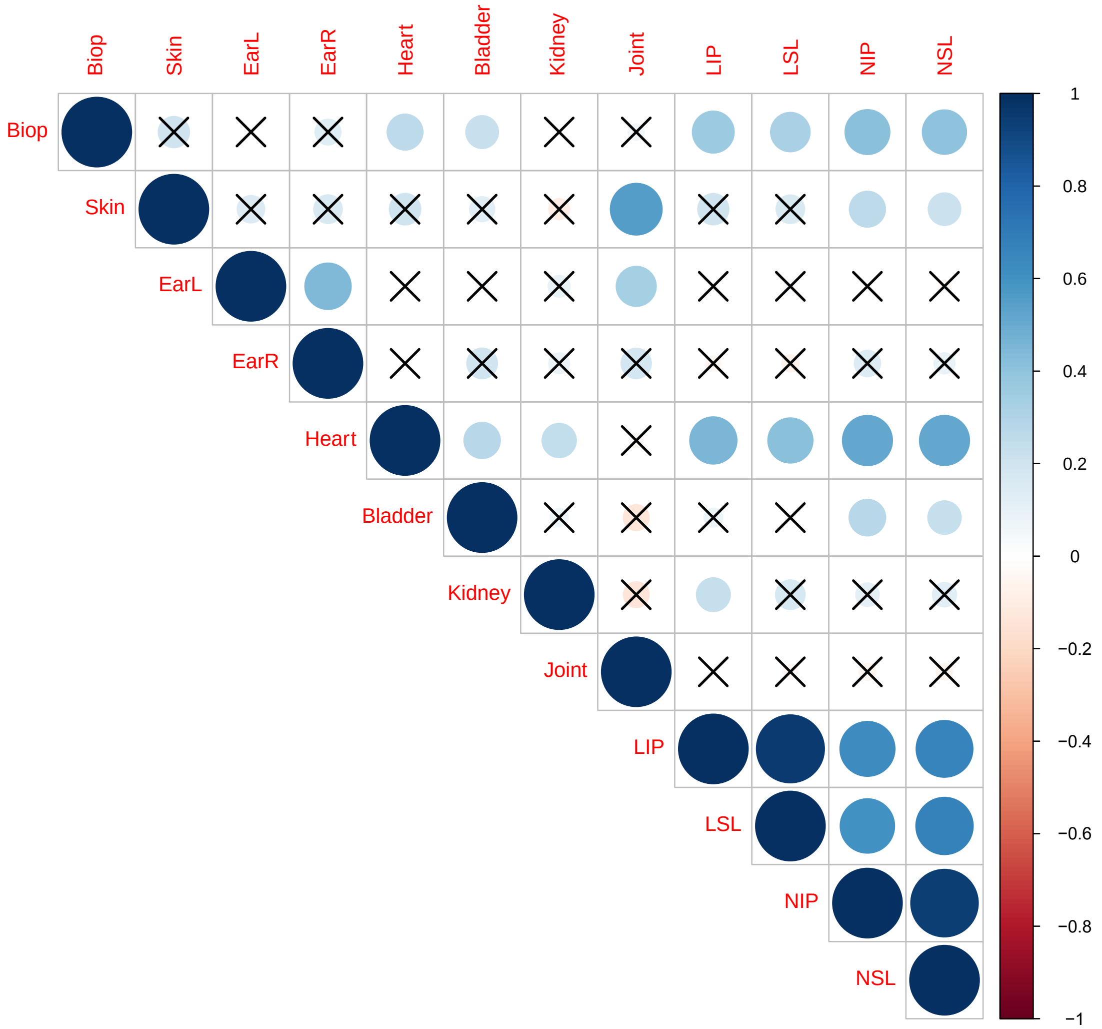

Figure S7. Phenotypic correlation matrix for the 12 variables across the 84 infected mice. The 12 variables include spirochete loads in 8 mouse tissues: (1) ear biopsy, (2) ventral skin, (3) left ear, (4) right ear, (5) heart, (6) bladder, (7) kidney, (8) joint, and four estimates of host-to-tick transmission of *B. burgdorferi*: (9) larval infection prevalence (LIP), (10) larval spirochete load

(LSL), (11) nymphal infection prevalence (NIP), and (12) nymphal spirochete load (NSL). The size and color of the circle indicate the absolute value and direction of the Pearson correlation coefficient. Non-significant pairwise correlations are indicated with an 'X'.

Section 10 – Estimation of MLST frequencies in *I. scapularis* ticks in North America.

Frequency of *B. burgdorferi* strains in *I. scapularis* ticks in North America: Two previous studies determined the frequencies of *B. burgdorferi* MLSTs in questing *I. scapularis* ticks collected in the USA and Canada [19, 20]. The number of *I. scapularis* ticks infected with each MLST are reported in Table 1 in Travinsky et al (2010) and Table 2 in Ogden et al (2011). After removing the MLSTs and ticks from northern California and western Europe, the study by Travinsky et al (2010) identified 35 different MLSTs in 654 *I. scapularis* ticks. The study by Ogden et al (2011) identified 40 different MLSTs in 166 *I. scapularis* ticks. These two studies combined contained 9 of the 11 strains and 8 of the 10 unique MLSTs in the present study. Strains Bb16-66 and Bb16-54, which corresponded to MLSTs 237 and 741, were not detected, and were therefore assigned counts of zero. For each study, we counted the number of *I. scapularis* ticks infected with each of the 10 unique MLSTs of interest. To obtain the best estimate of the strain-specific frequencies in *I. scapularis* populations, the counts were combined for the two studies. These counts were then converted to the strain-specific frequencies.

Study by Travinsky et al (2010): In Travinsky et al (2010), the MLST, *ospC* type, geographic region, and the number of ticks infected with a particular MLST and *ospC* type are given in columns 8, 1, 3, and 5 (denominator) of Table 1. For each of our 10 unique MLSTs, we searched for a matching MLST (without regard to *ospC* type) in Table 1 of Travinsky et al (2010). Of the 11 strains, 9 strains (highlighted in green in **Table S23**) had a matching MLST and 2 strains did not (Bb16-54 and Bb16-66; highlighted in blue in **Table S23**). These two 'rare' strains were assigned a count of 0 in **Table S23**. The number of *I. scapularis* ticks in which each MLST was found (N) is given in **Table S23**.

We wanted to obtain an estimate of the sampling effort for the northeastern USA and the midwestern USA (referred to as north-central USA in Travinsky et al (2010)). We removed all strains from northern California and Europe (n = 123 ticks), which left us with 654 ticks, of which 254, 257, and 143 ticks occurred in the northeastern USA, midwestern USA, or both regions, respectively. The 143 ticks that carry the MLSTs that occur in both regions were split among each region; the sampling effort was very similar between the northeastern USA (n = 254 + 72 = 326 ticks) and the midwestern USA (n = 257 + 71 = 328 ticks).

Of the 9 strains with a matching MLST, 7 strains (highlighted in green in **Table S23**) had a matching *ospC* type and 2 strains did not (Bb16-10-2 and Bb16-57-1; highlighted in pink in **Table S23**). There was also a match in geographic region between the present study and Travinsky et al (2010). All 5 strains that were obtained from Nova Scotia (East Canada) occurred in the northeastern United States (Region 1) in Travinsky et al (2010). Of the 6 strains that were sampled from Manitoba and western Ontario (Midwest Canada), 4 occurred in the north-central United States (Region 2) in Travinsky et al (2010).

Table S23. The 11 *B. burgdorferi* strains in our study were matched via their MLST to the number of *I. scapularis* ticks carrying that strain in the USA in the study by Travinsky et al (2010). For each of the 11 strains, the NML strain ID, region in Canada from which the strain was sampled, MLST, and *ospC* type are shown. With respect to the study by Travinksy et al

(2010), the MLST, *ospC* type, geographic region, and the number of *I. scapularis* ticks in which that combination of MLST and *ospC* type was found (N) are shown.

	Present study			Travinsky et al (2010)			
Strain ID	Region	MLST	*ospC* type	MLST	*ospC*	Region *	N
10-2	Midwest	32	H, C3	32	Hb	2	65
22-2	Midwest	55	A	55	A	2	4
54	Midwest	741	I	---	---	---	0
57-1	Midwest	29	A3	29	L	2	25
66	Midwest	237	J	---	---	---	0
126	Midwest	43	N	43	N	2	10
	Midwest			Sum			104
	Midwest			Other			224
	Midwest			Total			328
150	East	19	E	19	E	1, 2	19
167	East	12	M	12	M	1	4
174	East	37	T	37	T	1	16
178-1, 198	East	3	K	3	K	1	68
	East			Sum			107
	East			Other			219
	East			Total			326
	Both			Sum			211
	Both			Other			443
	Both			Total			654

* In Travinsky et al (2010), regions 1 and 2 refer to the northeastern United States and the north-central United States, respectively.

Study by Ogden et al (2011): In Ogden et al (2011), the MLST, count, and geographic region are given in columns 1, 2, and 3 in Table 2. For each of our 10 unique MLSTs, we searched for a matching MLST in Table 2 in Ogden et al (2011). Of the 11 strains, 7 strains (highlighted in green in **Table S24**) had a matching MLST and 4 strains did not (Bb16-10-2, Bb16-54, Bb16-66, and Bb16-126; highlighted in blue in **Table S24**). These four 'rare' strains were assigned a count of 0 in **Table S24**. The number of *I. scapularis* ticks in which each MLST was found (N) is given in **Table S24**.

In Ogden et al (2011), the MLSTs in the 166 ticks were classified according to whether they had already been identified in northeastern USA (n = 150 ticks), midwestern USA (n = 4 ticks), or both regions (n = 12 ticks). The 12 ticks that carry the MLSTs that occur in both regions were split among each region; the sampling effort was much higher in the northeastern USA (n = 150 + 6 = 156 ticks) compared to the midwestern USA (n = 4 + 6 = 10 ticks).

Table S24. The 11 *B. burgdorferi* strains in our study were matched via their MLST to the number of *I. scapularis* ticks carrying that strain in Canada in the study by Ogden et al (2011). For each of the 11 strains, the NML strain ID, region in Canada from which the strain was

sampled, MLST, and *ospC* type are shown. With respect to the study by Ogden et al (2011), the MLST, geographic region, and the number of *I. scapularis* ticks in which that MLST was found (N) are shown.

	Present study			Ogden et al. (2011)		
Strain ID	Region	MLST	*ospC* type	MLST	Region *	N
10-2	Midwest	32	H, C3	--	--	0
22-2	Midwest	55	A	55	MW	1
54	Midwest	741	I	--	--	0
57-1	Midwest	29	A3	29	NE + MW	4
66	Midwest	237	J	--	--	0
126	Midwest	43	N	--	--	0
	Midwest			Sum		5
	Midwest			Other		5
	Midwest			Total		10
150	East	19	E	19	NE	6
167	East	12	M	12	NE + MW	3
174	East	37	T	37	NE	2
178-1, 198	East	3	K	3	NE	26
	East			Sum		37
	East			Other		119
	East			Total		156
	Both			Sum		42
	Both			Other		124
	Both			Total		166

* In Ogden et al (2011), NE and MW refer to the fact that these MLSTs had already been identified in the northeastern United States and the midwestern United States, respectively.

Estimation of the MLST-specific frequencies: Our study contained 10 unique MLSTs because strains 178-1 and 198 both had MLST 3. For each MLST, we summed the counts for the studies by Travinsky et al (2010) and by Ogden et al (2011). The total number of *I. scapularis* ticks in which these 10 MLSTs were detected (N1) was 253 ticks. The total number of *I. scapularis* ticks sampled from the northeastern and midwestern USA for the two studies combined (N2) was 820 ticks. When N1 is split among the two regions (N3), there are 144 and 109 ticks in the northeastern and midwestern USA, respectively. When N2 is split among the two regions (N4), there are 482 and 338 ticks in the northeastern and midwestern USA, respectively.

The calculation of the strain-specific frequencies is shown in **Table S25**. Frequency 1 (%) of each MLST was calculated by dividing the strain-specific count by N1 (n = 253) and multiplying by 100. For example, frequency 1 for MLST 3 is 100*(94/253) = 37.15%. Frequency 2 (%) of each MLST was calculated by dividing the strain-specific count by N2 (n = 820) and multiplying by 100. For example, frequency 2 for MLST 3 is 100*(94/820) = 11.46%. Frequency 3 (%) of each MLST was calculated by dividing the strain-specific count by N3 (n = 109 for midwestern USA; n = 144 for northeastern USA) and multiplying by 100. For example,

frequency 3 for MLST 3 is 100*(94/144) = 65.28%. Frequency 4 (%) of each MLST was calculated by dividing the strain-specific count by N4 (n = 338 for midwestern USA; n = 482 for northeastern USA) and multiplying by 100. For example, frequency 4 for MLST 3 is 100*(94/482) = 19.50%. Frequencies 1 and 3 are calculated over the subset of the 10 MLSTs of interest, whereas frequencies 2 and 4 are calculated over all the MLSTs in the two regions of interest. Frequencies 1 and 2 use a common denominator, which does not account for potential differences in sampling effort between regions, whereas frequencies 3 and 4 account for potential differences in sampling effort between regions.

Table S25. Estimation of the frequencies of the 11 strains of *B. burgdorferi* in *I. scapularis* ticks in North America. The counts (number of ticks infected with the MLST of interest) for the 10 unique MLSTs and the other MLSTs are shown separately for the study by Travinsky et al (2010) and by Ogden et al (2011). Other MLSTs that belonged to both regions were split among the two regions as explained above. The counts were summed across the two studies (Sum). Frequencies 1, 2, 3, and 4 were calculated using 4 different denominators: N1, N2, N3, and N4.

Strain ID	Region	MLST	Travinsky (2010)	Ogden (2011)	Sum	N1	N2	N3	N4	Freq1 (%)	Freq2 (%)	Freq3 (%)	Freq4 (%)
10-2	Midwest	32	65	0	65	253	820	109	338	25.692	7.927	59.633	19.231
22-2	Midwest	55	4	1	5	253	820	109	338	1.976	0.610	4.587	1.479
54	Midwest	741	0	0	0	253	820	109	338	0.000	0.000	0.000	0.000
57-1	Midwest	29	25	4	29	253	820	109	338	11.462	3.537	26.606	8.580
66	Midwest	237	0	0	0	253	820	109	338	0.000	0.000	0.000	0.000
126	Midwest	43	10	0	10	253	820	109	338	3.953	1.220	9.174	2.959
	Midwest	Sum	104	5	109					43.083	13.293	100.000	32.249
	Midwest	Other	224	5	229							0.000	67.751
	Midwest	Total	328	10	338							100.000	100.00
150	East	19	19	6	25	253	820	144	482	9.881	3.049	17.361	5.187
167	East	12	4	3	7	253	820	144	482	2.767	0.854	4.861	1.452
174	East	37	16	2	18	253	820	144	482	7.115	2.195	12.500	3.734
178-1, 198	East	3	68	26	94	253	820	144	482	37.154	11.463	65.278	19.502
	East	Sum	107	37	144					56.917	17.561	100.000	29.876
	East	Other	219	119	338							0.000	70.124
	East	Total	326	156	482							100.000	100.000
	Both	Sum	211	42	253					100.000	30.854		
	Both	Other	443	124	567					0.000	69.146		
	Both	Total	654	166	820					100.000	100.000		

Section 11 – Relationship between laboratory estimates of lifetime NIP and frequencies observed in *I. scapularis* ticks across 11 *B. burgdorferi* strains.

We tested whether there was a relationship between the lifetime NIP and the MLST frequencies observed in *I. scapularis* ticks in North America across the 11 *B. burgdorferi* strains. The lifetime NIP was calculated by summing the infected and uninfected nymphs over all infestations and mice for each of the 11 *B. burgdorferi* strains. We analyzed the frequencies that included the other MLSTs when the two regions were combined (i.e., MLST frequency 2 in **Table S25**), and when the two regions were adjusted for sampling effort (i.e., MLST frequency 4 in **Table S25**). MLST frequency was analysed using a generalized linear model (GLM) with quasibinomial errors to correct for overdispersion. Explanatory variables included the fixed factor geographic region (2 levels: northeastern USA and midwestern USA), the covariate of the strain-specific lifetime NIP, and their interaction. The model was simplified by sequential removal of the least significant terms.

For MLST frequency 2, the interaction between geographic region and lifetime NIP was not significant and was removed from the model (Chi-square = 0.002, df = 1, p = 0.962). Geographic region did not have a significant effect on MLST frequency 2 and was removed from the model (Chi-square = 0.001, df = 1, p = 0.970). Lifetime NIP had a significant effect on MLST frequency 2 (Chi-square = 10.771, df = 1, p = 0.001). The slope of the relationship between NIP and MLST frequency 2 was positive (**Figure S7**; slope = 9.685, SE = 3.724, t = 2.601, p = 0.029). If strain Bb16-10-2 is removed from the analysis (due to a suspected mixed infection), the relationship becomes more statistically significant (slope = 12.778, SE = 4.039, t = 3.164, p = 0.013). Strains with higher lifetime host-to-tick transmission had a higher frequency in *I. scapularis* populations in nature.

For MLST frequency 4, the interaction between geographic region and lifetime NIP was not significant and was removed from the model (Chi-square = 0.002, df = 1, p = 0.968). Geographic region did not have a significant effect on MLST frequency 4 and was removed from the model (Chi-square = 0.308, df = 1, p = 0.578). Lifetime NIP had a significant effect on MLST frequency 4 (Chi-square = 7.407, df = 1, p = 0.006). The slope of the relationship between NIP and MLST frequency 4 was positive (**Figure S8**; slope = 8.708, SE = 3.942, t = 2.209, p = 0.0545). If strain Bb16-10-2 is removed from the analysis (due to a suspected mixed infection), the relationship becomes more statistically significant (slope = 11.851, SE = 3.860, t = 3.070, p = 0.015). After adjusting for differential sampling effort between the two geographic regions, strains with higher lifetime host-to-tick transmission had a higher frequency in *I. scapularis* populations in nature.

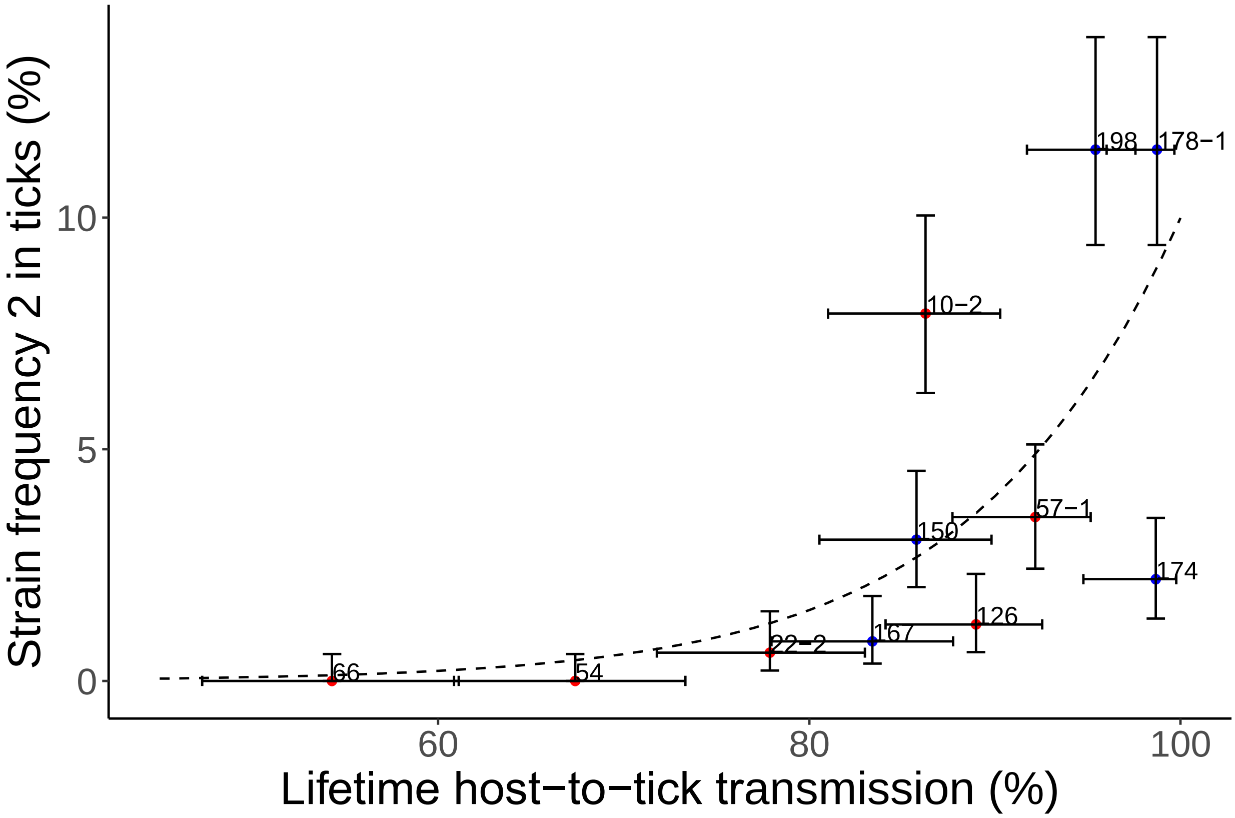

Figure S7. Our laboratory-based estimates of the lifetime host-to-tick transmission for the 11 strains of *B. burgdorferi* are positively related to the frequencies of these strains in natural populations of *I. scapularis* ticks in Canada and the USA. The strain-specific frequencies were combined both the northeastern and midwestern USA and did not adjust for differential sampling effort between regions (i.e., frequency 2 in **Table S25**). The slope of the logistic regression is positive and significant (slope = 9.685, SE = 3.724, t = 2.601, p = 0.029). The error bars represent the 95% confidence intervals.

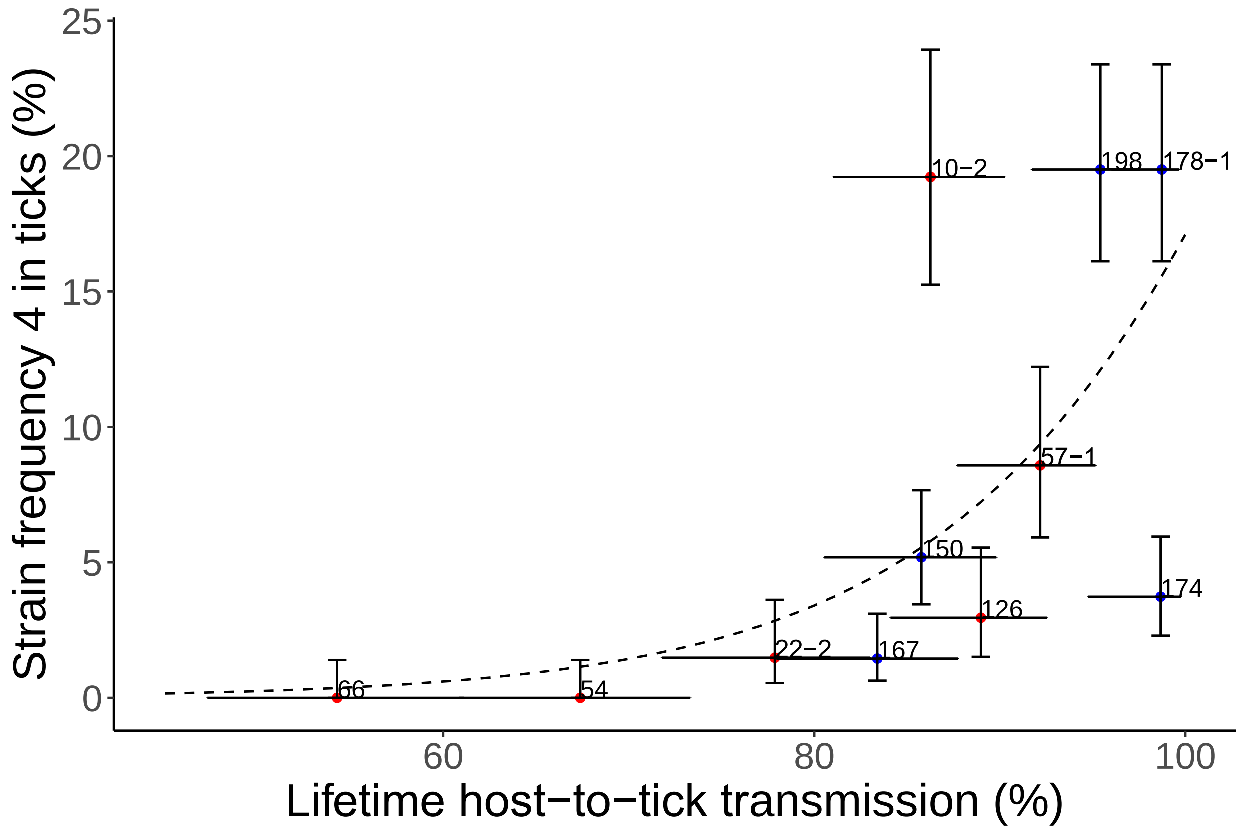

Figure S8. Our laboratory-based estimates of the lifetime host-to-tick transmission for the 11 strains of *B. burgdorferi* are positively related to the frequencies of these strains in natural populations of *I. scapularis* ticks in Canada and the USA. The strain-specific frequencies were calculated separately for the northeastern and midwestern USA and therefore adjusted for differential sampling effort between regions (i.e., frequency 4 in **Table S25**). The slope of the logistic regression is positive and just above the threshold of statistical significance (slope = 8.708, SE = 3.942, t = 2.209, p = 0.055). The error bars represent the 95% confidence intervals.

Table S26. Infection status of the ear biopsies and necropsies for the 11 strains of *B. burgdorferi*. For each strain, the NML strain ID, geographic region from which the strain was sampled, multi-locus sequence type (MTSL), number of infected mice, number of infected ear biopsies per total number of ear biopsies tested, and the percentage of infected ear biopsies are shown. The 7 necropsy tissues include the ventral skin, left ear, right ear, heart, bladder, kidney, and rear tibiotarsal joint. For each strain and necropsy type, the number of mice for which the necropsy was infected with that strain are shown.

Strain	Region	MLST	Mice	Infected/ Total Biopsies	Biopsy (%)	Ventral	Ear left	Ear right	Heart	Bladder	Kidney	Joint
10-2	Midwest	32	8	24/24	100.0	4	2	0	8	8	2	5
22-2	Midwest	55	8	23/23	100.0	4	6	2	8	8	1	8
54	Midwest	741	8	22/24	91.7	4	5	1	6	7	0	7
57-1	Midwest	29	8	23/23	100.0	3	5	3	8	8	2	8
66	Midwest	237	7	16/21	76.2	4	2	0	3	7	2	5
126	Midwest	43	8	24/24	100.0	7	5	4	8	8	4	8
150	East	19	8	24/24	100.0	4	1	0	8	8	4	3
167	East	12	8	24/24	100.0	4	2	0	8	8	2	5
174	East	37	5	14/15	93.3	3	1	1	5	4	3	4
178-1	East	3	8	23/24	95.8	3	2	1	8	8	2	3
198	East	3	8	24/24	100.0	4	1	0	8	8	3	4

Table S27. Mean spirochete load of the ear biopsies and necropsies for the 11 strains of *B. burgdorferi*. For each strain, the NML strain ID, geographic region from which the strain was sampled, multi-locus sequence type (MTSL), and number of infected mice are shown. The mouse tissues are the ear biopsies and the 7 necropsies, which include the ventral skin, left ear, right ear, heart, bladder, kidney, and rear tibiotarsal joint. For each strain the spirochete load was calculated as log10[(*23S rRNA*/ 10^6 *Beta actin*) + 1]. The mouse tissue spirochete load (MTSL) refers to the mean of the subset of infected tissues. The MTSL is dominated by the heart and bladder because these tissues have higher spirochete loads than the other necropsies. The MTSL was used as the strain-specific estimate of mouse tissue spirochete load in [5].

Strain	Region	MLST	Mice	Biopsy	Ventral	Ear left	Ear right	Heart	Bladder	Kidney	Joint	MTSL
10-2	Midwest	32	8	3.207	1.456	0.658	0.176	3.524	3.161	0.350	1.909	2.981
22-2	Midwest	55	8	2.965	1.645	1.990	0.521	3.049	3.022	0.376	3.077	2.897
54	Midwest	741	8	2.978	1.153	1.753	0.526	2.762	2.319	0.152	2.280	2.588
57-1	Midwest	29	8	3.474	1.433	1.842	0.826	3.034	3.227	0.735	2.541	2.780
66	Midwest	237	7	2.496	1.263	0.463	0.000	1.533	2.512	0.460	2.144	2.427
126	Midwest	43	8	3.203	2.773	1.738	0.849	3.572	2.813	0.926	2.724	2.746
150	East	19	8	3.204	1.733	0.282	0.138	3.537	3.380	1.069	1.143	3.041
167	East	12	8	3.068	1.486	0.789	0.134	3.408	3.064	0.931	1.684	2.929
174	East	37	5	3.221	2.006	0.926	0.454	3.688	2.798	1.343	1.998	3.059
178-1	East	3	8	3.218	1.465	0.800	0.256	3.426	3.403	0.825	1.378	3.015
198	East	3	8	3.369	1.689	0.260	0.000	3.508	3.483	0.895	1.181	3.085

Table S28. Transmission variables for the 11 strains of *B. burgdorferi*. The 11 *B. burgdorferi* strains, the geographic region from which the strains were sampled, and their multi-locus sequence types (MLST) are shown. For each strain, the number of infected mice, number of infected larvae per total larvae tested, larval infection prevalence (LIP), larval spirochete load 1 (LSL) larval spirochete load 2 (LSL2), nymphal infection prevalence (NIP), nymphal spirochete load 1 (NSL) and nymphal spirochete load 2 (NSL2) are shown. The LSL and NSL are calculated as log10(*23S rRNA* copies + 1) and includes the uninfected ticks, which have a value of 0. The LSL2 and NSL2 are calculated as log10(*23S rRNA* copies) and excludes the uninfected ticks.

Strain	Region	MLST	Mice	Infected/ Total Larvae	LIP (%)	LSL	LSL2	Infected/ Total Nymphs	NIP (%)	NSL	NSL2
10-2	Midwest	32	8	62/87	71.3	2.456	3.532	202/234	86.3	3.806	4.382
22-2	Midwest	55	8	53/83	63.9	2.100	3.522	176/226	77.9	3.433	4.342
54	Midwest	741	8	33/93	35.5	1.001	3.120	155/230	67.4	2.822	4.346
57-1	Midwest	29	8	70/90	77.8	2.699	3.698	212/230	92.2	3.728	4.041
66	Midwest	237	7	27/77	35.1	1.145	3.572	114/210	54.3	2.092	3.887
126	Midwest	43	8	58/90	64.4	2.197	3.571	210/236	89.0	3.825	4.274
150	East	19	8	61/94	64.9	1.892	3.107	205/239	85.8	3.389	3.951
167	East	12	8	66/93	71.0	2.440	3.565	200/240	83.3	3.453	4.141
174	East	37	5	47/58	81.0	3.109	4.008	148/150	98.7	4.763	4.827
178-1	East	3	8	85/89	95.5	3.854	4.194	233/236	98.7	4.672	4.730
198	East	3	8	83/96	86.5	3.265	3.924	229/240	95.4	4.570	4.790

Section 12 - References

1. Tyler S, Tyson S, Dibernardo A, Drebot M, Feil EJ, Graham M, et al. Whole genome sequencing and phylogenetic analysis of strains of the agent of Lyme disease *Borrelia burgdorferi* from Canadian emergence zones. Scientific Reports. 2018;8. doi: 10.1038/s41598-018-28908-7. PubMed PMID: WOS:000438343600062.

2. Margos G, Gatewood AG, Aanensen DM, Hanincova K, Terekhova D, Vollmer SA, et al. MLST of housekeeping genes captures geographic population structure and suggests a European origin of *Borrelia burgdorferi*. Proc Natl Acad Sci U S A. 2008;105(25):8730-5. doi: 10.1073/pnas.0800323105. PubMed PMID: WOS:000257185700045.

3. Wang G, Liveris D, Mukherjee P, Jungnick S, Margos G, Schwartz I. Molecular typing of *Borrelia burgdorferi*. Current protocols in microbiology. 2014;34:12C.5.1-C.5.31. doi: 10.1002/9780471729259.mc12c05s34. PubMed PMID: MEDLINE:25082003.

4. Margos G, Binder K, Dzaferovic E, Hizo-Teufel C, Sing A, Wildner M, et al. PubMLST.org - The new home for the *Borrelia* MLSA database. Ticks Tick Borne Dis. 2015;6(6):869-71. doi: 10.1016/j.ttbdis.2015.06.007. PubMed PMID: WOS:000362143800024.

5. Zinck CB, Thampy PR, Rego ROM, Brisson D, Ogden NH, Voordouw M. *Borrelia burgdorferi* strain and host sex influence pathogen prevalence and abundance in the tissues of a laboratory rodent host. Mol Ecol. 2022;n/a(n/a). doi: https://doi.org/10.1111/mec.16694.

6. Barbour AG, Travinsky B. Evolution and distribution of the *ospC* gene, a transferable serotype determinant of *Borrelia burgdorferi*. mBio. 2010;1(4). doi: 10.1128/mBio.00153-10. PubMed PMID: WOS:000284718000007.

7. Wang IN, Dykhuizen DE, Qiu W, Dunn JJ, Bosler EM, Luft BJ. Genetic diversity of *ospC* in a local population of *Borrelia burgdorferi* sensu stricto. Genetics. 1999;151:15-30.

8. Bunikis J, Garpmo U, Tsao J, Berglund J, Fish D, Barbour AG. Sequence typing reveals extensive strain diversity of the Lyme borreliosis agents *Borrelia burgdorferi* in North America and *Borrelia afzelii* in Europe. Microbiology-Sgm. 2004;150:1741-55. doi: 10.1099/mic.0.26944-0. PubMed PMID: WOS:000222437300016.

9. R Development Core Team. R: A language and environment for statistical computing. Vienna, Austria: R Foundation for Statistical Computing; 2021.

10. RStudio Team. RStudio: Integrated Development for R. Boston, MA: RStudio, PBC; 2020.

11. Bates D, Machler M, Bolker BM, Walker SC. Fitting Linear Mixed-Effects Models Using lme4. Journal of Statistical Software. 2015;67(1):1-48. doi: 10.18637/jss.v067.i01. PubMed PMID: WOS:000365981400001.

12. Fox J, Weisberg S. An R Companion to Applied Regression. Third edition ed. Thousand Oaks, CA: Sage; 2019.

13. Lenth RV. Emmeans: Estimated marginal means, aka least-squares means. R package version 1.5-2.1. 2021.

14. Lüdecke D. ggeffects: Tidy Data Frames of Marginal Effects from Regression Models. Journal of Open Source Software. 2018;3(26). doi: https://doi.org/10.21105/joss.00772.

15. Harrell FEJ, Dupont C. Hmisc: Harrell Miscellaneous. R package version 4.4-2 ed2020.

16. Courtney JW, Kostelnik LM, Zeidner NS, Massung RF. Multiplex real-time PCR for detection of *Anaplasma phagocytophilum* and *Borrelia burgdorferi*. J Clin Microbiol. 2004;42(7):3164-8. doi: 10.1128/jcm.42.7.3164-3168.2004. PubMed PMID: WOS:000222672100046.

17. Torres G, Hoehmann CL, Cuoco JA, Hitscherich K, Pavia C, Hadjiargyrou M, et al. Ketamine intervention limits pathogen expansion in vitro. Pathogens and Disease. 2018;76(2). doi: 10.1093/femspd/fty006. PubMed PMID: WOS:000439773200005.

18. Couret J, Dyer MC, Mather TN, Han S, Tsao JI, Lebrun RA, et al. Acquisition of *Borrelia burgdorferi* infection by larval *Ixodes scapularis* (Acari: Ixodidae) associated with engorgement measures. J Med Entomol. 2017;54(4):1055-60. Epub 2017/04/12. doi: 10.1093/jme/tjx053. PubMed PMID: 28399208.

19. Ogden NH, Margos G, Aanensen DM, Drebot MA, Feil EJ, Hanincova K, et al. Investigation of genotypes of *Borrelia burgdorferi* in *Ixodes scapularis* ticks collected during surveillance in Canada. Appl Environ Microbiol. 2011;77(10):3244-54. doi: 10.1128/aem.02636-10. PubMed PMID: WOS:000290473200011.

20. Travinsky B, Bunikis J, Barbour AG. Geographic differences in genetic locus linkages for *Borrelia burgdorferi*. Emerg Infect Dis. 2010;16(7):1147-50. doi: 10.3201/eid1607.091452. PubMed PMID: WOS:000279522200019.
